# Copper-azide nanoparticle: a ‘catalyst-cum-reagent’ for the designing of 5-alkynyl 1,4-disubstituted triazoles

**DOI:** 10.1038/s41598-020-74018-8

**Published:** 2020-10-07

**Authors:** Debkumar Nandi, Venkata K. Perla, Sarit K. Ghosh, Charmaine Arderne, Kaushik Mallick

**Affiliations:** 1 Department of Chemical Sciences, University of Johannesburg, P.O. Box 524, Auckland Park, 2006 South Africa; 2 Present Address: TCG Lifesciences Pvt. Limited, Plot No-7, Salt Lake Electronics Complex, BN Block, Sector V, Kolkata, West Bengal 700091 India

**Keywords:** Catalysis, Organic chemistry

## Abstract

A single pot, wet chemical route has been applied for the synthesis of polymer supported copper azide, CuN_3_, nanoparticles (CANP). The hybrid system was used as ‘catalyst-cum-reagent’ for the azide-alkyne cyclo-addition reaction to construct triazole molecules using substituted benzyl bromide and terminal alkyne. The electron donating group containing terminal alkyne produced 5-alkynyl 1,4-disubstituded triazole product whereas for alkyne molecule with terminal electron withdrawing group facilitate the formation of 1,4-disubstituted triazole molecule.

## Introduction

The copper catalyzed 1,3-dipolar cycloaddition of azides and terminal alkynes is considered to be the most protuberant reaction of the Click chemistry concept^1^ to construct 1,2,3-triazole moieties, widely applicable in pharmaceutical, combinatorial, and material chemistry^2–5^. Catalytic conversion of the same reaction using copper as catalyst leads to 1,4 substituted triazole as sole product^6–8^. Reaction between sodium, lithium, or magnesium acetylides with organic azides^9^ in presence of ruthenium catalyst^10^ have also been reported where 1,5-disubstituted 1,2,3-triazoles are the major products. Coinage metals, such as, silver^11,12^ and gold^13^ have also appeared as catalyst for the construction of 1,4 substituted triazole molecules. The azide-alkyne cycloaddition reaction between carbon nanotube functionalized azide containing polymer and alkyne molecule has been reported using copper acetate as an efficient catalyst^14^. A Cu(I)-N‐heterocyclic carbene framework has been applied for the azide-alkyne cycloaddition reaction and it was established that six‐membered NHC ligands showed superior performance for the preparation of 1,4‐disubstituted triazoles as compared with the five‐membered one^15^. A Cu(I)‐catalyzed three component (terminal alkynes, azides, and propargylic carbonate) reaction exhibited the formation of 1,3,5‐trisubstituted triazoles (5‐allenyl‐1,2,3‐triazoles) with a high yield^16^. An efficient one pot synthesis protocol has been reported for the copper-mediated azide-alkyne cycloaddition using alkenyl-triflate as a precursor^17^.

Though significant progresses have been made in azide-alkyne cyclo addition reactions but most of them are restricted to the construction of either 1,4 or 1,5-disubstituted-1,2,3-triazole moieties. Few information are also available for the specific synthesis of 1,4,5-trisubstituted 1,2,3-triazole moieties and with much scope to be explored yet. The assembling of 1,4,5-trisubstituted triazoles has been reported using Cu(CH_3_CN)_4_ PF_6_ as catalyst, substituted ethylene diamine as ligand in presence of molecular oxygen as oxidant and 4-methoxymorpholine N-oxide as co-oxidant^18^. Temperature controlled synthesis of bis-(1,2,3-triazole) and 5-alkynyl-1,2,3-triazole has also been reported from various alkyne and azide molecules^19^.

Last few years, we also have engaged in the search of effective catalysts for different organic transformations reactions^20–26^. In association with our ongoing research on the development of effective catalysts for the azide-alkyne cycloaddition reaction, a single pot approach was reported where in-situ catalyst generation and azide-alkyne cyclo product formation was discussed^27^. Cu(I)-polyaminobenzoic acid catalyzed azide-alkyne cycloaddition reaction^28^ and solvent-less microwave irradiation technique using polymer supported copper(I) composite for triazole formation reaction^29^ have been reported earlier. Role of photonic quantum dot (Cu_2_S) as catalyst^30^ and photonic effect of carbon nitride on the catalytically active Cu center^31^ for 1,2,3-triazole formation have also been reported recently by our group.

In this current communication, we have describe a step-wise route for the synthesis of copper azide nanoparticles (CANP) and applied this material as ‘catalyst cum reagent’ where Cu(I) was performed as a catalytic role and azide counterpart acted as nucleophilic role for azide-alkyne cyclo-addition reaction to construct triazole molecules.

## Experimental

### Synthesis of copper azide nanoparticles (CANP)

In a typical experiment, 0.093 g of aniline (10^−2^ mol/dm^3^) was diluted in 10 mL methanol in a conical flask. To this solution, 10 mL of aqueous CuSO_4_, 5H_2_O solution (10^−2^ mol/dm^3^) was added slowly under continuous stirring conditions. During the addition, the solution turned to green, while at the end, a green coloured precipitation was formed at the bottom of the conical flask. To the above precipitation, 5 mL aqueous solution of sodium azide (0.065 g) was added drop-wise and allowed to stir for another 2 h. A change in colour from light green to dark green was noticed. Little amount of the precipitated material was collected from the bottom of the conical flask and pipetted onto lacey, carbon-coated, nickel mesh grids for transmission electron microscopy (TEM) study, before and after the addition of sodium azide. The remaining portion of the compound was dried under vacuum at 60 °C and used as a catalyst for the title reaction.

## Result and discussion

### Mechanism for the formation of copper azide nanoparticles (CANP)

**Figure 1:**
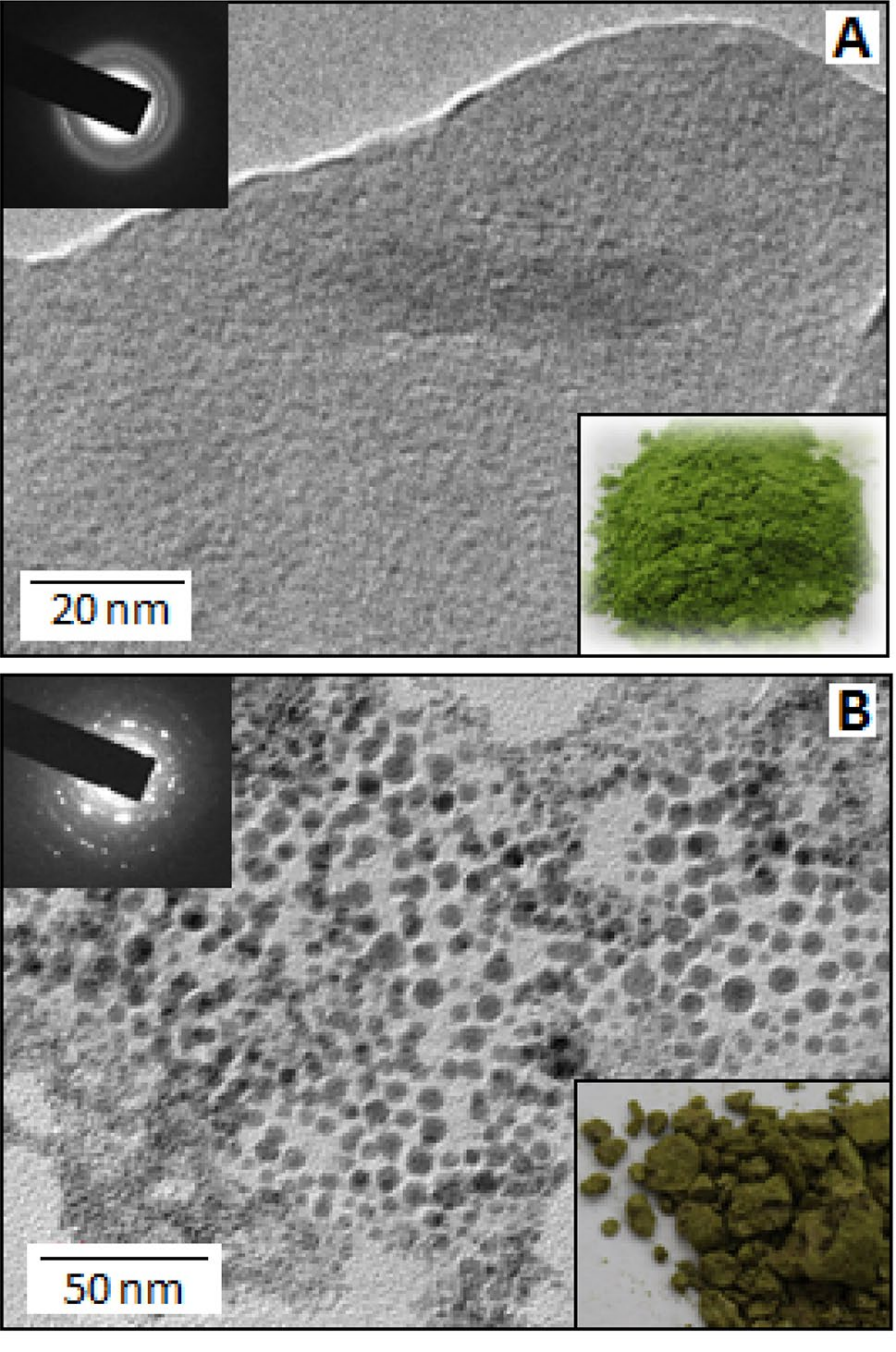
(**A**) TEM image of Cu-polyaniline. Inset: Selected area diffraction and optical images of Cu-polyaniline. (**B**) TEM image of CuN_3_-polyaniline. Inset: Selected area diffraction and optical images of CuN_3_-polyaniline.

Metal salt of gold, silver and palladium mediated synthesis of polyaniline, from aniline monomer, involve the release of electrons during the reaction, where the metal salts act as an oxidizing agents. The released electrons reduce the metal ions with the formation of the corresponding nanoparticles and the oxidation of aniline forms polyaniline, act as a stabilizer for the particles^32–34^. In the current experiment, during the reaction between cupric sulphate and aniline evidenced the formation of Cu(I)-polyaniline, due to the partial reduction of Cu(II)^27,28^. Polyaniline have several amine and imine moieties which can act as a macro ligand^35^, that coordinate with the Cu(I) species. The Cu(I)-polyaniline subsequently forms polyaniline stabilized CuN_3_ nanoparticles by the addition of sodium azide. Figure 1 (A and B) display the TEM images of the organic–inorganic composite system before and after the addition of sodium azide, respectively. The selected area electron diffraction (SAED) image (Fig. 1, inset) indicates highly crystalline nature of CuN_3_-polyaniline system. Figure 1, insets, show the optical images of Cu(I)-polyaniline and CuN_3_-polyaniline hybrid system. The TEM image (Fig. 1B) shows the formation of copper-azide nanoparticles (dark spots) with the size distribution ranging from 5 to 12 nm.

### Application of CANP for triazole synthesis

Initially, we had optimize the reaction condition and for the first set of reaction, 1.0 equivalent of each benzyl bromide (**1a**), 4-ethynyl-1-fluoro-2-methylbenzene (**2e**), CANP and tri-ethylamine were mixed together in methanol under stirring condition at 25 °C. The reaction was monitored by thin-layer chromatography technique and two distinguished spots, other than starting materials, were observed under UV-irradiation. After 3 h, the products were isolated and characterized by spectroscopic techniques. The isolated yield resulted 50% of the 1,4 disubstituted-triazole [1-benzyl-4-(4-fluoro-3-methylphenyl)-1*H*-1,2,3-triazole], **3ae′**, and 30% of the 5-alkynyl-1,4-disubstituded-triazole [1-benzyl-4-(3-fluoro-4-methylphenyl)-5-((3-fluoro-4-methylphenyl)ethynyl)-1*H*-1,2,3-triazole], **3ae (**supplementary information, Table S1). For a second set of reaction, 1.0 equivalent of benzyl bromide and CANP, 2.0 equivalent of alkyne and tri-ethylamine produced **3ae,** with the yield of 85%, and no side product (**3ae′)** was obtained (Table S1, entry 7). Among the various solvents tested, methanol and ethanol showed identical efficiency and the rest of the study was performed using methanol as a sole solvent. The effect of various bases, such as, hydrazine hydrate, di-/tri-ethylamine, potassium carbonate and potassium hydroxide were tested for the reaction. The triethylamine showed the best performance and was applied for the rest of the studies.

**Figure 2:**
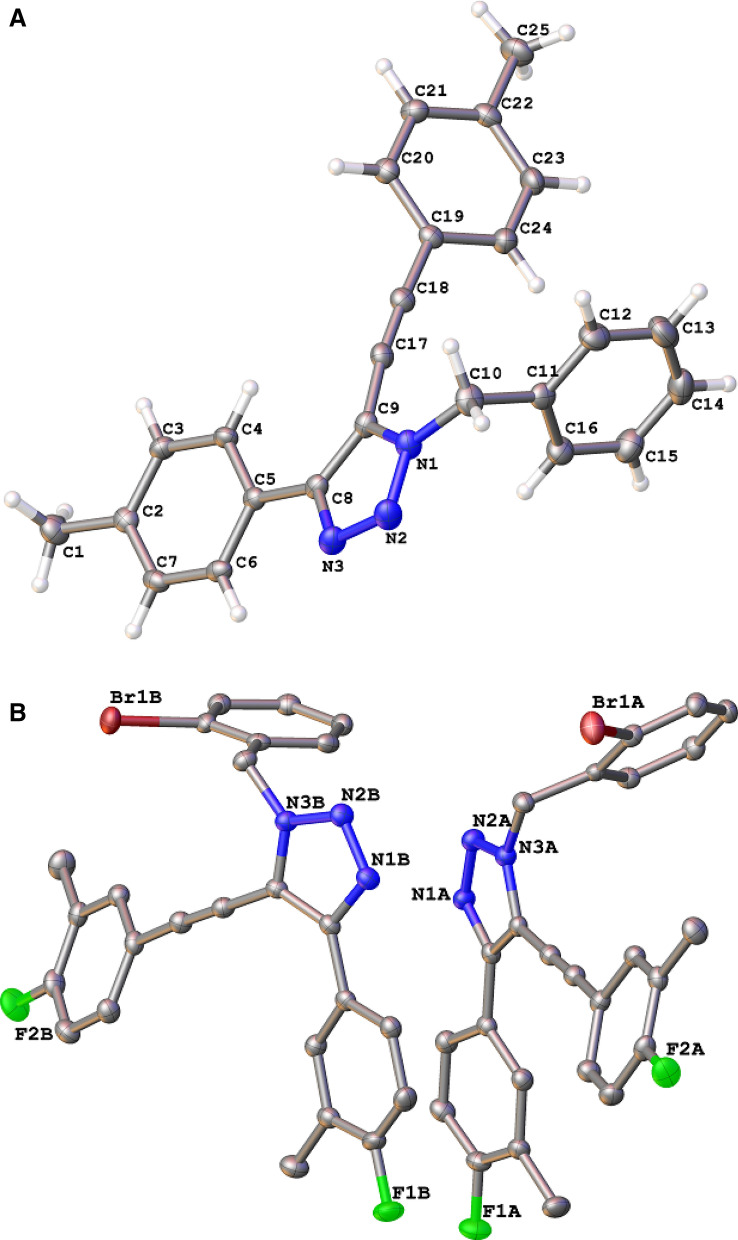
(**A**) The single crystal structure of 1-benzyl-4-(4-methoxyphenyl)-5-((4-methoxyphenyl) ethynyl)-1*H*-1,2,3-triazole, **3ac**. (**B**) The single crystal structure of 1-(2-bromobenzyl)-4-(3-fluoro-4-methylphenyl)-5-((3-fluoro-4-methylphenyl) ethynyl)-1*H*-1,2,3-triazole, **3be**.

To study the general applicability and the substrate scope of the above reaction, a series of benzyl bromide based molecules and terminal alkynes were investigated. The resultant compounds and the corresponding yield (in terms of %) are incorporated in Table 1. In the presence of CANP, benzyl bromide (**1a**), *2*-bromo-benzyl bromide (**1b**) and *4*-methyl-benzyl bromide (**1c**) reacted with phenylacetylene (**2a**) produced 5-alkenyl triazole products, such as, 1-benzyl-4-phenyl-5-(phenylethynyl)-1*H*-1,2,3-triazole (**3aa**), 1-(2-bromobenzyl)-4-phenyl-5-(phenylethynyl)-1*H*-1,2,3-triazole) (**3ba**) and 1-(4-methylbenzyl)-4-phenyl-5-(phenylethynyl)-1*H*-1,2,3-triazole (**3ca**), respectively. Under the above menttioned condotions, mono substituted aromatic tramninal alkyne, such as, 1-ethynyl-4-methylbenzene (**2b**) and 1-ethynyl-4-methoxybenzene (**2c**) successfully reacted with benzyl bromides (**1a**, **1b** and **1c**). The resultant products **3ab**, **3bb**, **3cb**, **3ac** and **3bc** were formed in the range of 89–97% of yield. Disubstituted (*o–p* and *m–p*) aromatic terminal alkynes, 1-ethynyl-4-methoxy-2-methylbenzene (**2d)** and 4-ethynyl-1-fluoro-2-methylbenzene (**2e**), reacted with benzyl bromide (**1a**), *2*-bromo-benzyl bromide (**1b**) and *4*-methyl-benzyl bromide (**1c**) and produced 5-alkynyl-1, 4-disubstituded-triazole products, **3ad**, **3bd**, **3cd**, **3ae**, **3be** and **3ce**, respectively (Table 1). The single crystal structures of 1-benzyl-4-(4-methoxyphenyl)-5-((4-methoxyphenyl) ethynyl)-1*H*-1,2,3-triazole, **3ac**, and 1-(2-bromobenzyl)-4-(3-fluoro-4-methylphenyl)-5-((3-fluoro-4-methylphenyl)ethynyl)-1*H*-1,2,3-triazole, **3be**, are presented in Fig. 2, A and B, respectively. Ether linked terminal alkynes, [(prop-2-ynyloxy) methyl] benzene (**2f**) and 1-bromo-2-[(prop-2-ynyloxy) methyl] benzene (**2g**), have also responded on the title reaction with benzyl bromide (**1a**) or *2*-bromo-benzyl bromide (**1b**) to produce 5-alkynyl-1,4-di-substituded triazole molecules such as, 1-benzyl-5-[3-(benzyloxy) prop-1-ynyl]-4-(benzyloxymethyl)-1*H*-1,2,3-triazole (**3af**), 5-[3-(benzyloxy) prop-1-ynyl]-4-(benzyloxymethyl)-1-(2-bromobenzyl)-1*H*-1,2,3-triazole, (**3bf**), 1-benzyl-4-[(2-bromobenzyloxy)methyl]-5-[3-(2-bromobenzyloxy)prop-1-ynyl]-1*H*-1,2,3triazole **(3ag**) and 1-(2-bromobenzyl)-4-[(2-bromobenzyloxy) methyl]-5-(3-(2-bromobenzyloxy) prop-1-ynyl)-1*H*-1,2,3-triazole (**3bg**), with the isolated yields ranging from 81–86%. Benzyl bromide based molecules (**1a**, **1b** and **1c**) formed **3ah**, **3bh** and **3ch** with the isolated yield of 90, 93 and 96%, respectively, when reacted with 2-ethynyl-6-methoxynaphthalene (**2h**). Aliphatic terminal alkyne, hex-1-yne (**2i**), has also participated with **1a**, **1b** and **1c** and resulted the expected products 1-benzyl-4-butyl-5-(hex-1-ynyl)-1*H*-1,2,3-triazole (**3ai**), 1-(2-bromobenzyl)-4-butyl-5-(hex-1-ynyl)-1*H*-1,2,3-triazole (**3bi**) and 4-butyl-5-(hex-1-ynyl)-1-(4-methylbenzyl)-1*H*-1,2,3-triazole (**3ci**) with the isolated yield of 87, 85 and 91%, respectively.

**Table 1:**
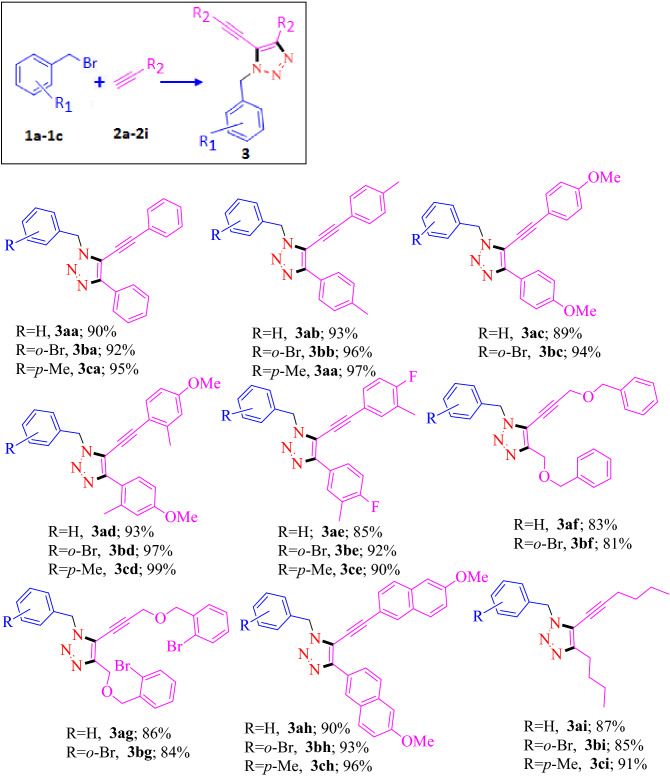
Substrate scope for the reaction.

Reaction condition: Aryl bromide (1.0 eqv), CANP (1.0 eqv 200 mg), Terminal alkyne (2.0 eqv), MeOH (4 mL), Et_3_N (2 eqv), 25 °C, 3 h. Isolated yield.

On the other hand, the reaction between terminal alkyne, attached with the electron withdrawing group, and benzyl bromide based molecules resulted 1, 4-disubstituted 1, 2, 3-triazole as the sole product (Table 2). Electron withdrawing group attached terminal alkyne, such as, 1-ethynyl-4-nitrobenzene (**2j**) when reacted separately with two equivalent of each benzyl bromide (**1a**) and *2*-bromo-benzyl bromide (**1b**), in presence of CANP, only one equivalent of molecule has taken part in the reaction and produced 1-benzyl-4-(4-nitrophenyl)-1*H*-1,2,3-triazole (**4aj**) and 1-(2-bromobenzyl)-4-(4-nitrophenyl)-1*H*-1,2,3-triazole (**4bj**), as the sole product with 83 and 87% of isolated yield, respectively. In a **s**imilar fashion, 1-ethynylcyclohexanol (**2k)** and prop-2-yn-1-ol (**2l)** also reacted individually with **1a** and **1b** and formed 1, 4-disubstituted 1,2,3-triazole (**4ak**, **4bk**, **4al** and **4bl**) as end product.

**Table 2:**
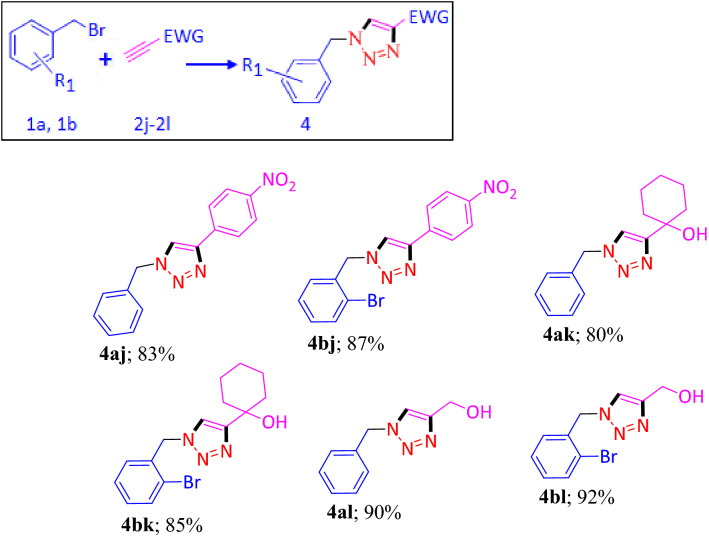
Substrate scope for electron withdrawing group attached terminal alkynes.

Reaction condition: Aryl bromide (1.0 eqv.), CANP (1.0 eqv. 200 mg), Terminal alkyne (1.0 eqv.), MeOH (4 mL), Et_3_N (1.0 eqv.), 25 °C, 3 h. Isolated yield.

We also have performed the following experiments to find out the exclusivity of CANP for the title reaction. A Cu(I)-supported polyaniline (Cu-PANI), as reported earlier^27^, has been prepared and applied in the present reaction system under optimized condition, where, we observed only alkyne 2b and 2c yielded 5-alkynyl 1,4-disubstituded triazoles products (3) in minute amount, 4% and 7%, respectively, and 1,4-disubstituted triazole molecule (3′) formed as the major product (Table 3).

**Table 3:** Yield comparison of triazole molecule using Cu-PANI and CANPS catalysts.


Entry	Catalyst	Product 1 (3, %)	Product 1 (3′, %)
1	Cu-PANI	3aa, 0	3aa′, 99
2	CANP	3aa, 90	3aa′, 0
3	Cu-PANI	3ab, 4	3ab′, 90
4	CANP	3ab, 93	3ab′, 0
5	Cu-PANI	3ac, 7	3ac′, 89
6	CANP	3ac, 89	3ac′, 0
7	Cu-PANI	3ae, 0	3ae′, 87
8	CANP	3ae, 85	3ae′, 0

Reaction Conditions: Benzyl bromide (171 mg, 1.0 mmol), 4-ethynyl-1-fluoro-2-methylbenzene, MeOH (4.0 mL), Cu-PANI/CANP (200 mg, 1 equivalent), 3 h. Isolated yields.

**Figure 3:**
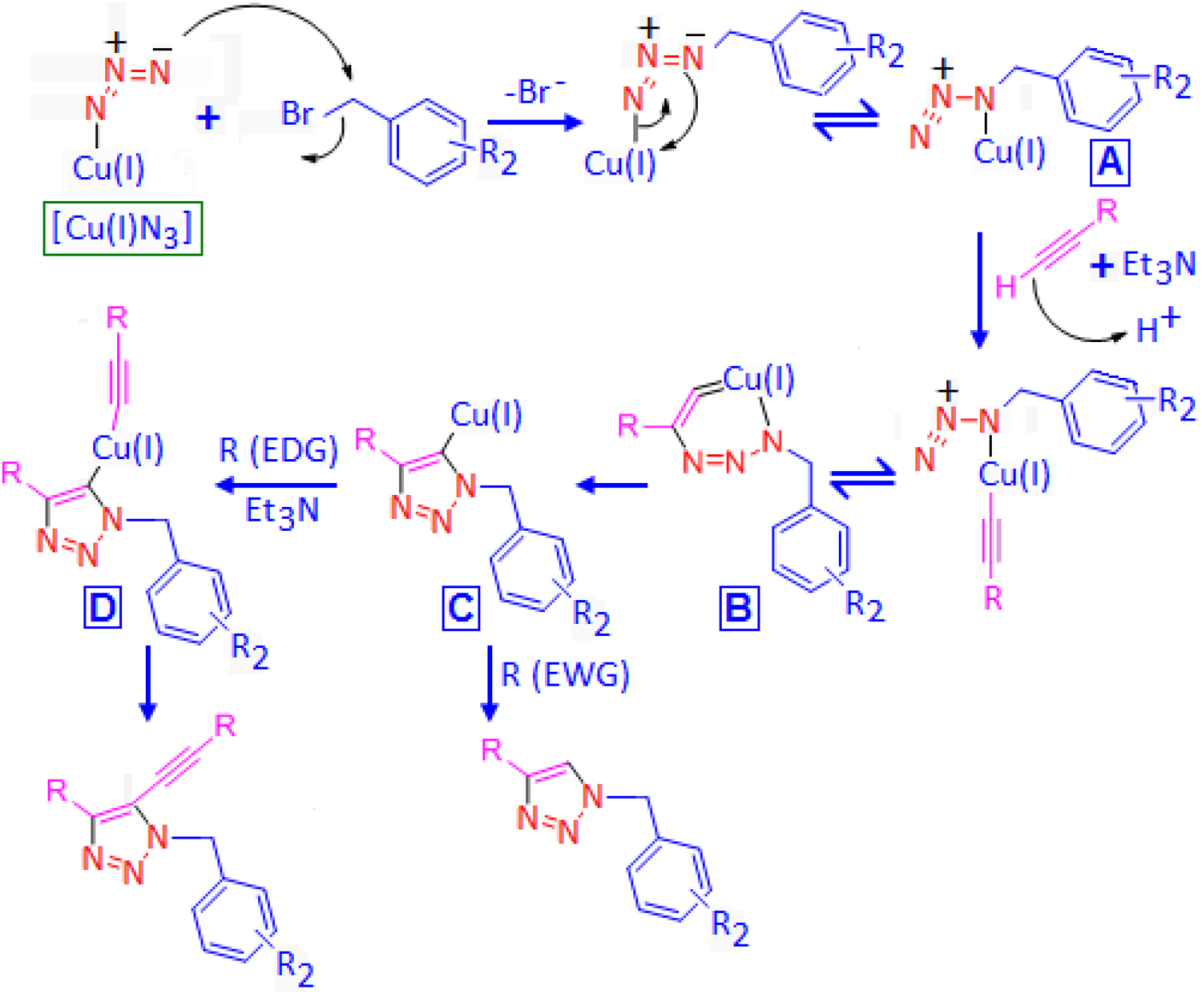
Proposed mechanism for CANP mediated azide-alkyne cyclo addition reaction using alkyne molecule with electron donating group (EDG) and electron withdrawing group (EWG).

The mechanism of the above observations can be explained as follows. Initially, benzyl bromide molecule followed an aromatic nucleophilic substitution reaction and formed copper-organic azide complex, which further rearranged to its canonical conjugate form (Fig. 3, intermediate A). In presence of triethylamine, a facile deprotonating of the terminal hydrogen of the alkyne molecule allows to the formation of Cu-acetylidine complex that subsequently rearranged and produced metallacycle (Fig. 3, intermediate B). In the next step, the formation of 1,4-disubstituted triazole unit attached with copper center at 5-position (Fig. 3, intermediate C). In case of electron donating group (EDG) containing terminal alkyne, further deprotonation resulted to second Cu-acetylidine complex D (Fig. 3), which finally produced 5-alkenyl 1,4-disubstituted triazole, as an end product. On the other hand, for alkyne molecule with terminal electron withdrawing group (EWG), a strong rate limiting oxidative addition facilitate the formation of 1,4-disubstituted triazole molecule.

In all the above reactions, the ‘reagent’ (azide) counterpart of the ‘catalyst-cum-reagent’ system (CANP) was drained out to form the products. The recovered catalyst (RC), which is left with the copper component, was applied for the preparation of triazole glycoside, where the reaction between sugar azide and terminal alkyne formed the 1, 4-disubstituted triazole. Various di- and mono-substituted aromatic terminal alkynes, such as, 1-ethynyl-4-methoxy-2-methylbenzene (**2d**), 4-ethynyl-1-fluoro-2-methylbenzene (**2e**) and 1-ethynyl-4-nitrobenzene (**2j**) reacted with sugar azide molecules [1-azido-2,3,4,6-tetra-*o*-acetyl-β-D-glucopyranose (**5a**) and 1-azido-2,3,4,6-tetra-*o*-acetyl-β-D-galactopyranose **(5b)**] and formed 1, 4 disubstituted triazole molecules (**6ad**, **6bd**, **6ae**, **6be**, **6aj** and **6bj**), within the range of 73–90% of product yield (Table 4).

**Table 4:**
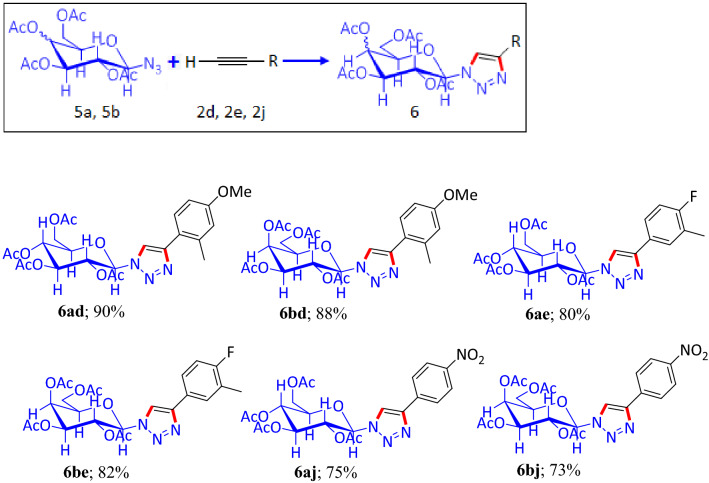
Substrate scope for sugar azide and terminal alkyne and recovered catalyst (RC).

Reaction condition: Aryl bromide (1 eqv), RC (10 mg), alkyne (1 eqv), MeOH (4 mL), Et_3_N (1 eqv), 25 °C, 3 h. Isolated yield.

In this communication, a facile route was adopted for the preparation of polyaniline stabilized copper-azide nanoparticle. The copper azide nanoparticle was performed as ‘catalyst-cum-reagent’ for synthesizing the triazole molecules through azide-alkyne cyclo-addition reaction using substituted benzyl bromide and terminal alkyne molecules, where copper was performed as a role of catalyst for the cyclo-addition reaction and azide was the source of triazole unit. The alkyne with electron donating group was produced 5-alkynyl 1,4-disubstituded triazoles as the sole product, whereas, alkyne molecule with terminal electron withdrawing group facilitate the formation of 1,4-disubstituted triazole molecule. The recovered catalyst (without azide counterpart) showed the catalytic performance for the reaction between sugar azide and terminal alkyne with the formation of triazole glycosides.

## Methods

### General procedure for the click reaction

In a 25 mL round bottom flask, substituted benzyl bromide **1** (1.0 equivalent), terminal alkyne **2** (2.0 equivalent or 1.0 equivalent for EWG attached alkyne), were mixed in 4 mL of solvent (methanol). To this reaction mixture, CANP (200 mg, 1.0 equivalent) and Et_3_N (240 µL, 2.0 equivalent or 140 µL, 1.0 equivalent for EWG attached alkyne) was added and allowed to stir for 3 h. The reaction mixture was monitored using a thin layer chromatography technique. After complete disappearance of starting materials, a previously reported technique^23^ was followed to purify the the triazole products.

### General procedure for click reaction with recovered catalyst

In a 25 mL round bottom flask, sugar azide **5** (1 equivalent), terminal alkyne **2** (1 equivalent), were charged in 4 mL methanol. To this reaction mixture the dried recovered catalyst (10 mg) and Et_3_N (1 equivalent) was added and allowed to stir for 3 h. The reaction mixture was monitored using thin layer chromatography technique. After completion, the reaction mixture was filtered and dried under residue pressure and followed the similar procedure as above.

### Single crystal analysis

#### Computing details

Data collection: APEX2 2014-11; cell refinement: SAINT v8.38A; data reduction: SAINT v8.38A; program used to solve structure: SHELXT 2014/5; program used to refine structure: SHELXL 2018/3; molecular graphics: Olex2; software used to prepare material for publication: Olex2, PLATON.

Crystal Data: 1-benzyl-4-(4-methoxyphenyl)-5-[(4-methoxyphenyl) ethynyl]-1*H*-1,2,3-triazole, 3ac, C_25_H_21_N_3_ (M = 363.45 g/mol): monoclinic space group P2_1_/c (no. 14), a = 12.7083(16) Å, b = 5.5633(7) Å, c = 27.879(4) Å, V = 1934.4(4) Å^3^, Z = 4, T = 100.03 K, μ(MoKα) = 0.074 mm^-1^, D_calc_ = 1.248 g/cm^3^, 39,612 reflections measured (2.412 ≤ Θ ≤ 28.565°), 3849 unique (R_int_ = 0.0699, R_sigma_ = 0.0462) which were used in all calculations. The final R_1_ was 0.0499 (I > 2σ (I)) and wR_2_ was 0.1323 (all data).

1-(2-Bromobenzyl)-4-(3-fluoro-4-methylphenyl)-5-[(3-fluoro-4-methylphenyl) ethynyl]-1*H*-1,2,3-triazole, 3be, C_25_H_18_N_3_BrF_2_ (M = 478.33 g/mol): triclinic space group P$\stackrel{-}{1}$ (no. 2), a = 7.4264(6) Å, b = 13.0510(12) Å, c = 21.726(2) Å, V = 2075.3(3) Å^3^, Z = 4, T = 99.96 K, μ(MoKα) = 2.016 mm^-1^, D_calc_ = 1.531 g/cm^3^, 62,912 reflections measured (2.412 ≤ Θ ≤ 28.565°), 10,501 unique (R_int_ = 0.0497, R_sigma_ = 0.0345) which were used in all calculations. The final R_1_ was 0.0349 (I > 2σ (I)) and wR_2_ was 0.0942 (all data).

## Supplementary information

Supplementary information.
