# Supplementary material for: Copper-azide nanoparticle: a ‘catalyst-cum-reagent’ for the designing of 5-alkynyl 1,4-disubstituted triazoles
Source: Sci Rep. 2020 Oct 7;10:16720. doi: 10.1038/s41598-020-74018-8 (PMC7542177; doi:10.1038/s41598-020-74018-8)
Supplement: Supplementary file 1 — Supplementary information. [file 41598_2020_74018_MOESM1_ESM.docx]

**Supporting Information**

**Copper-azide nanoparticle: A ‘catalyst-cum-reagent’ for the designing of 5-alkynyl 1, 4-disubstituded triazoles**

Debkumar Nandi, Venkata K Perla, Sarit K Ghosh, Charmaine Arderne, Kaushik Mallick

Department of Chemical Sciences, University of Johannesburg, P.O. Box: 524, Auckland Park, 2006, South Africa.

**List of contents**

| *General Considerations* | *S1* |
| --- | --- |
| *Optimization of reaction* | *S2* |
| *Characterization cyclo-adduct* | *S3-S16* |
| *Experimental References* | *S16* |
| *NMR Spectra* | *S17-S56* |

**General Considerations:**

Solvents were distilled from appropriate drying agent prior to use. Commercially available reagents were used without further purification unless otherwise stated. ^1^H and ^13^C NMR spectra were recorded on a Bruker AVANCE^III^-400 spectrometer.^1^H NMR (400 MHz) and ^13^C NMR (100 MHz) were registered in CDCl_3_ as solvent and tetramethylsilane (TMS) as an internal standard. Chemical shifts are reported in δ units (ppm). All coupling constants (*J*) are reported in hertz (Hz).

**Table S1:** Optimization of reaction condition ^a^


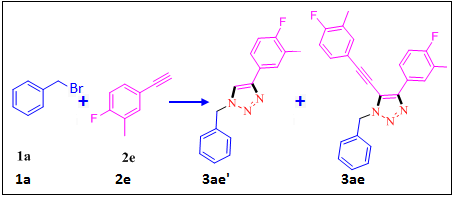


| **Entry** | **CAN**  **(eqv.)** | **Alkyne (eqv.)** | **Et_3_N**  **(eqv.)** | **solvent** | **3ae'**^b^ **(%)** | **3ae** ^b^ **(%)** |
| --- | --- | --- | --- | --- | --- | --- |
| 1 | 1.0 | 1.0 | 1.0 | MeOH | 50 | 30 |
| 2 | 1.2 | 1.0 | 1.0 | MeOH | 50 | 30 |
| 3 | 1.0 | 1.5 | 1.0 | MeOH | 20 | 50 |
| 4 | 1.0 | 2.0 | 1.0 | MeOH | 7 | 70 |
| 5 | 1.0 | 2.2 | 1.0 | MeOH | 7 | 70 |
| 6 | 1.0 | 2.0 | 1.5 | MeOH | 5 | 80 |
| **7** | **1.0** | **2.0** | **2.0** | **MeOH** | **-** | **85** |
| 8 | 1.0 | 2.0 | 2.2 | MeOH | - | 85 |
| 9 | 1.0 | 2.0 | 2.0 | DCM | 10 | 35 |
| 10 | 1.0 | 2.0 | 2.0 | THF | 15 | 25 |
| 11 | 1.0 | 2.0 | 2.0 | MEOH:H2O (1:1) | - | 80 |
| 12 | 1.0 | 2.0 | 2.0 | EtOH | - | 85 |

a) Reaction Conditions: Benzyl bromide (171 mg, 1.0 mmol), 4-ethynyl-1-fluoro-2-methylbenzene, MeOH (4.0 mL), PCA (200 mg, 1 equivalent), 3h. (b) Isolated yields.

***Characterization of cyclo-adduct:***

 **1-benzyl-4-phenyl-5-(phenylethynyl)-1H-1,2,3-triazole (3aa)**:^1^ White solid, (yield: 320 mg, 90%). Synthesized following the general procedure from benzyl bromide **1a** (171 mg, 1.0 mmol), phnylacetelene **2a** (204 mg, 2.0 mmol) and CuN_3_ (PCUA) (200 mg, 1 equivalent). ^1^H NMR (400 MHz, CDCl_3_): 5.66 (s, 2H), 7.31-7.50 (d, *J*= 8.4 Hz, 2H), 8.16-8.18 (m, 2H); ^13^C NMR (100 MHz, CDCl_3_): δ 52.9, 75.5, 102.3, 117.2, 121.4, 126.2 (2C), 128.0 (2C), 128.5, 128.6, 128.8 (4C), 129.6, 130.3(2C), 131.5, 134.7 (2C), 148.4.

**1-(2-bromobenzyl)-4-phenyl-5-(phenylethynyl)-1H-1,2,3-triazole) (3ba)**: Light yellow solid, (yield: 380 mg, 92%). Synthesized following the general procedure from *o*-bromobenzylbromide **1b** (249 mg, 1.0 mmol), phnylacetelene **2a** (204 mg, 2.0 mmol) and CuN_3_ (PCUA) (200 mg, 1 equivalent). ^1^H NMR (400 MHz, CDCl_3_): δ 5.81 (s, 2H), 6.98 (d, *J*= 8.0 Hz, 1H), 7.17-7.18 (m, 1H), 7.19-7.25 (m, 1H), 7.35-7.40 (m, 4H), 7.45-7.50 (m, 4H), 7.60 (d, *J*= 8.0 Hz, 1H), 8.22-8.24 (m, 2H); ^13^C NMR (100 MHz, CDCl_3_): δ 52.3, 75.1, 102.8, 117.8, 121.3, 122.6, 126.2 (2C), 127.9, 128.5, 128.6 (2C), 128.7 (2C), 128.9, 129.6, 129.7, 130.2, 131.5, 132.8 (2C), 134.2, 147.9.

**1-(4-methylbenzyl)-4-phenyl-5-(phenylethynyl)-1*H*-1,2,3-triazole (3ca)**: White solid, (yield: 332 mg, 95%). Synthesized following the general procedure from 1-(bromomethyl)-4-methylbenzene **1c** (185 mg, 1.0 mmol), phnylacetelene **2a** (204 mg, 2.0 mmol) and CuN_3_ (PCUA) (200 mg, 1 equivalent). ^1^H NMR (400 MHz, CDCl_3_): 2.32 (s, 3H), 5.62 (s, 2H), 7.15 (d, *J*= 8.0 Hz, 2H), 7.29-7.33 (m, 3H), 7.40-7.46 (m, 5H), 7.50-7.52 (m, 2H), 8.17 (d, *J*= 7.6 Hz, 2H); ^13^C NMR (100 MHz, CDCl_3_): δ 21.1, 52.8, 75.7, 102.2, 117.1, 121.5, 126.2 (2C), 128.1 (2C), 128.5, 128.6 (2C), 128.7(2C), 129.5 (2C), 129.6, 130.3, 131.5 (2C), 131.7, 138.3, 148.1.

**1-benzyl-4-p-tolyl-5-(p-tolylethynyl)-1H-1,2,3-triazole (3ab):**^1^ White solid, (yield: 338 mg, 93%). Synthesized following the general procedure from benzyl bromide **1a** (171 mg, 1.0 mmol), 1-ethynyl-4-methylbenzene **2b** (232 mg, 2.0 mmol) and CuN_3_ (PCUA) (200 mg, 1 equivalent). ^1^H NMR (400 MHz, CDCl_3_): 2.39 (s, 3H), 2.40 (s, 3H), 5.64 (s, 2H), 7.22 (d, *J*= 7.6 Hz, 2H), 7.28 (d, *J*= 8.0 Hz, 2H), 7.34-7.36 (m, 3H), 7.40-7.42 (m, 4H), 8.12 (d, *J*= 7.6 Hz, 2H); ^13^C NMR (100 MHz, CDCl_3_): δ 21.2, 21.5, 52.7, 75.1, 102.4, 116.9, 118.2, 126.0 (2C), 127.5, 128.0 (2C), 128.3, 128.7, 129.1, 129.2(2C), 129.3(2C), 131.3(2C), 134.7, 138.3, 139.9, 147.9.

**1-benzyl-4-p-tolyl-5-(p-tolylethynyl)-1H-1,2,3-triazole (3bb):** White solid, (yield: 424 mg, 96%). Synthesized following the general procedure *o*-bromobenzylbromide **1b** (249 mg, 1.0 mmol), 1-ethynyl-4-methylbenzene **2b** (232 mg, 2.0 mmol) and CuN_3_ (PCUA) (200 mg, 1 equivalent). ^1^H NMR (400 MHz, CDCl_3_): 2.37 (s, 3H), 2.40 (s, 3H), 5.79 (s, 2H), 6.7 (d, *J*= 7.6 Hz, 1H), 7.15-7.17 (m, 3H), 7.22-7.28 (m, 3H), 7.35 (d, *J*= 8.0 Hz, 2H), 7.60 (d, *J*= 8.0 Hz, 2H), 8.11 (d, *J*= 8.0 Hz, 2H); ^13^C NMR (100 MHz, CDCl_3_): δ 21.3, 21.6, 52.2, 74.6, 102.9, 117.6, 118.3, 122.5, 126.0 (2C), 127.4, 127.9, 128.3, 129.2(2C), 129.3(2C), 129.7, 131.4(2C), 132.8, 134.3, 138.5, 139.9, 147.9.

**1-(4-methylbenzyl)-4-p-tolyl-5-(p-tolylethynyl)-1H-1,2,3-triazole (3cb):** White solid, (yield: 366 mg, 97%). Synthesized following the general procedure 1-(bromomethyl)-4-methylbenzene **1c** (185 mg, 1.0 mmol), 1-(bromomethyl)-4-methylbenzene **1c** (185 mg, 1.0 mmol) and CuN_3_ (PCUA) (200 mg, 1 equivalent). ^1^H NMR (400 MHz, CDCl_3_): 2.31 (s, 3H), 2.37 (s, 3H), 2.40 (s, 3H), 5.60 (s, 2H), 7.14 (d, *J*= 8.0 Hz, 2H), 7.19-7.25 (m, 4H), 7.30 (d, *J*= 8.0 Hz, 2H), 7.40 (d, *J*= 8.0 Hz, 2H), 8.06 (d, *J*= 8.0 Hz, 2H); ^13^C NMR (100 MHz, CDCl_3_): δ 21.1, 21.3, 21.6, 52.6, 75.2, 102.4, 115.0, 116.9, 118.4, 126.0 (2C), 127.5, 128.1, 129.2(2C), 129.3 (2C), 129.4 (2C), 131.3, 131.4, 131.8, 138.2, 138.3, 139.9, 147.9.

**1-benzyl-4-(4-methoxyphenyl)-5-((4-methoxyphenyl)ethynyl)-1H-1,2,3-triazole (3ac):**^1^ White solid, (yield: 371 mg, 89%). Synthesized following the general procedure benzyl bromide **1a** (171 mg, 1.0 mmol), 1-ethynyl-4-methoxybenzene **2c** (264 mg, 2.0 mmol). and CuN_3_ (PCUA) (200 mg, 1 equivalent). ^1^H NMR (400 MHz, CDCl_3_): 3.82 (s, 3H), 3.83 (s, 3H), 5.62 (s, 2H), 6.90 (d, *J*= 8.4 Hz, 2H), 7.00 (d, *J*= 8.8 Hz, 2H), 7.24-7.42 (m, 7H), 8.11 (d, *J*= 8.8 Hz, 2H); ^13^C NMR (100 MHz, CDCl_3_): δ 52.8, 55.2, 55.4, 74.5, 102.2, 113.4, 114.0 (2C), 114.3 (2C), 116.7, 123.1, 127.4 (2C), 128.4 (2C), 128.3, 128.8 (2C), 133.1 (2C), 134.8, 147.6, 159.7, 160.1.

**1-(2-bromobenzyl)-4-(4-methoxyphenyl)-5-((4-methoxyphenyl)ethynyl)-1*H*-1,2,3-triazole (3bc):** White solid, (yield: 445 mg, 94%). Synthesized following the general procedure *o*-bromobenzylbromide **1b** (249 mg, 1.0 mmol), 1-ethynyl-4-methoxybenzene **2c** (264 mg, 2.0 mmol). and CuN_3_ (PCUA) (200 mg, 1 equivalent). ^1^H NMR (400 MHz, CDCl_3_): 3.81 (s, 3H), 3.83 (s, 3H), 5.76 (s, 2H), 6.89 (d, *J*= 8.8 Hz, 2H), 6.94-6.99 (m, 3H), 7.16 (t, *J*= 7.2 Hz, 1H), 7.24 (d, *J*= 7.4 Hz, 1H), 7.38 (d, *J*= 8.8 Hz, 2H), 7.58 (d, *J*= 7.6 Hz, 1H), 8.14 (d, *J*= 8.8 Hz, 2H); ^13^C NMR (100 MHz, CDCl_3_): δ 52.2, 55.3, 55.4, 74.1, 102.7, 113.4, 114.1 (2C), 114.2 (2C), 117.3, 122.5, 123.0, 127.5 (2C), 127.9, 128.4, 129.7, 132.8, 133.2 (2C), 134.4, 147.5, 159.8, 160.6.

**1-benzyl-4-(4-methoxy-2-methylphenyl)-5-((4-methoxy-2-methylphenyl)ethynyl)-1*H*-1,2,3-triazole (3ad):** White solid, (yield: 394 mg, 93%). Synthesized following the general procedure benzyl bromide **1a** (171 mg, 1.0 mmol), 1-ethynyl-4-methoxy-2-methylbenzene **2d** (292 mg, 2.0 mmol) and CuN_3_ (PCUA) (200 mg, 1 equivalent). ^1^H NMR (400 MHz, CDCl_3_): 2.27 (s, 3H), 2.48 (s, 3H), 3.78 (s, 3H), 3.81 (s, 3H), 5.66 (s, 2H), 6.67-6.71 (m, 2H), 6.75-6.82 (m, 2H), 7.26-7.38 (m, 6H), 7.55 (d, *J*= 8.4 Hz, 1H); ^13^C NMR (100 MHz, CDCl_3_): δ 20.9, 21.1, 52.8, 55.2, 55.3, 77.4, 100.6, 110.9, 111.5, 113.5, 115.2, 116.0, 119.4, 122.1, 127.8 (2C), 128.3, 128.8 (2C), 131.1, 133.4, 135.0, 138.9, 142.2, 149.1, 159.7, 160.4.

**1-(2-bromobenzyl)-4-(4-methoxy-2-methylphenyl)-5-((4-methoxy-2-methylphenyl)ethynyl)-1H-1,2,3-triazole** (**3bd**): White solid, (yield: 486 mg, 97%). Synthesized following the general procedure *o*-bromobenzylbromide **1b** (249 mg, 1.0 mmol), 1-ethynyl-4-methoxy-2-methylbenzene **2d** (292 mg, 2.0 mmol) and CuN_3_ (PCUA) (200 mg, 1 equivalent). ^1^H NMR (400 MHz, CDCl_3_): 2.16 (s, 3H), 2.50 (s, 3H), 3.75 (s, 3H), 3.81 (s, 3H), 5.78 (s, 2H), 6.63-6.66 (m, 2H), 6.76-6.86 (m, 3H), 7.13-7.26 (m, 3H), 7.57-7.59 (m, 2H); ^13^C NMR (100 MHz, CDCl_3_): δ 20.7, 21.1, 52.4, 55.2 (2C), 74.5, 101.1, 111.4, 113.4, 115.2, 116.1, 118.5, 120.1, 122.3, 127.9, 128.4, 129.2, 129.6, 131.1, 132.8, 134.5, 139.0, 142.3, 146.3, 148.9, 159.8, 160.4.

**4-(4-methoxy-2-methylphenyl)-5-((4-methoxy-2-methylphenyl)ethynyl)-1-(4-methylbenzyl)-1H-1,2,3-triazole (3cd):** White solid, (yield: 433 mg, 99%). Synthesized following the general procedure 1-(bromomethyl)-4-methylbenzene **1c** (185 mg, 1.0 mmol), 1-ethynyl-4-methoxy-2-methylbenzene **2d** (292 mg, 2.0 mmol) and CuN_3_ (PCUA) (200 mg, 1 equivalent). ^1^H NMR (400 MHz, CDCl_3_): 2.28 (s, 3H), 2.31 (s, 3H), 3.77 (s, 3H), 3.80 (s, 3H), 5.61 (s, 2H), 6.68-6.81 (m, 4H), 7.13-7.15 (m, 2H), 7.27-7.30 (m, 3H), 7.54 (d, *J*= 8.4 Hz, 1H); ^13^C NMR (100 MHz, CDCl_3_): δ 20.9 (2C), 21.1, 52.6, 55.2, 55.3, 77.7, 101.5, 111.0, 111.5, 113.6, 115.2, 116.5, 119.3, 122.2, 127.9 (2C), 129.5 (2C), 131.1, 132.0, 133.4, 138.1, 138.9, 142.2, 148.0, 159.6, 160.4.

**1-benzyl-4-(3-fluoro-4-methylphenyl)-5-((3-fluoro-4-methylphenyl)ethynyl)-1H-1,2,3-triazole (3ae):** White solid, (yield: 339 mg, 85%). Synthesized following the general procedure benzyl bromide **1a** (171 mg, 1.0 mmol), 4-ethynyl-1-fluoro-2-methylbenzene **1e** (268 mg, 2.0 mmol) and CuN_3_ (PCUA) (200 mg, 1 equivalent). ^1^H NMR (400 MHz, CDCl_3_): 2.30 (s, 3H), 2.32 (s, 3H), 5.64 (s, 2H), 7.04-7.06 (m, 3H), 7.29-7.37 (m, 6H), 7.94-8.03 (m, 2H); ^13^C NMR (100 MHz, CDCl_3_): δ 14.4, 14.6, 52.9, 74.9, 101.6, 115.1 (d, *J*_CF_= 32 Hz, 1C), 115.5 (d, *J*_CF_= 92 Hz, 1C), 116.9 (d, *J*_CF_= 68 Hz, 1C), 125.0, 125.2 (d, *J*_CF_= 32 Hz, 1C), 126.0 (d, *J*_CF_ = 72 Hz, 1C), 126.1 (2C), 127.9 (d, *J*_CF_= 68 Hz, 1C), 128.26, 128.46 (2C), 128.83, 129.21, 129.47, 130.8 (d, *J*_CF_= 32 Hz, 1C), 134.7 (d, *J*_CF_= 28 Hz, 1C), 147.38, 160.4 (d, *J*_CF_= 184 Hz, 1C), 163.2 (d, *J*_CF_= 202 Hz, 1C); ^19^F NMR (400 MHz, CDCl_3_): δ -112.4, -116.8.

**1-benzyl-4-(4-fluoro-3-methylphenyl)-1H-1,2,3-triazole(3ae'):** White solid, (obesurved during optimizetion procedure). Synthesized following the general procedure benzyl bromide **1a** (171 mg, 1.0 mmol), 4-ethynyl-1-fluoro-2-methylbenzene **1e** (268 mg, 2.0 mmol) and CuN_3_ (PCUA) (200 mg, 1 equivalent). ^1^H NMR (400 MHz, CDCl_3_): 2.26 (s, 3H), 5.52 (s, 2H), 6.98 (t, *J*= 9.0 Hz, 1H), 7.28-7.34 (m, 5H), 7.51-7.63 (m, 3H); ^13^C NMR (100 MHz, CDCl_3_): δ 14.4, 54.1, 115.2 (d, *J*_CF_= 90.8 Hz, 1C), 119.2, 124.6 (d, *J*_CF_= 32.4 Hz, 1C), 125.2 (d, *J*_CF_= 70.4 Hz, 1C), 126.3 (d, *J*_CF_ = 13.0 Hz, 1C), 128.0 (2C), 128.7 -128.8 (m, 2C), 129.1, 134.6, 147.4, 159.9, 162.4; ^19^F NMR (400 MHz, CDCl_3_): δ -117.9.

**1-(2-bromobenzyl)-4-(3-fluoro-4-methylphenyl)-5-((3-fluoro-4-methylphenyl)ethynyl)-1H-1,2,3-triazole (3be):** White solid, (yield: 439 mg, 92%). Synthesized following the general procedure *o*-bromobenzylbromide **1b** (249 mg, 1.0 mmol), 4-ethynyl-1-fluoro-2-methylbenzene **1e** (268 mg, 2.0 mmol) and CuN_3_ (PCUA) (200 mg, 1 equivalent). ^1^H NMR (400 MHz, CDCl_3_): 2.28 (s, 3H), 2.34 (s, 3H), 5.78 (s, 2H), 6.96-7.02 (m, 2H), 7.06-7.11 (m, 1H), 7.16-7.20 (m, 1H) 7.25-7.30 (m, 3H), 7.61 (d, *J*= 8.0 Hz, 1H), 7.98 (brs, 1H), 8.06 (d, *J*= 6.8 Hz, 1H); ^13^C NMR (100 MHz, CDCl_3_): δ 14.4, 14.6, 52.3, 74.5, 102.0, 115.1-115.71 (m, 2C), 116.9 (d, *J*_CF_= 12 Hz, 1C),, 117.39, 122.53, 125.0-125.5 (m, 1C), 125.6-126.02 (m, 2C), 127.94, 128.8-129.8 (2C), 130.8 ((d, *J*_CF_= 32 Hz, 1C), 132.83, 134.17 (2C), 134.71, 132.77, 147.19, 160.24 (d, *J*_CF_= 184 Hz, 1C), 162.9 (d, *J*_CF_= 204 Hz, 1C); ^19^F NMR (400 MHz, CDCl_3_): δ -116.1, -117.5.

**4-(4-fluoro-3-methylphenyl)-5-((4-fluoro-3-methylphenyl)ethynyl)-1-(4-methylbenzyl)-1H-1,2,3-triazole (3ce):** White solid, (yield: 372 mg, 90%). Synthesized following the general procedure 1-(bromomethyl)-4-methylbenzene **1c** (185 mg, 1.0 mmol), 4-ethynyl-1-fluoro-2-methylbenzene **1e** (292 mg, 2.0 mmol) and CuN_3_ (PCUA) (200 mg, 1 equivalent). ^1^H NMR (400 MHz, CDCl_3_): 2.30 (s, 3H), 2.31 (s, 3H), 2.32 (s, 2H), 5.59 (s, 2H), 7.01-7.07 (m, 3H), 7.14-7.16 (m, 2H), 7.26-7.31 (m, 4H) 7.91-7.94 (m, 1H), 7.99-8.01 (m, 1H); ^13^C NMR (100 MHz, CDCl_3_): δ 14.4, 14.6, 21.1, 52.8, 75.0, 101.5, 115.1-115.97 (m, 2C), 116.74, 117.1 (d, *J*_CF_= 16 Hz, 1C), 124.9-125.2 (m, 1C), 125.8-126.1 (m, 2C), 128.02 (2C), 129.40, 129.4 (2C), 130.8 (d, *J*_CF_= 32 Hz, 1C), 131.68, 134.6, 134.7, 138.31, 147.36, 160.5 (d, *J*_CF_= 216 Hz, 1C), 162.8 (d, *J*_CF_= 232 Hz, 1C); ^19^F NMR (400 MHz, CDCl_3_): δ -112.5, -116.9.

**1-benzyl-5-(3-(benzyloxy)prop-1-ynyl)-4-(benzyloxymethyl)-1*H*-1,2,3-triazole** **(3af):** White solid, (yield: 352 mg, 83%). Synthesized following the general procedure benzyl bromide **1a** (171 mg, 1.0 mmol), ((prop-2-ynyloxy)methyl)benzene **2f** (292 mg, 2.0 mmol) and CuN_3_ (PCUA) (200 mg, 1 equivalent). ^1^H NMR (400 MHz, CDCl_3_): 4.39 (s, 2H), 4.53 (s, 2H), 4.60 (s, 2H), 4.66 (s, 2H), 5.55 (s, 2H), 7.24-7.36 (m, 15H); ^13^C NMR (100 MHz, CDCl_3_): δ 52.8, 57.5, 62.4, 71.1, 71.8, 72.3, 98.6, 119.9, 127.6, 127.8 (2C), 127.9 (2C), 128.0 (2C), 128.1, (2C), 128.3(2C), 128.4 (2C), 128.8 (2C), 134.4, 136.8, 137.7, 147.3.

**5-(3-(benzyloxy)prop-1-ynyl)-4-(benzyloxymethyl)-1-(2-bromobenzyl)-1H-1,2,3-triazole (3bf):** White solid, (yield: 407 mg, 81%). Synthesized following the general procedure *o*-bromobenzylbromide **1b** (249 mg, 1.0 mmol), ((prop-2-ynyloxy)methyl)benzene **2f** (292 mg, 2.0 mmol) and CuN_3_ (PCUA) (200 mg, 1 equivalent). ^1^H NMR (400 MHz, CDCl_3_): 4.34 (s, 2H), 4.46 (s, 2H), 4.62 (s, 2H), 4.69 (s, 2H), 5.70 (s, 2H), 6.85 (d, *J*= 7.6 Hz, 1H), 7.14-7.17 (m, 2H), 7.23-7.37 (m, 10H), 7.56 (d, *J*= 8.0 Hz, 1H); ^13^C NMR (100 MHz, CDCl_3_): δ 52.4, 57.5, 62.5, 70.7, 71.7, 72.4, 99.7, 115.0, 120.6, 126.9, 127.7, 127.9, 128.0, 128.3, 128.4 (2C), 128.5 (2C), 128.8 (2C), 129.2, 129.8, 132.9, 133.9, 136.8, 137.7, 147.3.

**1-benzyl-4-((2-bromobenzyloxy)methyl)-5-(3-(2-bromobenzyloxy)prop-1-ynyl)-1H-1,2,3-triazole (3ag):** White solid, (yield: 500 mg, 86%). Synthesized following the general procedure benzyl bromide **1a** (171 mg, 1.0 mmol), 1-bromo-2-((prop-2-ynyloxy)methyl)benzene **2g** (446 mg, 2.0 mmol) and CuN_3_ (PCUA) (200 mg, 1 equivalent). ^1^H NMR (400 MHz, CDCl_3_): 4.47 (s, 2H), 4.62 (s, 2H), 4.65 (s, 2H), 4.74 (s, 2H), 5.55 (s, 2H), 7.08-7.28 (m, 9H), 7.40-7.56 (m, 4H); ^13^C NMR (100 MHz, CDCl_3_): δ 52.9, 58.3, 63.0, 71.3, 71.7, 98.4, 120.0, 122.7, 122.9, 127.3, 127.4, 127.9 (2C), 128.5, 128.8 (3C), 128.9, 129.2, 129.3, 129.4, 132.4, 132.6, 134.3, 136.4, 137.1, 147.1.

**1-(2-bromobenzyl)-4-((2-bromobenzyloxy)methyl)-5-(3-(2-bromobenzyloxy)prop-1-ynyl)-1H-1,2,3-triazole (3bg):** White solid, (yield: 554 mg, 84%). Synthesized following the general procedure *o*-bromobenzylbromide **1b** (249 mg, 1.0 mmol), 1-bromo-2-((prop-2-ynyloxy)methyl)benzene **2g** (446 mg, 2.0 mmol) and CuN_3_ (PCUA) (200 mg, 1 equivalent). ^1^H NMR (400 MHz, CDCl_3_): 4.44 (s, 2H), 4.55 (s, 2H), 4.68 (s, 2H), 4.79 (s, 2H), 5.70 (s, 2H), 6.88 (d, *J*= 6.8 Hz, 1H), 7.07-7.15 (m, 4H), 7.20-7.28 (m, 3H), 7.34-7.36 (m, 1H), 7.47-7.50 (m, 3H); ^13^C NMR (100 MHz, CDCl_3_): δ 48.3, 52.4, 58.3, 60.1, 70.8, 71.2, 71.7, 98.9, 112.8, 117.6, 120.7, 127.3, 127.4, 127.9, 128.8, 128.9, 129.1, 129.2, 129.3, 129.4, 129.8, 132.4, 132.8, 133.8, 16.4, 137.1, 147.1.

**1-benzyl-4-(6-methoxynaphthalen-2-yl)-5-((6-methoxynaphthalen-2-yl)ethynyl)-1H-1,2,3-triazole (3ah):** White solid, (yield: 446 mg, 90%). Synthesized following the general procedure *o*-bromobenzylbromide **1b** (249 mg, 1.0 mmol), 2-ethynyl-6-methoxynaphthalene **2h** (364 mg, 2.0 mmol) and CuN_3_ (PCUA) (200 mg, 1 equivalent). ^1^H NMR (400 MHz, CDCl_3_): 3.92 (s, 3H), 3.94 (s, 3H), 5.71 (s, 2H), 7.13-7.21 (m, 2H), 7.30-7.39 (m, 4H), 7.40-7.50 (m, 3H), 7.73-7.81 (m, 5H), 7.93 (s, 1H), 8.21-8.23 (m, 1H), 8.62 (s, 1H); ^13^C NMR (100 MHz, CDCl_3_): δ 52.5, 53.0, 54.5, 74.3, 103.2, 105.9, 116.3, 117.2, 119.2 (2C), 120.0 (2C), 124.6 (2C), 125.3 (2C), 127.4(2C), 128.2 (3C), 128.9, 129.4 (3C), 129.8 (2C), 131.8, 134.5, 134.9, 148.3, 158.2, 158.9.

**1-(2-bromobenzyl)-4-(6-methoxynaphthalen-2-yl)-5-((6-methoxynaphthalen-2-yl)ethynyl)-1*H*-1,2,3-triazole (3bh):** White solid, (yield: 534 mg, 93%). Synthesized following the general procedure *o*-bromobenzylbromide **1b** (249 mg, 1.0 mmol), 2-ethynyl-6-methoxynaphthalene **2h** (364 mg, 2.0 mmol) and CuN_3_ (PCUA) (200 mg, 1 equivalent). ^1^H NMR (400 MHz, CDCl_3_): 3.91 (s, 3H), 3.93 (s, 3H), 5.85 (s, 2H), 7.02 (d, *J*= 7.2 Hz, 1H), 7.12-7.27 (m, 6H), 7.46 (d, *J*= 8.4 Hz, 1H), 7.62 (d, *J*= 8.0 Hz, 1H), 7.71 (d, *J*= 8.4 Hz, 2H), 7.84-7.93 (m, 3H), 8.34 (d, *J*= 8.4 Hz, 1H), 8.68 (s, 1H); ^13^C NMR (100 MHz, CDCl_3_): δ 52.4, 55.3 (2C), 74.5, 105.7, 105.8, 116.1, 117.9, 119.2, 119.9, 122.6, 124.5, 125.3, 125.6, 127.2, 127.9, 128.1, 128.3, 128.8, 128.9, 129.5 (3C), 129.7, 129.9, 131.8, 132.9, 134.4, 134.6, 134.8, 147.3, 158.2, 158.9.

**4-(6-methoxynaphthalen-2-yl)-5-((6-methoxynaphthalen-2-yl)ethynyl)-1-(4-methylbenzyl)-1H-1,2,3-triazole (3ch):** White solid, (yield: 489 mg, 96%). Synthesized following the general procedure 1-(bromomethyl)-4-methylbenzene **1c** (185 mg, 1.0 mmol), 2-ethynyl-6-methoxynaphthalene **2h** (364 mg, 2.0 mmol) and CuN_3_ (PCUA) (200 mg, 1 equivalent). ^1^H NMR (400 MHz, CDCl_3_): 2.32 (s, 3H), 3.91 (s, 3H), 3.94 (s, 3H), 5.67 (s, 2H), 7.13-7.22 (m, 6H), 7.12-7.27 (m, 6H), 7.35 (d, *J*= 7.6 Hz, 2H), 7.51 (d, *J*= 8.4 Hz, 1H), 7.74-7.82 (m, 4H), 7.96 (s, 1H), 8.29 (d, *J*= 8.8 Hz, 1H), 8.63 (s, 1H); ^13^C NMR (100 MHz, CDCl_3_): δ 21.0, 51.7, 52.8, 55.5, 75.6, 103.2, 106.0, 116.5, 117.6, 119.9, 120.1, 124.6, 125.2, 127.1 (2C), 127.3 (2C), 128.2 (3C), 128.3 (2C), 129.5 (3C), 129.6, 130.2, 131.7, 131.8, 135.0, 138.2, 148.2, 157.9, 159.0.

**1-benzyl-4-butyl-5-(hex-1-ynyl)-1H-1,2,3-triazole (3ai):** White solid, (yield: 257 mg, 87%). Synthesized following the general procedure benzyl bromide **1a** (171 mg, 1.0 mmol), hex-1-yne **2i** (164 mg, 1.0 mmol) and CuN_3_ (PCUA) (200 mg, 1 equivalent). ^1^H NMR (400 MHz, CDCl_3_): 0.90-0.92 (m, 6H), 1.30-1.42 (m, 4H), 1.51-1.55 (m, 2H), 1.65-1.69 (m, 2H), 2.41-2,44 (m, 2H), 2.66-2.69 (m, 2H), 5.46 (s, 2H), 7.25-7.27 (m, 5H); ^13^C NMR (100 MHz, CDCl_3_): δ 13.4, 13.6, 19.2, 21.7, 22.1, 25.1, 30.1, 30.8, 52.3, 66.1, 103.0, 119.1, 127.9 (2C), 128.6, 129.2 (2C), 135.0, 150.2.

**1-(2-bromobenzyl)-4-butyl-5-(hex-1-ynyl)-1H-1,2,3-triazole (3bi):** White solid, (yield: 318 mg, 85%). Synthesized following the general procedure *o*-bromobenzylbromide **1b** (249 mg, 1.0 mmol), hex-1-yne **2i** (164 mg, 1.0 mmol) and CuN_3_ (PCUA) (200 mg, 1 equivalent). ^1^H NMR (400 MHz, CDCl_3_): 0.85 (t, *J*= 7.2 Hz, 3H), 0.93 (t, *J*= 7.4 Hz, 3H), 1.28-1.39 (m, 4H), 1.43-1.48 (m, 2H), 1.66-1.72 (m, 2H), 2.38 (t, *J*= 7.0 Hz, 2H), 2.72 (t, *J*= 7.6 Hz, 2H), 5.60 (s, 2H), 6.72-6.74 (m, 1H), 7.11-7.14 (m, 1H), 7.18-7.22 (m, 1H), 7.54-7.56 (m, 1H); ^13^C NMR (100 MHz, CDCl_3_): δ 13.5, 13.8, 19.3, 21.8, 22.3, 25.3, 30.1, 31.0, 52.0, 65.8, 103.7, 119.9, 122.4, 127.8, 128.5, 129.4, 132.7, 134.7, 150.2.

**4-butyl-5-(hex-1-ynyl)-1-(4-methylbenzyl)-1H-1,2,3-triazole (3ci):** White solid, (yield: 281 mg, 91%). Synthesized following the general procedure 1-(bromomethyl)-4-methylbenzene **1c** (185 mg, 1.0 mmol), hex-1-yne **2i** (164 mg, 1.0 mmol) and CuN_3_ (PCUA) (200 mg, 1 equivalent). ^1^H NMR (400 MHz, CDCl_3_): 0.85-0.91 (m, 6H), 1.31-1.44 (m, 4H), 1.62-1.68 (m, 2H), 2.30 (s, 3H), 2.44 (t, *J*= 6.8 Hz, 2H), 2.67 (t, *J*= 7.8 Hz, 2H), 5.42 (s, 2H), 7.10-7.13 (m, 2H), 7.16-7.18 (m, 1H); ^13^C NMR (100 MHz, CDCl_3_): δ 13.5, 13.7, 19.3, 21.9, 22.2, 25.2, 30.2, 30.9, 52.3, 66.3, 102.9, 115.0, 118.5, 119.1, 127.8, 129.2, 129.3, 132.2, 137.9, 150.3.

**1-benzyl-4-(4-nitrophenyl)-1H-1,2,3-triazole** **(4aj):**^2^ White solid, (yield: 232 mg, 83%). Light yellow solid, (266 mg, mp: 136-138^o^C. Synthesized following the general procedure from benzyl bromide **1a** (171 mg, 1.0 mmol), 1-ethynyl-4-nitrobenzene **2j** (294 mg, 2.0 mmol) and CuN_3_ (PCUA) (200 mg, 1 equivalent). ^1^H NMR (400 MHz, CDCl_3_): δ 5.58 (s, 2H), 7.30-7.38 (m, 5H), 7.81 (s, 1H), 7.93 (d, *J*= 8.8 Hz, 2H), 8.21 (d, *J*= 8.8 Hz, 2H); ^13^C NMR (100 MHz, CDCl_3_): δ 54.4, 121.0, 124.1, 124.2, 126.0, 126.1, 128.0, 128.1, 129.0, 129.1, 129.2, 134.1, 136.8, 145.9, 147.2.

**1-(2-bromobenzyl)-4-(4-nitrophenyl)-1H-1,2,3-triazole** **(4bj):** Light yellow solid, (yield: 312 mg, 87%). Synthesized following the general procedure from *o*-bromobenzylbromide **1b** (249 mg, 1.0 mmol), 1-ethynyl-4-nitrobenzene **2j** (294 mg, 2.0 mmol) and CuN_3_ (PCUA) (200 mg, 1 equivalent). ^1^H NMR (400 MHz, CDCl_3_): δ 5.73 (s, 2H), 7.27 (d, *J*= 7.6 Hz, 2H), 7.32-7.35 (m, 1H), 7.64 (d, *J*= 7.6 Hz, 1H), 7.93 (s, 1H), 7.98 (d, *J*= 8.4 Hz, 2H), 8.25 (d, *J*= 8.4 Hz, 2H); ^13^C NMR (100 MHz, CDCl_3_): δ 54.4, 121.3, 123.6, 124.2 (2C), 126.1 (2C), 128.4, 130.6, 130.7, 133.4, 133.7, 136.7, 145.8, 147.3.

**1-(1-benzyl-1*H*-1,2,3-triazol-4-yl)cyclohexanol** ( TA-9c, **4ak**):^3^ White solid, (yield: 206 mg, 80%). Synthesized following the general procedure from *o*-bromobenzylbromide **1b** (249 mg, 1.0 mmol), 1-ethynylcyclohexanol **2k** (248 mg, 2.0 mmol), and CuN_3_ (PCUA) (200 mg, 1 equivalent). ^1^H NMR (400 MHz, CDCl_3_): δ 1.26-1.88 (m, 10H), 2.99 (s, 1H), 5.42 (s, 2H), 7.18-7.21 (m, 2H), 7.29-7.31 (m, 3H), 7.34 (s, 1H); ^13^C NMR (100 MHz, CDCl_3_): δ 21.6 (2C), 25.0, 37.7 (2C), 53.7, 69.1, 119.4, 127.8 (2C), 128.3, 128.7 (2C), 134.4, 155.8.

**1-(1-(2-bromobenzyl)-1*H*-1,2,3-triazol-4-yl)cyclohexanol** (**4bk**):^3^ White solid, (yield: 286 mg, 85%). Synthesized following the general procedure from *o*- *o*-bromobenzylbromide **1b** (249 mg, 1.0 mmol), 1-ethynylcyclohexanol **2k** (248 mg, 2.0 mmol) and CuN_3_ (PCUA) (200 mg, 1 equivalent).  ^1^H NMR (400 MHz, CDCl_3_): δ 1.34-1.97 (m, 10H), 2.73 (s, 1H), 5.59 (s, 2H), 7.07 (dd, *J*=7.6, 1.6 Hz, 1H), 7.16 (dt, *J*=7.7, 1.7 Hz, 1H), 7.25 (dt, *J*=7.6, 1.2 Hz, 1H) 7.45 (s, 1H) 7.56 (dd, *J*=8.0, 1.2 Hz, 1H); ^13^C NMR (100 MHz, CDCl_3_): δ 21.8 (2C), 25.2, 38.0 (2C), 53.6, 69.5, 119.9, 123.3, 128.1, 130.1, 130.2, 133.1, 134.2, 155.9.

**(1-Benzyl-1*H*-1,2,3-triazol-4-yl)methanol** (**5al**):^4^ White solid, (yield: 170 mg, 90%). Synthesized following the general procedure from benzyl bromide **1a** (171 mg, 1.0 mmol), NaN_3_(65 mg, 1.0 mmol), prop-2-yn-1-ol **2l** (56 mg, 1.0 mmol) ^1^H NMR (400 MHz, CDCl_3_): δ 4.43 (brs, O*H*, 1H), 4.63 (s, 2H), 5.37 (s, 2H), 7.15-7.17 (m, 2H), 7.23-7.26 (m, 3H), 7.43 (s, 1H); ^13^C NMR (100 MHz, CDCl_3_): δ 53.8, 55.6, 121.9, 127.8 (2C), 128.4, 128.8 (2C), 134.4, 148.1.

**(1-Benzyl-1*H*-1,2,3-triazol-4-yl)methanol** (**5bl**):^4^ White solid, (yield: 247 mg, 92%). Synthesized following the general procedure from *o*-bromobenzylbromide **1b** (249 mg, 1.0 mmol),n NaN_3_(65 mg, 1.0 mmol), prop-2-yn-1-ol **2l** (56 mg, 1.0 mmol) ^1^H NMR (400 MHz, CDCl_3_): δ 3.41 (brs, O*H*, 1H), 4.72 (s, 2H), 5.60 (s, 2H), 7.10-7.13 (m, 1H), 7.16-7.28 (m, 2H), 7.54 (s, 1H), 7.57 (d, *J*= 8.0 Hz, 1H); ^13^C NMR (100 MHz, CDCl_3_): δ 53.7, 56.2, 121.9, 123.4, 128.1, 130.2, 130.3, 133.1, 133.9, 148.0.

**4-(4-methoxy-2-methyl-phenyl)-1-(2,3,4,6-tetra-*O*-acetyl-β-D--glucopyranosyl)-1*H*-1,2,3-triazole** (**6ad**):^2^ White solid, (yield: 468 mg, 90%). Synthesized following the general procedure from 1-azido-2,3,4,6-tetra-*O*-acetyl-β-D-glucopyranose **5a** (373 mg, 1.0 mmol), 1-ethynyl-4-methoxy-2-methylbenzene **2d** (146 mg, 1.0 mmol). ^1^H NMR (400 MHz, CDCl_3_): δ1.97 (s, 6H), 2.01 (s, 6H), 2.36 (s, 3H), 3.76 (s, 3H), 3.99-4.13 (m, 2H), 4.26 (dd, *J*= 12.4, 4.8 Hz, 1H), 5.23 (t, *J*= 1,6 Hz, 1H), 5.40 (t, *J*= 9.4 Hz, 1H), 5.47-5.52 (m, 1H) 5.91 (d, *J*= 9.2, 1H), 6.75 (s, 2H), 7.60-7.62 (m, 1H), 7.80 (s, 1H). ^13^C NMR (100 MHz, CDCl_3_): δ 20.1, 20.5, 20.6, 21.0, 21.4, 55.2, 61.6, 67.8, 70.3, 72.7, 75.0, 85.7, 114.4, 116.2, 119.4, 122.0, 130.3, 137.3, 147.5, 159.6, 168.9, 169.4, 169.8, 170.4.

**4-(4-methoxy-2-methyl-phenyl)-1-(2,3,4,6-tetra-*O*-acetyl-β-D-galactopyranosyl)-1*H*-1,2,3-triazole** (**6bd**):^2^ White solid, (yield: 458 mg, 88%). Synthesized following the general procedure from 1-azido-2,3,4,6-tetra-*O*-acetyl-β-D-galactopyranose **5b** (373 mg, 1.0 mmol), 1-ethynyl-4-methoxy-2-methylbenzene **2d** (146 mg, 1.0 mmol). ^1^H NMR (400 MHz, CDCl_3_): δ1.85 (s, 3H), 1.97 (s, 3H), 1.99 (s, 3H), 2.18 (s, 3H) 2.40 (s, 3H), 3.78 (s, 3H), 4.13-4.17 (m, 2H), 4.23-4.26 (m, 1H), 5.25 (dd, *J*= 10.4, 3.2 Hz, 1H), 5.53 (d, *J*= 2.8 Hz, 1H), 5.60 (t, *J*= 10.0 Hz, 1H), 5.88 (d, *J*= 9.2 Hz, 1H) 6.77 (s, 2H), 7.61 (d, *J*= 9.2 Hz, 1 H) 7.81 (s, 1H); ^13^C NMR (100 MHz, CDCl_3_): δ 20.1 (2C), 20.4, 20.5, 21.3, 55.1, 61.1, 66.9, 67.8, 70.7, 73.9, 86.2, 111.3, 116.1, 119.4, 122.0, 130.3, 137.3, 147.5, 159.5, 169.0, 169.7, 169.9, 170.2.

**4-(4-fluoro-3-methylphenyl)-1-(2,3,4,6-tetra-*O*-acetyl-β-D--glucopyranosyl)-1*H*-1,2,3-triazole** (**6ae**): White solid, (yield: 406 mg, 80%). Synthesized following the general procedure from 1-azido-2,3,4,6-tetra-*O*-acetyl-β-D-glucopyranose **5a** (373 mg, 1.0 mmol), 4-ethynyl-1-fluoro-2-methylbenzene 2**e** (134 mg, 1.0 mmol) and CuN_3_ (PCUA) (200 mg, 1 equivalent). ^1^H NMR (400 MHz, CDCl_3_): δ 1.82 (s, 3H), 1.99 (s, 3H), 2.02 (s, 6H), 2.25 (s, 3H), 3.99-4.13 (m, 2H), 4.28 (dd, *J*= 12.4, 5.0 Hz, 1H) 5.23 (t, J= 9.6 Hz, 1H), 5.39-5.50 (m, 2H), 5.91 (d, J= 9.2 Hz, 1H), 6.99 (t, J= 8.8 Hz, 1H), 7.52-7.56 (m,1H), 7.63 (d, J= 6.0 Hz, 1H), 7.95 (s, 1H); ^13^C NMR (100 MHz, CDCl_3_): δ 14.4, 20.0 (2C), 20.4, 20.5, 61.5, 67.6, 70.2, 72.6, 75.0, 85.7, 115.5 (d, J= 22.0 Hz), 117.4, 124.85 (d, *J*_CF_= 8.1 Hz, 1C), 125.3 (d, *J*_CF_= 17.6 Hz, 1C) 125.7 (d, *J*_CF_= 3.5 Hz, 1C), 129.0 (d, *J*_CF_= 5.0 Hz, 1C), 147.7, 163.8 (d, *J*_CF_= 245 Hz, 1C), 168.9, 169.3, 169.8, 170.4; ^19^F NMR (400 MHz, CDCl_3_): δ -117.2.

**4-(4-fluoro-3-methylphenyl)-1-(2,3,4,6-tetra-*O*-acetyl-β-D-galactopyranosyl)-1*H*-1,2,3-triazole** (**6be**): White solid, (yield: 416 mg, 82%). Synthesized following the general procedure from 1-azido-2,3,4,6-tetra-*O*-acetyl-β-D-galactopyranose **1b** (373 mg, 1.0 mmol), 4-ethynyl-1-fluoro-2-methylbenzene 2**e** (134 mg, 1.0 mmol) and CuN_3_ (PCUA) (200 mg, 1 equivalent). ^1^H NMR (400 MHz, CDCl_3_): δ1.85 (s, 3H), 1.97 (s, 3H), 1.99 (s, 6H), 2.27 (s, 3H), 4.13-4.16 (m, 2H), 4.23-4.25 (m, 1H), 5.26 (dd, J= 10.4, 3.2 2H), 5.52-5.59 (m, 2H), 5.87 (d, J= 9.2 Hz, 1H), 7.0 (t, J= 9.0 Hz, 1H), 7.55-7.59 (m,1H), 7.64 (d, J= 1.2 Hz, 1H), 7.66 (s, 1H); ^13^C NMR (100 MHz, CDCl_3_): δ 14.4 (d, J= 3.0 Hz), 20.1, 20.4, 20.5, 20.5, 61.1, 66.9, 67.8, 70.7, 73.9, 86.2, 115.3 (d, *J*_CF_= 23.0 Hz, 1C), 117.4, 124.8 (d, *J*_CF_= 8.2 Hz, 1C), 125.3 (d, *J*_CF_= 17.6 Hz, 1C) 125.7 (d, *J*_CF_= 5.2 Hz, 1C), 147.7, 163.8 (d, *J*_CF_= 245 Hz, 1C), 168.9, 169.3, 169.8, 170.4; ^19^F NMR (400 MHz, CDCl_3_): δ -117.4.

**4-(4-nitro-phenyl)-1-(2,3,4,6-tetra-*O*-acetyl-β-D--glucopyranosyl)-1*H*-1,2,3-triazole** (**6aj**):^6^ White solid, (yield: 390 mg, 75%) Synthesized following the general procedure from 1-azido-2,3,4,6-tetra-*O*-acetyl-β-D-galactopyranose **5a** (373 mg, 1.0 mmol), 1-ethynyl-4-nitrobenzene **2j** (147 mg, 1.0 mmol). ^1^H NMR (400 MHz, CDCl_3_): δ 1.88 (s, 3H), 2.03 (s, 3H), 2.06 (s, 3H), 2.07 (s, 3H), 4.01-4.05 (m, 1H), 4.12-4.16 (m, 1H), 4.33 (dd, *J*= 12.6, 5.0 Hz, 1H), 5.26 (t, *J*= 9.6 Hz, 1H), 5.41-5.48 (m, 2H), 5.93 (d, *J*= 8.8 Hz, 1H), 8.00 (d, *J*= 8.8 Hz, 2H), 8.15 (s, 1H), 8.29 (d, *J*= 8.8 Hz, 2H ); ^13^C NMR (100 MHz, CDCl_3_): δ 20.1 (2C), 20.4, 20.6, 61.5, 67.6, 70.3, 72.4, 75.2, 85.9, 119.4, 124.2 (2C), 126.4 (2C), 136.1, 146.2, 147.5, 169.0, 169.3, 169.7, 170.4.

**4-(4-nitro-phenyl)-1-(2,3,4,6-tetra-*O*-acetyl-β-D- galactopyranosyl)1*H*-1,2,3-triazole** (**6bj**): White solid, (yield: 380 mg, 73%). Synthesized following the general procedure from 1-azido-2,3,4,6-tetra-*O*-acetyl-β-D-galactopyranose **5b** (373 mg, 1.0 mmol), 1-ethynyl-4-nitrobenzene **2j** (147 mg, 1.0 mmol). ^1^H NMR (400 MHz, CDCl_3_): δ1.87 (s, 3H), 1.98 (s, 3H), 1.99 (s, 3H), 2.20 (s, 3H), 4.05-4.07 (m, 1H), 4.15-4.17 (m, 1H), 4.26-4.27 (m, 1H), 5.28 (dd, *J*= 10.4, 3.2 Hz, 1H), 5.53-5.58 (m, 2H), 5.90 (d, *J*= 9.2 Hz, 1H), 7.99 (d, *J*= 8.8 Hz, 2H), 8.18 (s, 1H), 8.25 (d, *J*= 8.8 Hz, 2H); ^13^C NMR (100 MHz, CDCl_3_): δ 20.2, 20.4 (2C), 20.5, 61.1, 66.8, 67.9, 70.6, 74.2, 86.3, 119.5, 124.2 (2C), 126.4 (2C), 136.1, 146.1, 147.5, 169.2, 169.7, 169.8, 170.2.

**References:**

1. L. Li, X. Fan, Y. Zhang, A. Zhu, G. Zhang, Tetrahedron, **2013**, 69, 9939-9946.

2. D. Nandi, A. Taher, R. U. Islam, S. Siwal, M. Choudhary, K. Mallick, R. Soc. Open Sci. **2016** 3:11, 160580

3. D. Nandi, A. Taher, R. U. Islam, M. Choudhary, S. Siwal, K. Mallick Sci. Rep.,  **2016**, 6:33025.

4. R. Islam, A. Taher, M. Choudhary, S. Siwal, K. Mallick, Sci. Rep., **2015**, 5. 9632.

6. R. Islam, A. Taher, M. Choudhary, J. Witcomb, K.  Mallick, 
Dalton Trans., **2014**, 44, 1341-1349.

7. D. [Goyard,](http://0-fl-www.reaxys.com.ujlink.uj.ac.za/reaxys/secured/paging.do?performed=true&action=get_preparations&databaseId=0&rnd=0.5584709553002796) A. [Chajistamatiou,](http://0-fl-www.reaxys.com.ujlink.uj.ac.za/reaxys/secured/paging.do?performed=true&action=get_preparations&databaseId=0&rnd=0.5584709553002796) A. [Sotiropoulou,](http://0-fl-www.reaxys.com.ujlink.uj.ac.za/reaxys/secured/paging.do?performed=true&action=get_preparations&databaseId=0&rnd=0.5584709553002796) E. [Chrysina,](http://0-fl-www.reaxys.com.ujlink.uj.ac.za/reaxys/secured/paging.do?performed=true&action=get_preparations&databaseId=0&rnd=0.5584709553002796) J. [Praly,](http://0-fl-www.reaxys.com.ujlink.uj.ac.za/reaxys/secured/paging.do?performed=true&action=get_preparations&databaseId=0&rnd=0.5584709553002796) S. Vidal, Chem. Eur. J., **2014**, 20, 5423-5432.


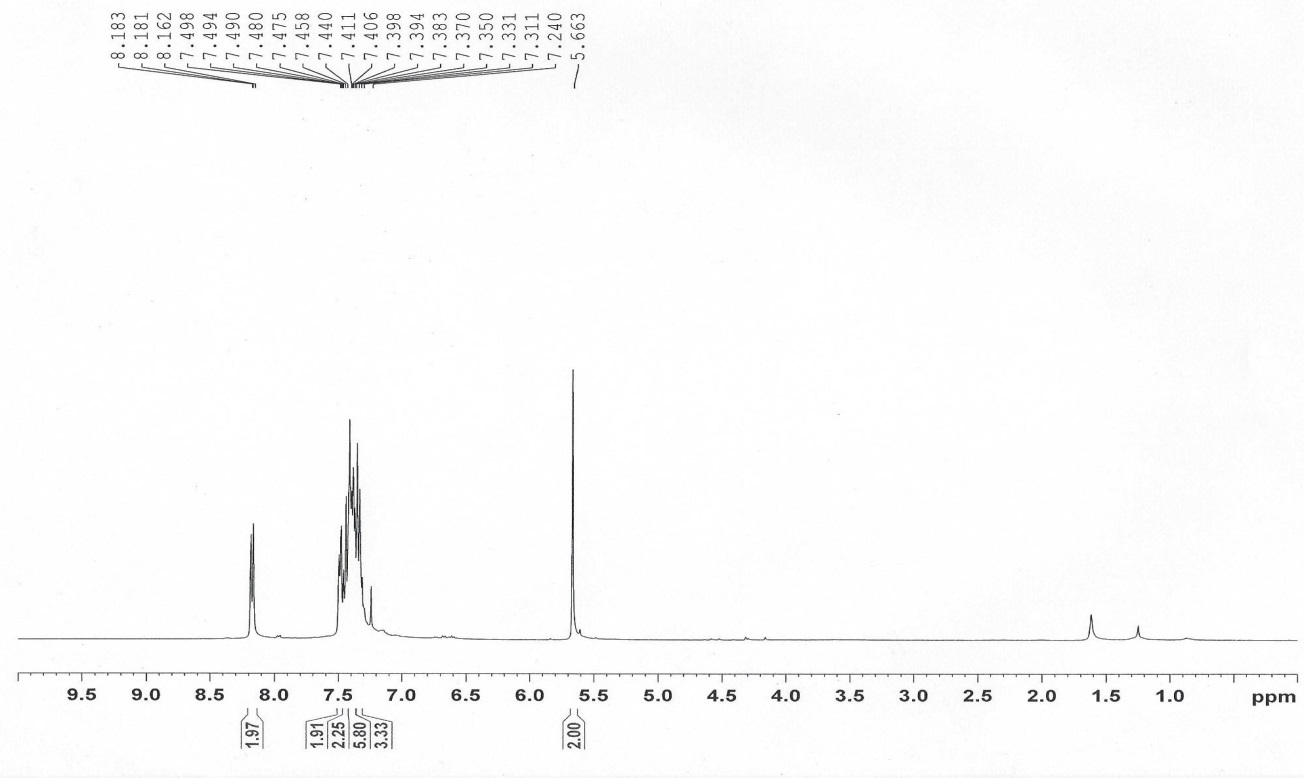


**Figure S1**. ^1^H NMR spectrum of **3aa** in CDCl_3_


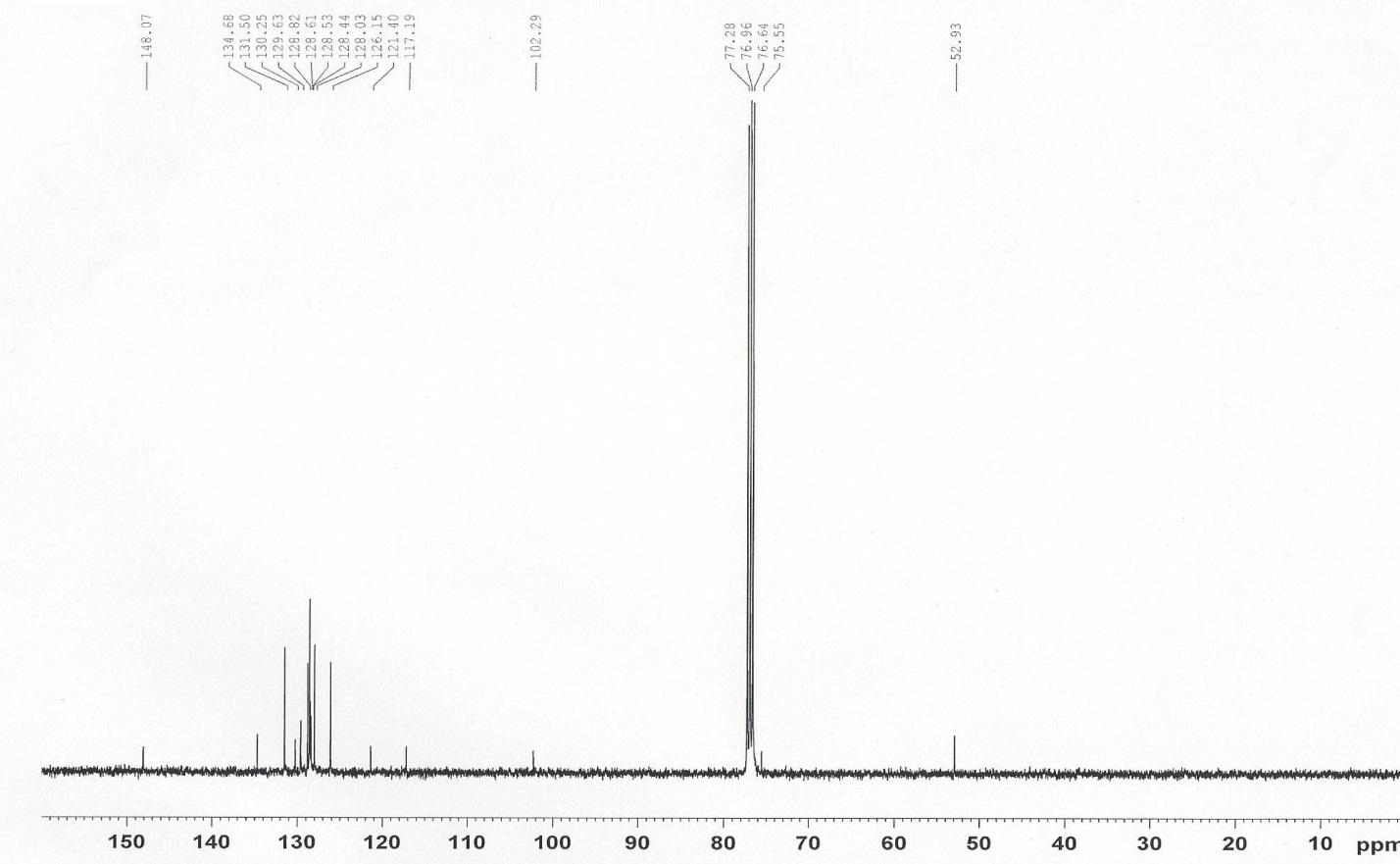


**Figure S2**. ^13^C NMR spectrum of **3aa** in CDCl_3_


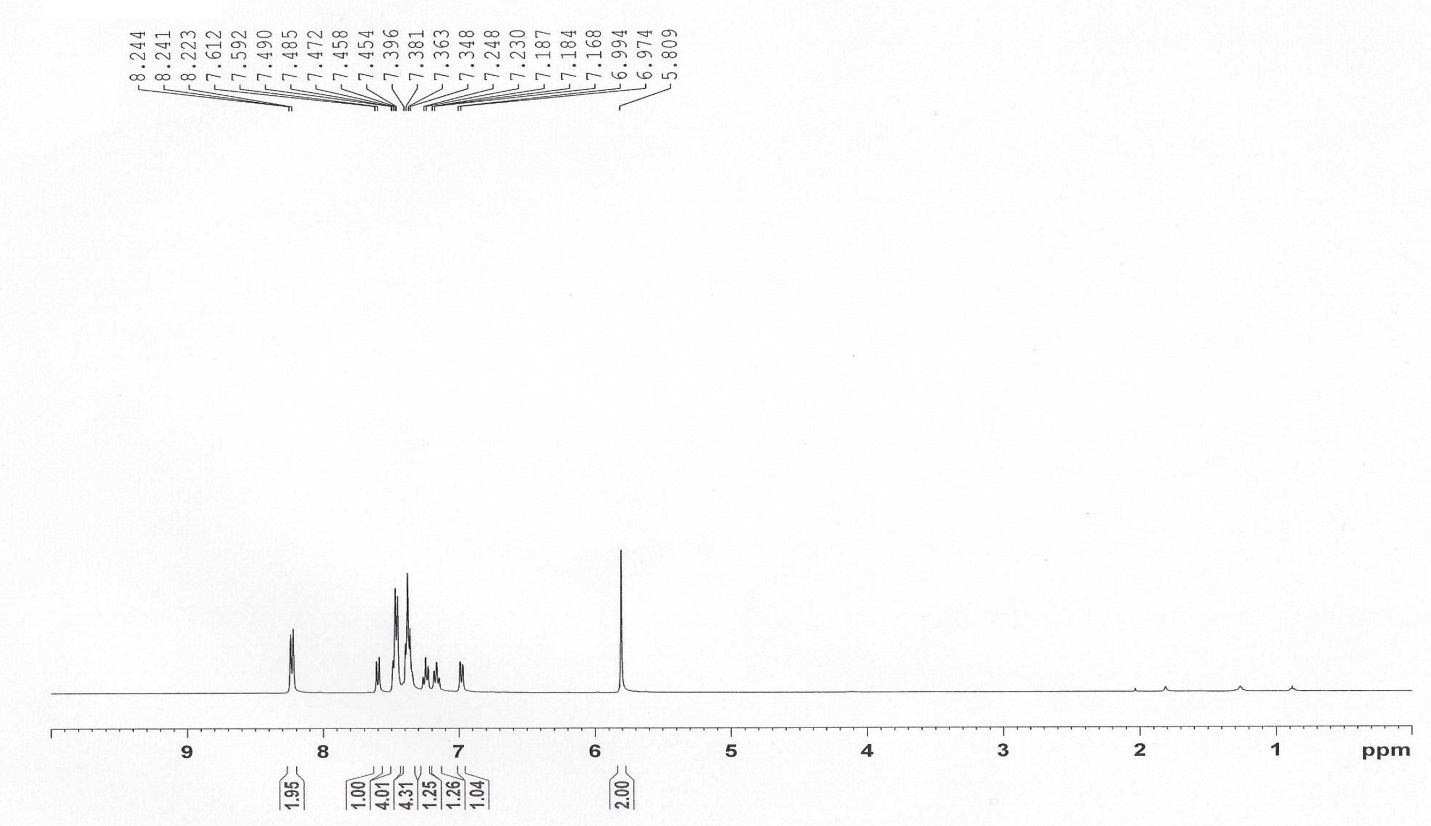


**Figure S3**. ^1^H NMR spectrum of **3ba** in CDCl_3_


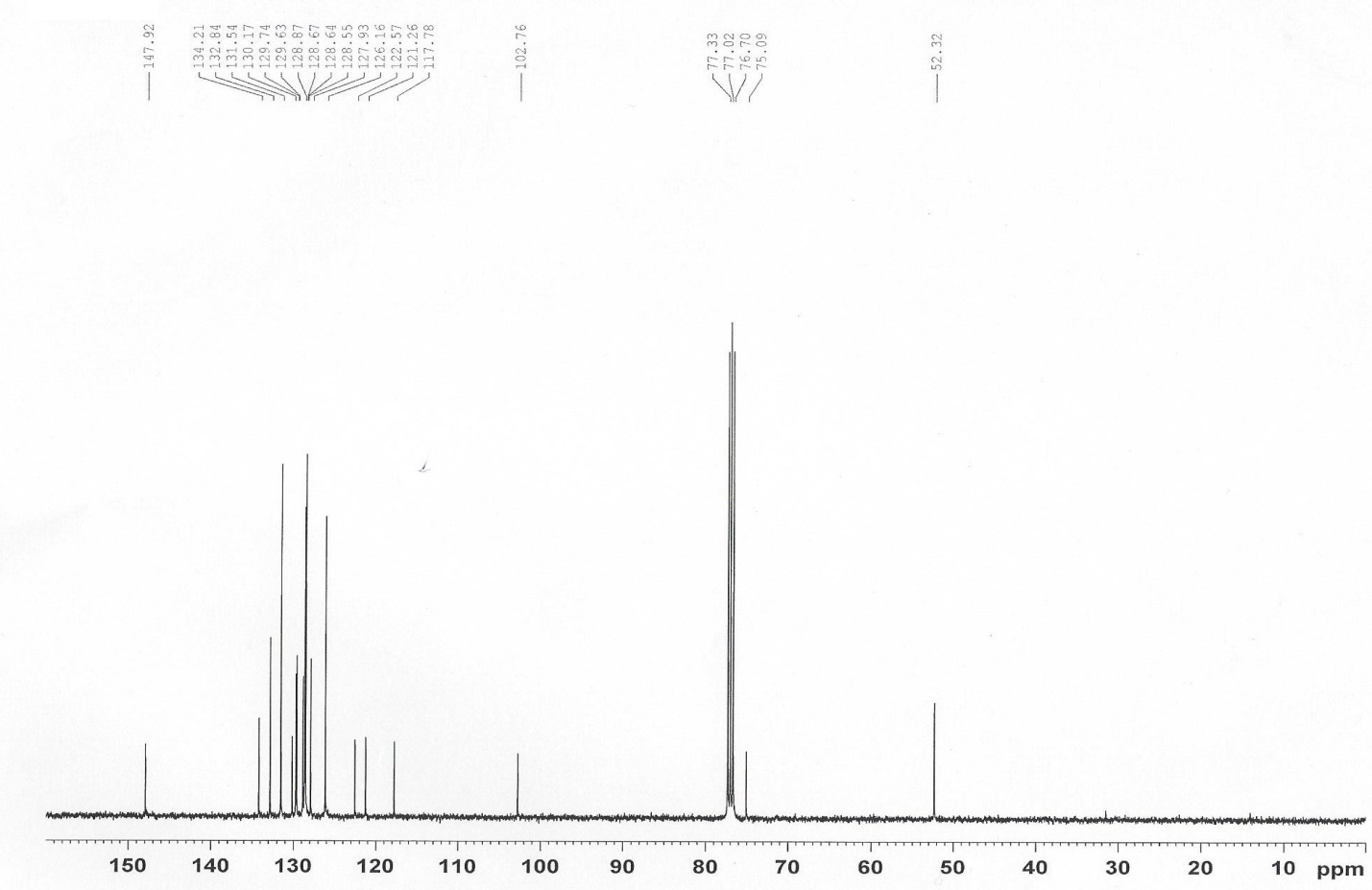


**Figure S4**. ^13^C NMR spectrum of **3ba** in CDCl_3_


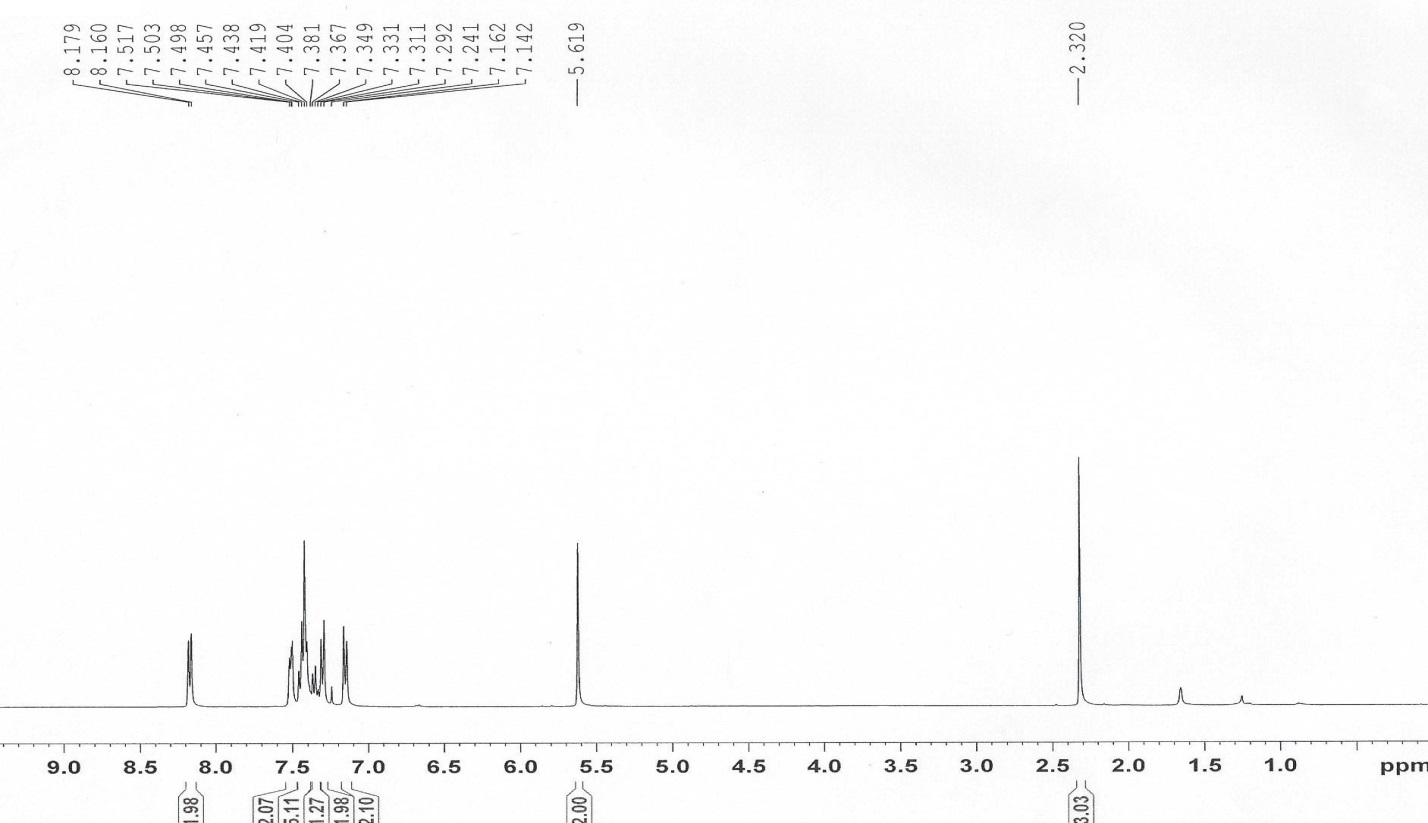


**Figure S5**. ^1^H NMR spectrum of **3ca** in CDCl_3_


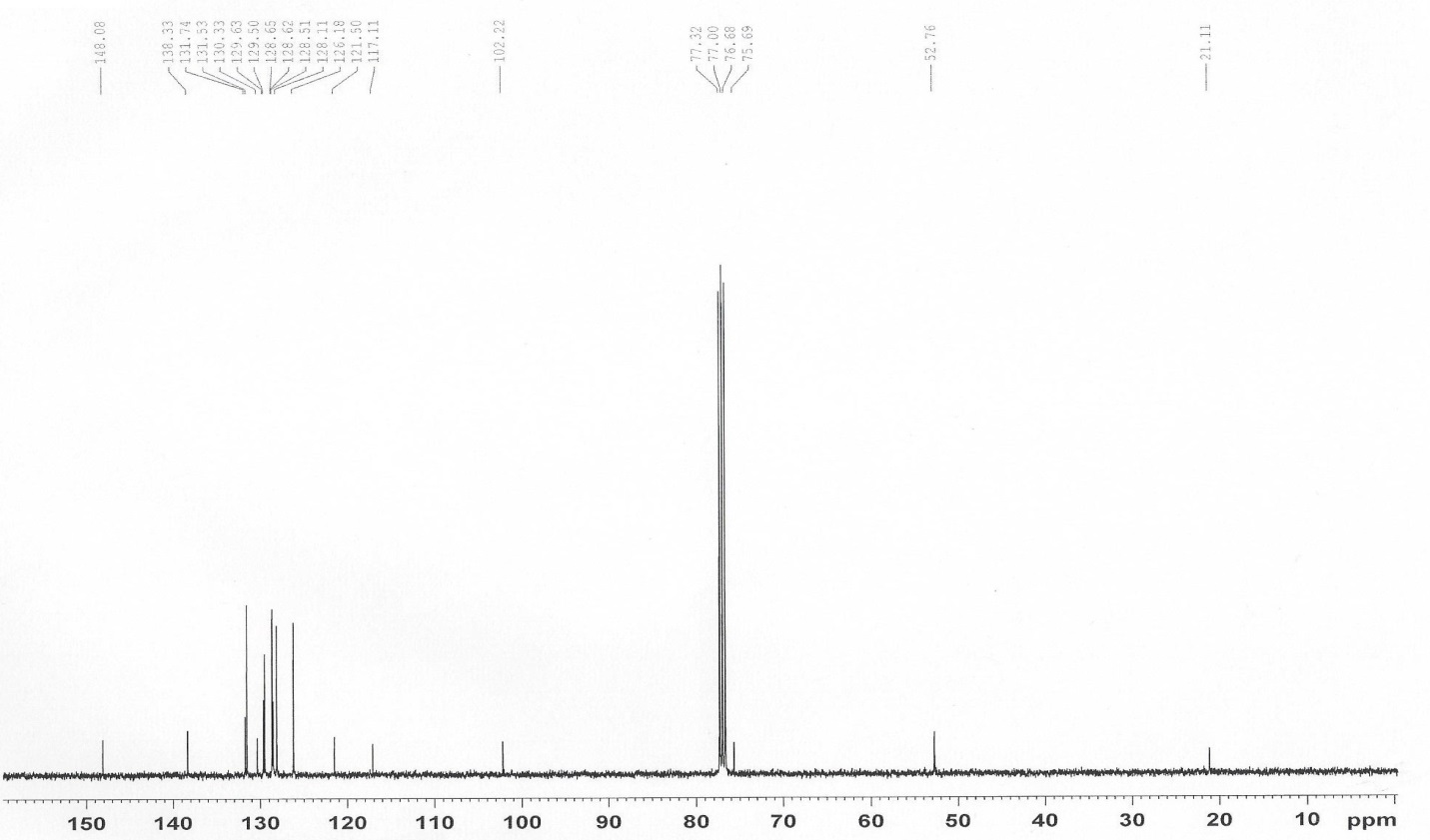


**Figure S6**. ^13^C NMR spectrum of **3ca** in CDCl_3_


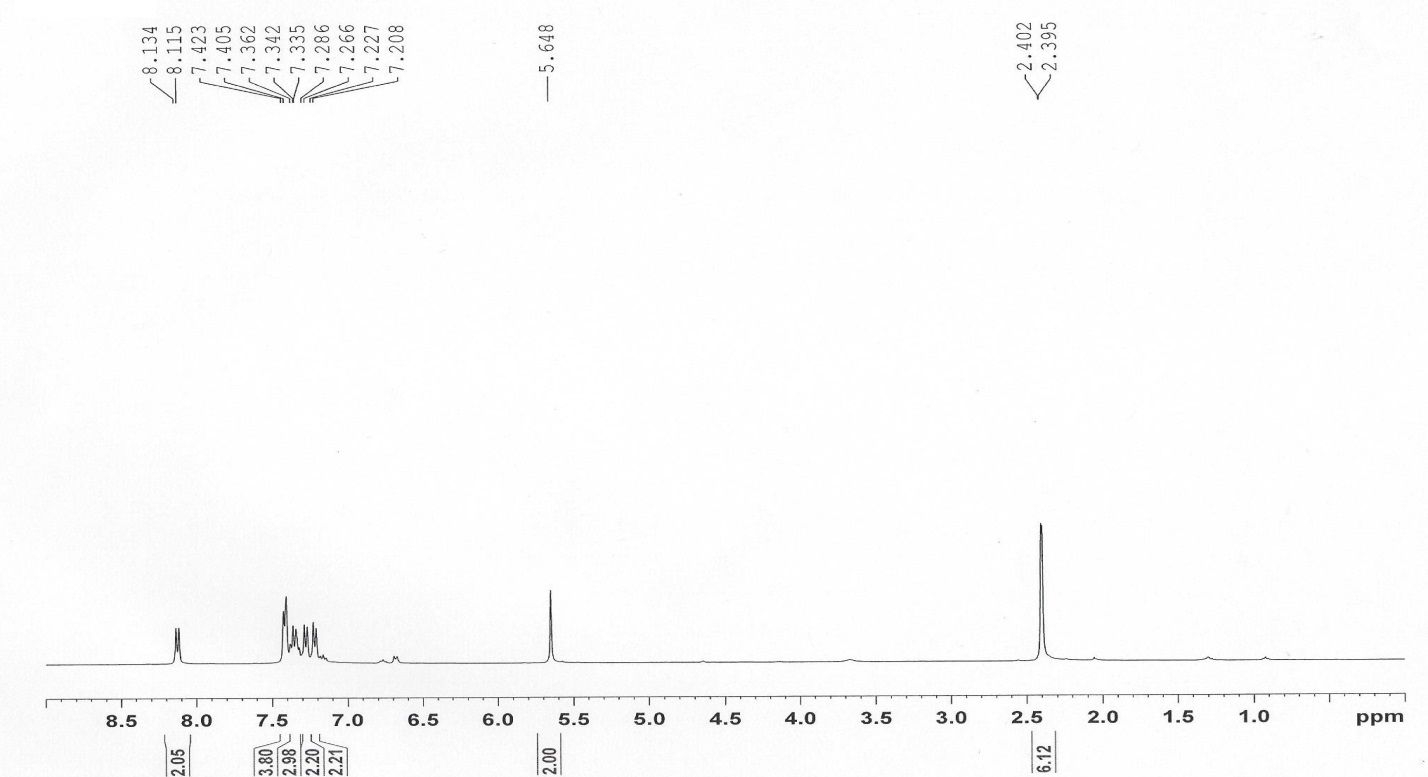


**Figure S7**. ^1^H NMR spectrum of **3ab** in CDCl_3_


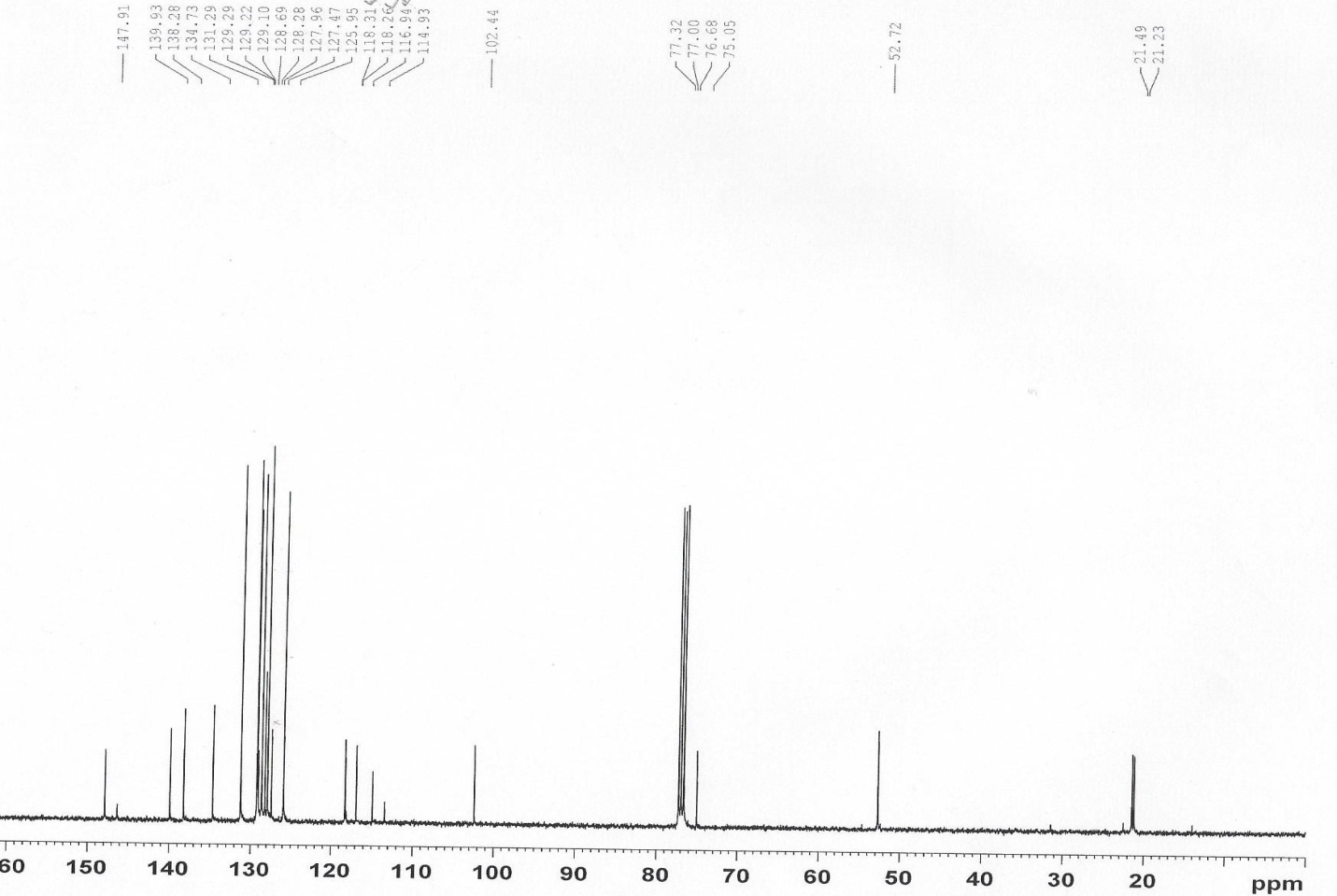


**Figure S8**. ^13^C NMR spectrum of **3ab** in CDCl_3_


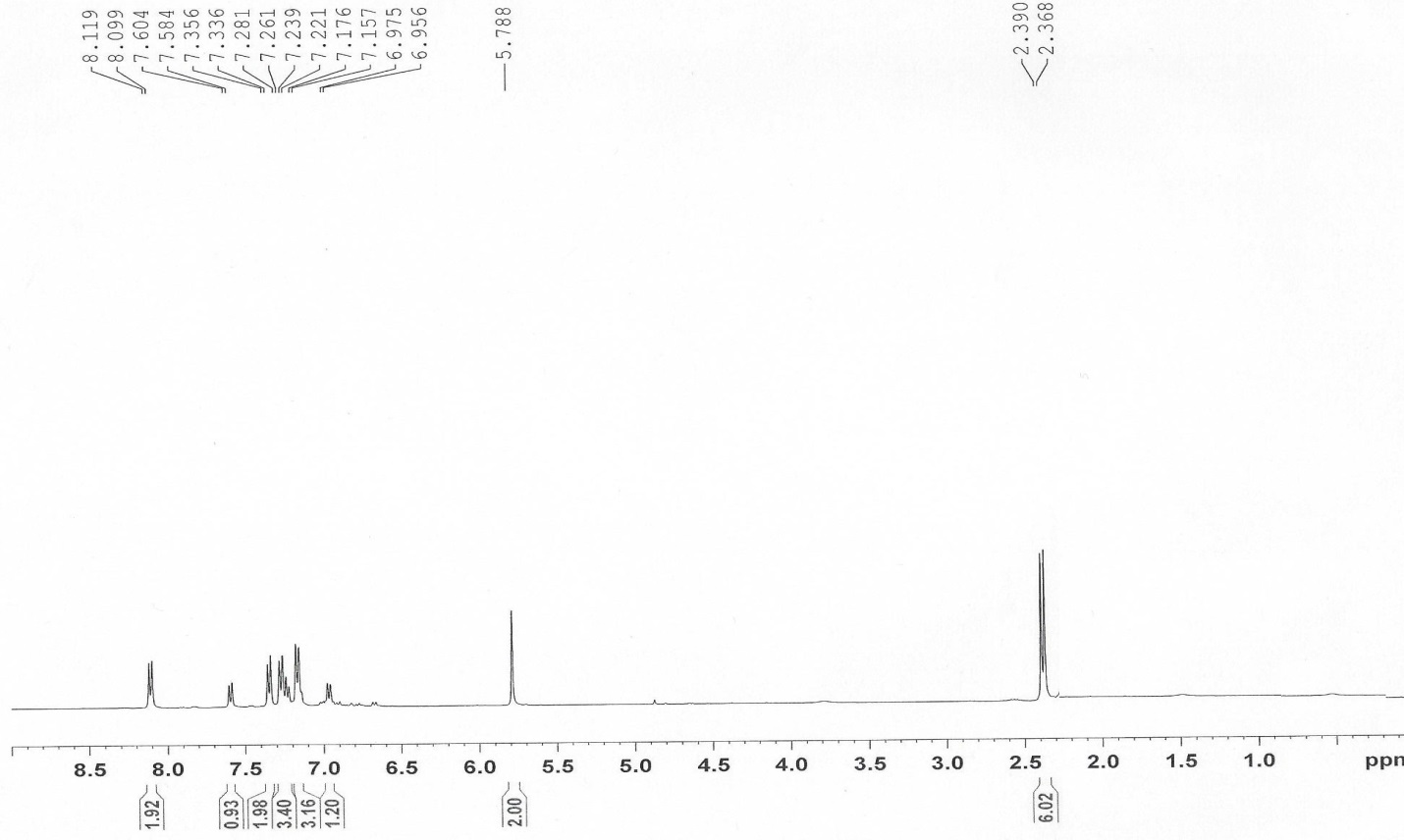


**Figure S9**. ^1^H NMR spectrum of **3bb** in CDCl_3_


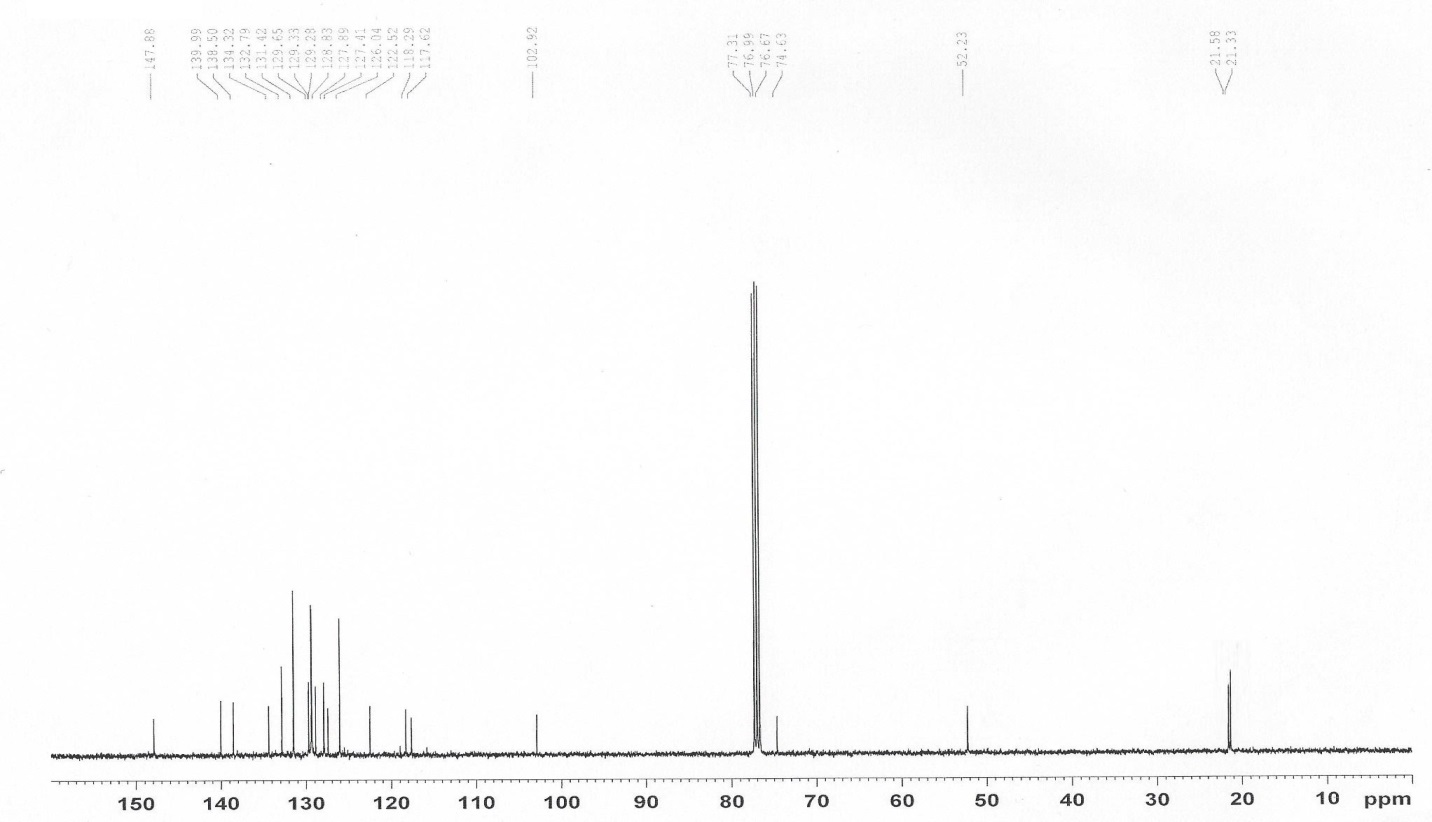


**Figure S10**. ^13^C NMR spectrum of **3bb** in CDCl_3_


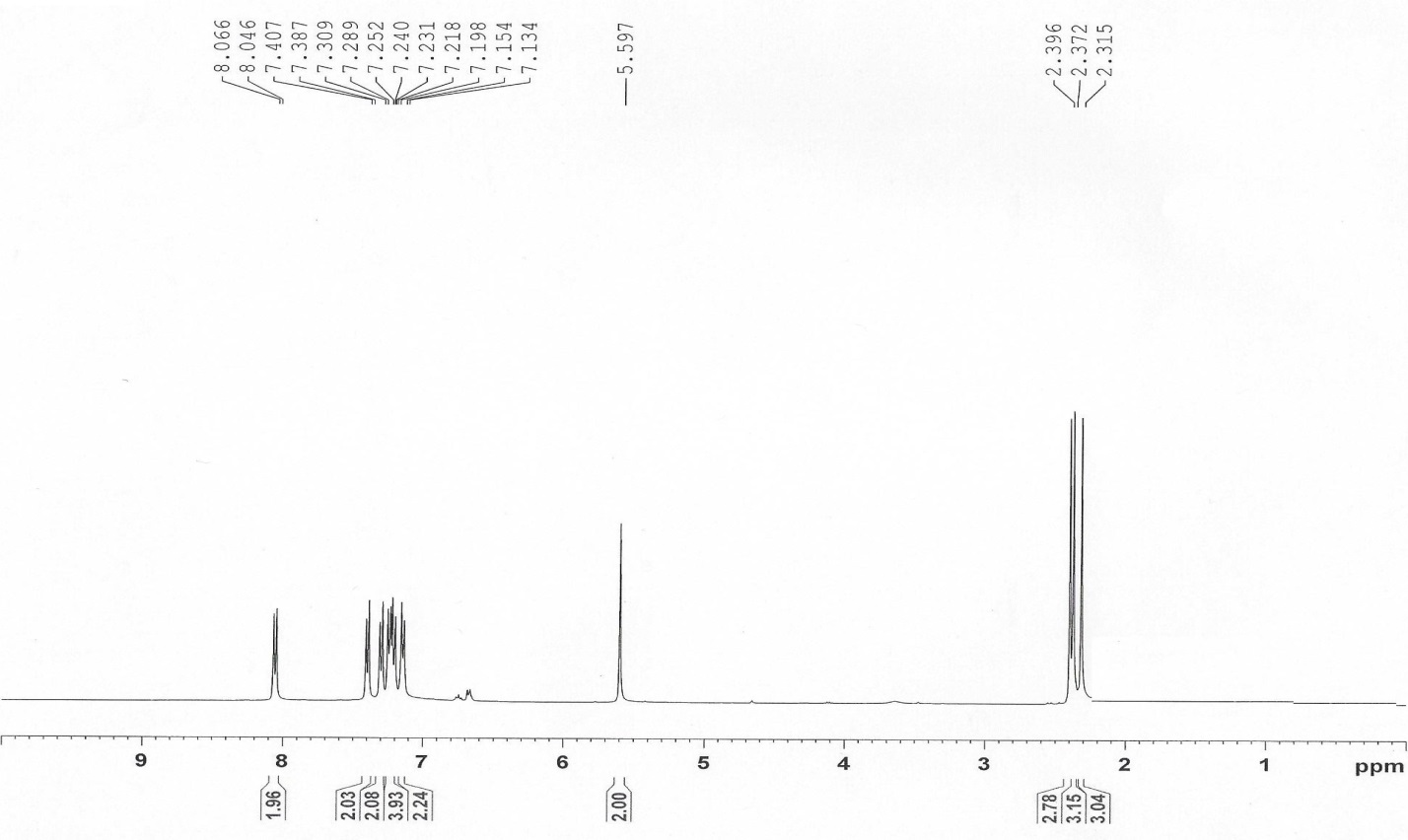


**Figure S11**. ^1^H NMR spectrum of **3cb** in CDCl_3_


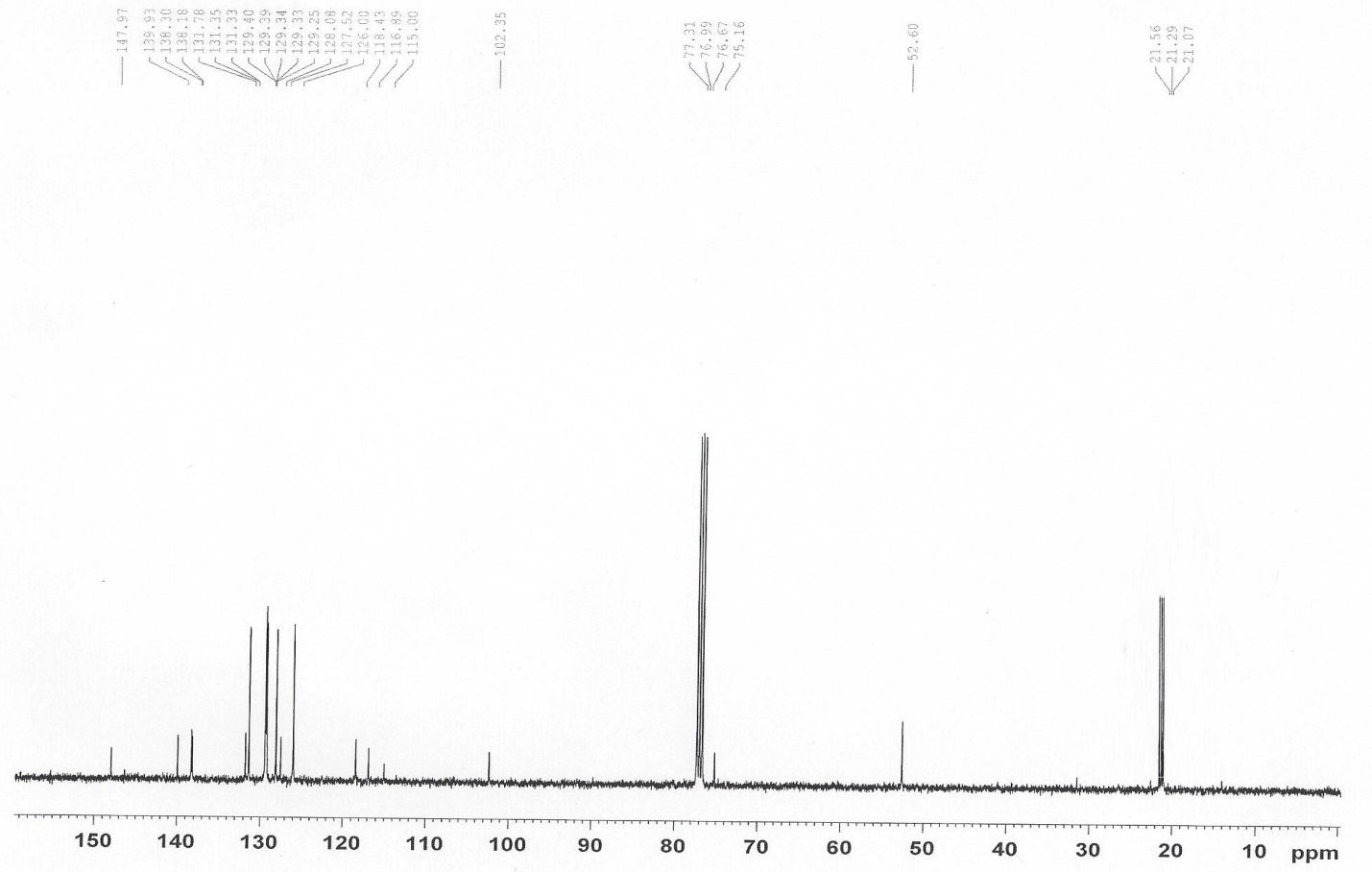


**Figure S12**. ^13^C NMR spectrum of **3cb** in CDCl_3_


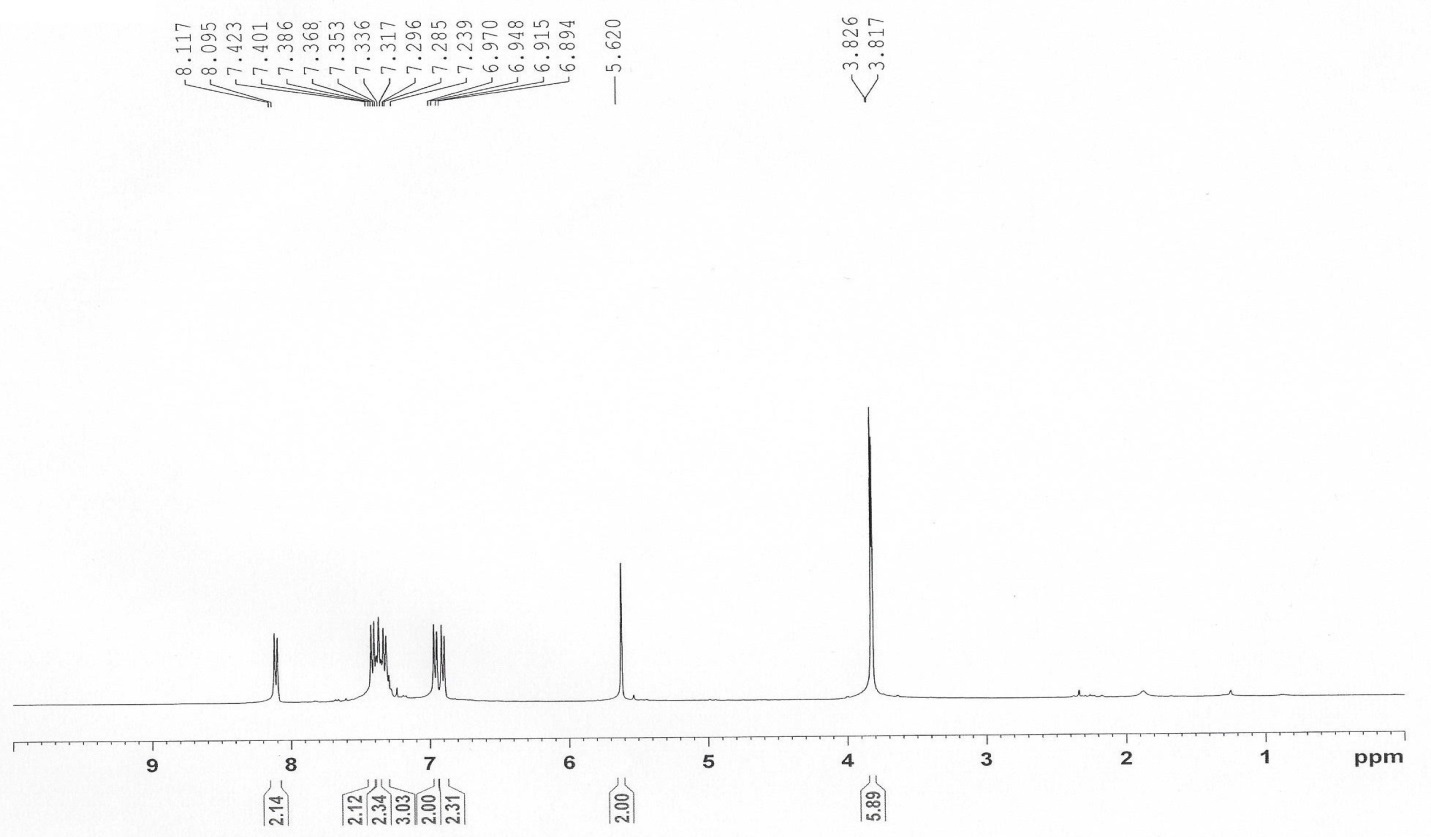


**Figure S13**. ^1^H NMR spectrum of **3ac** in CDCl_3_


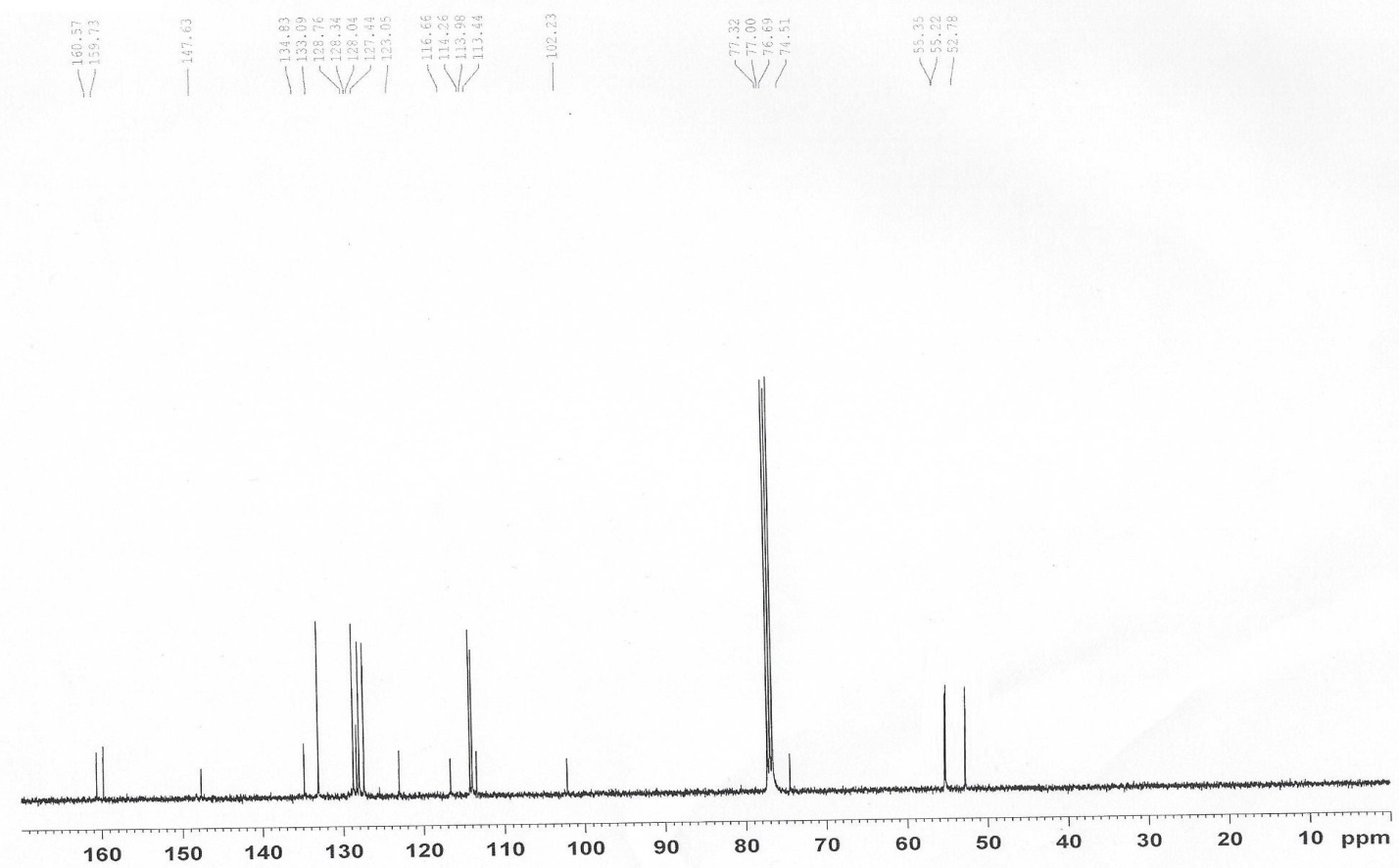


**Figure S14**. ^13^C NMR spectrum of **3ac** in CDCl_3_


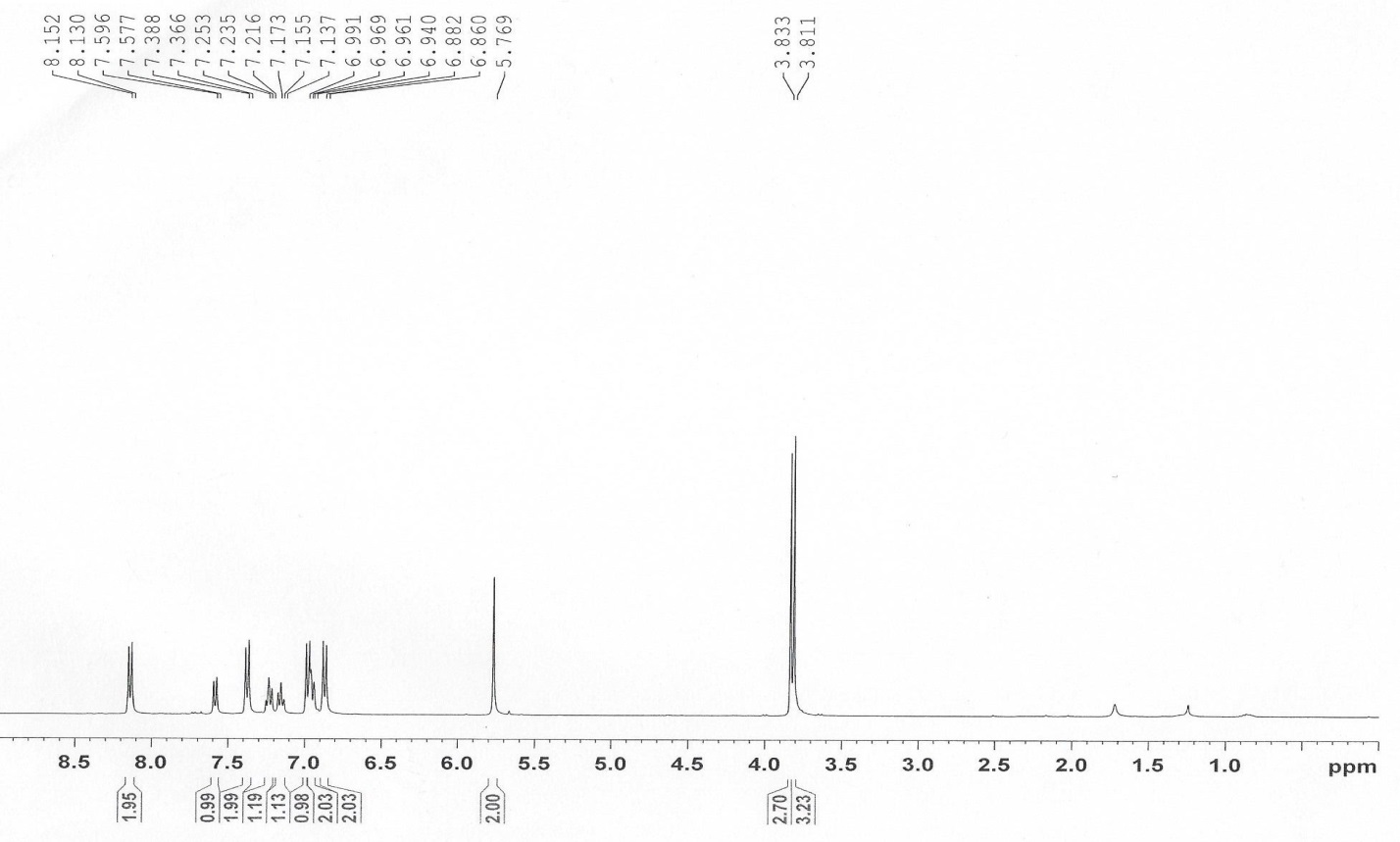


**Figure S15**. ^1^H NMR spectrum of **3bc** in CDCl_3_


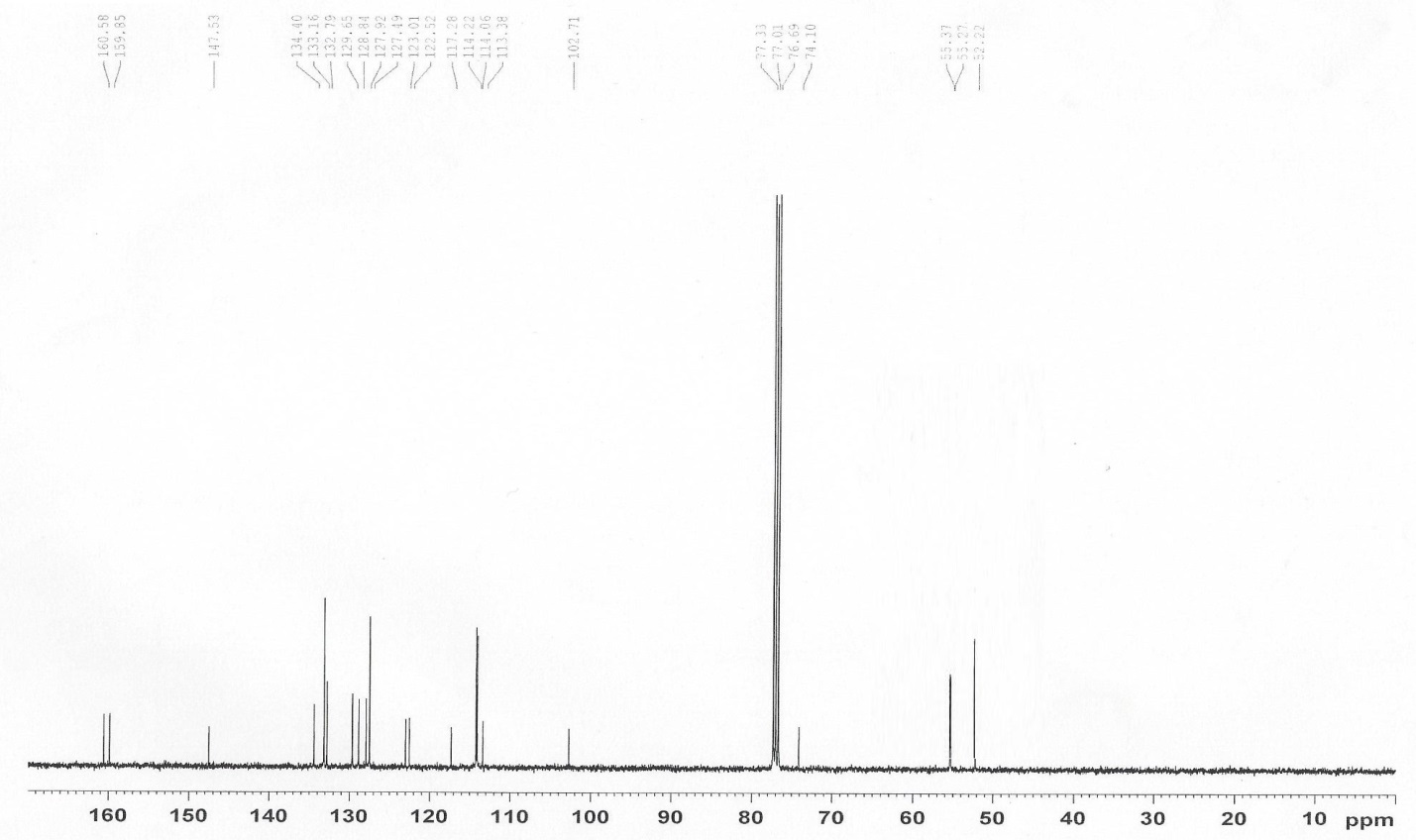


**Figure S16**. ^13^C NMR spectrum of **3bc** in CDCl_3_


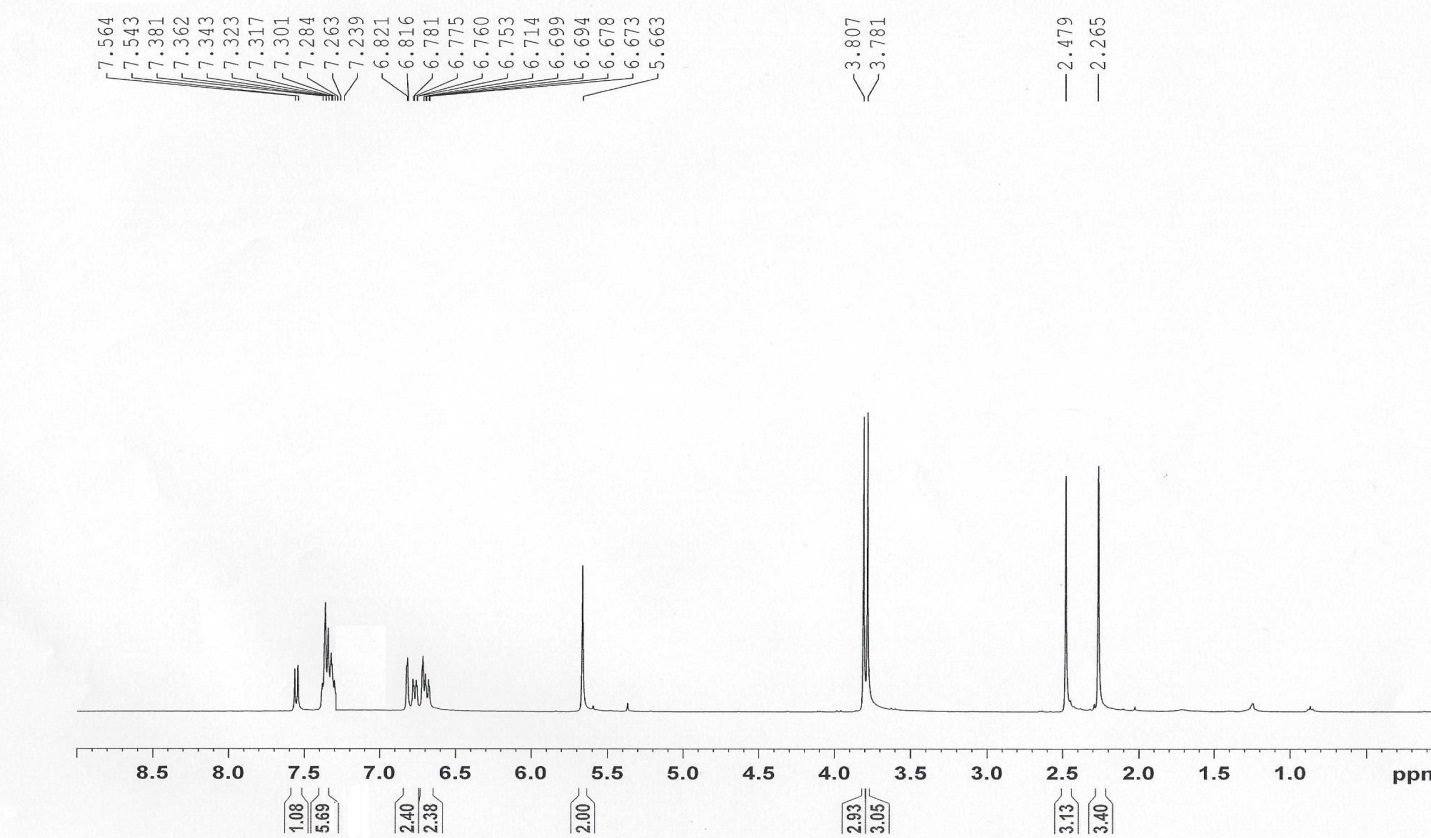


**Figure S17**. ^1^H NMR spectrum of **3ad** in CDCl_3_


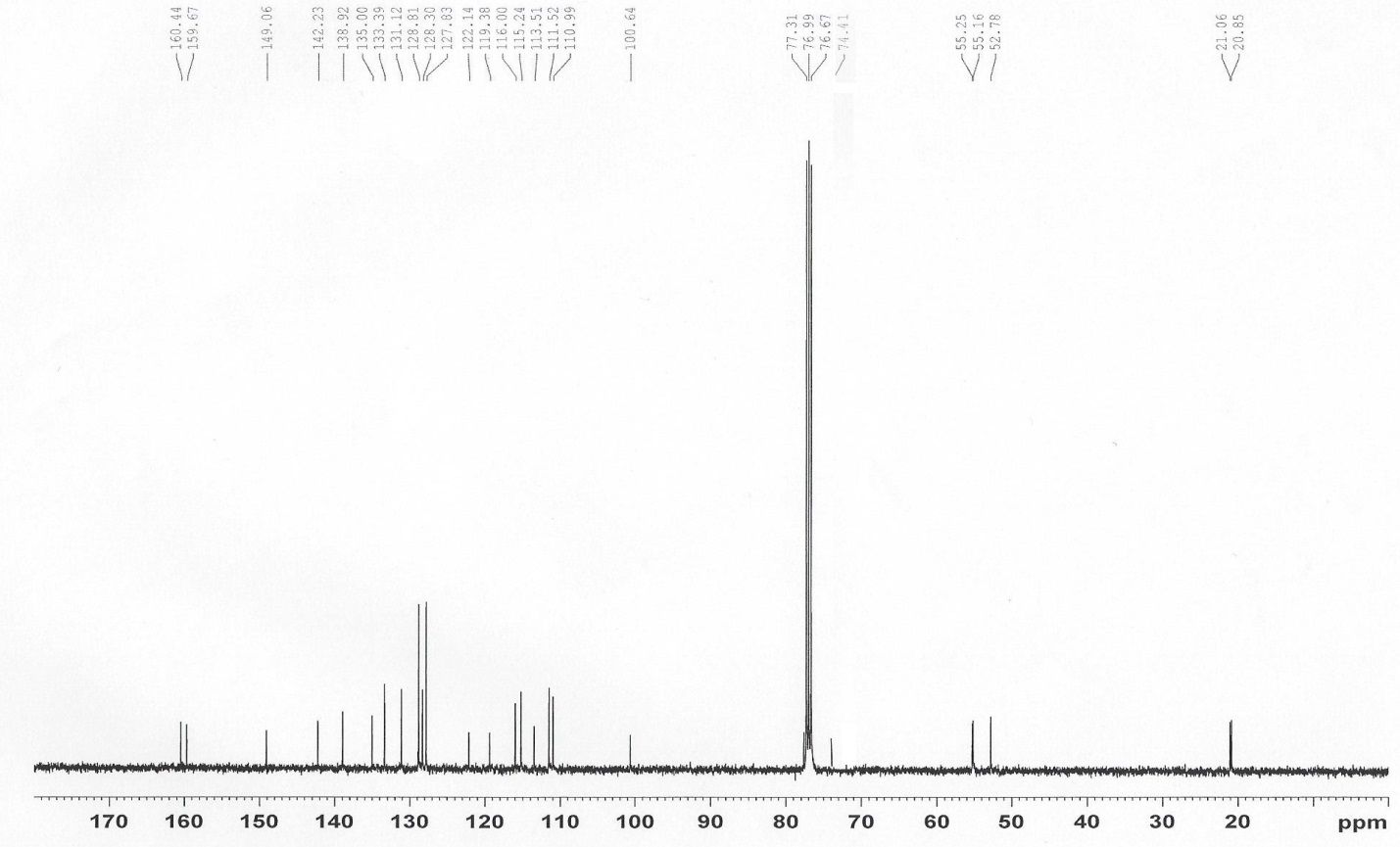


**Figure S18**. ^13^C NMR spectrum of **3ad** in CDCl_3_


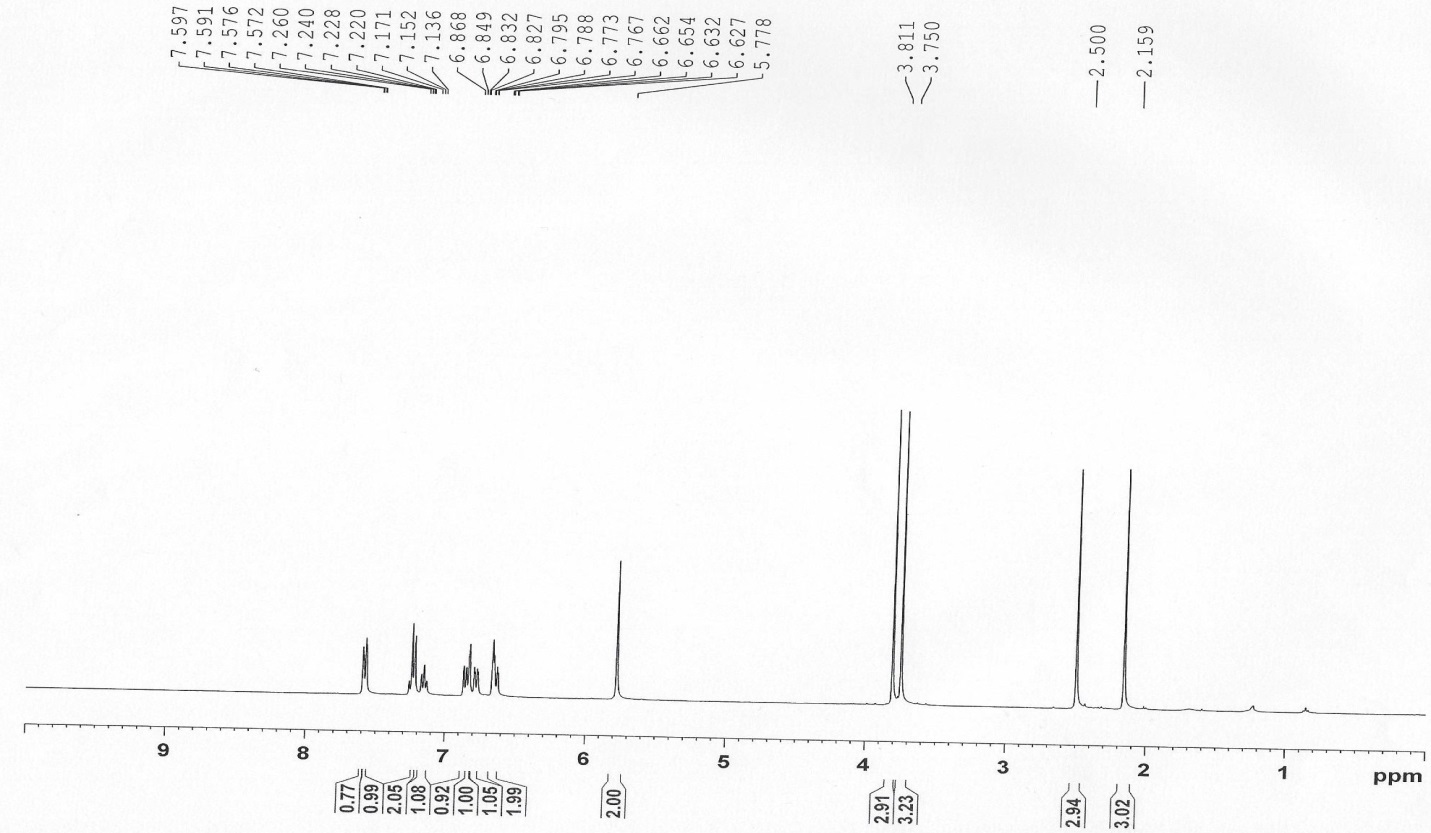


**Figure S19**. ^1^H NMR spectrum of **3bd** in CDCl_3_


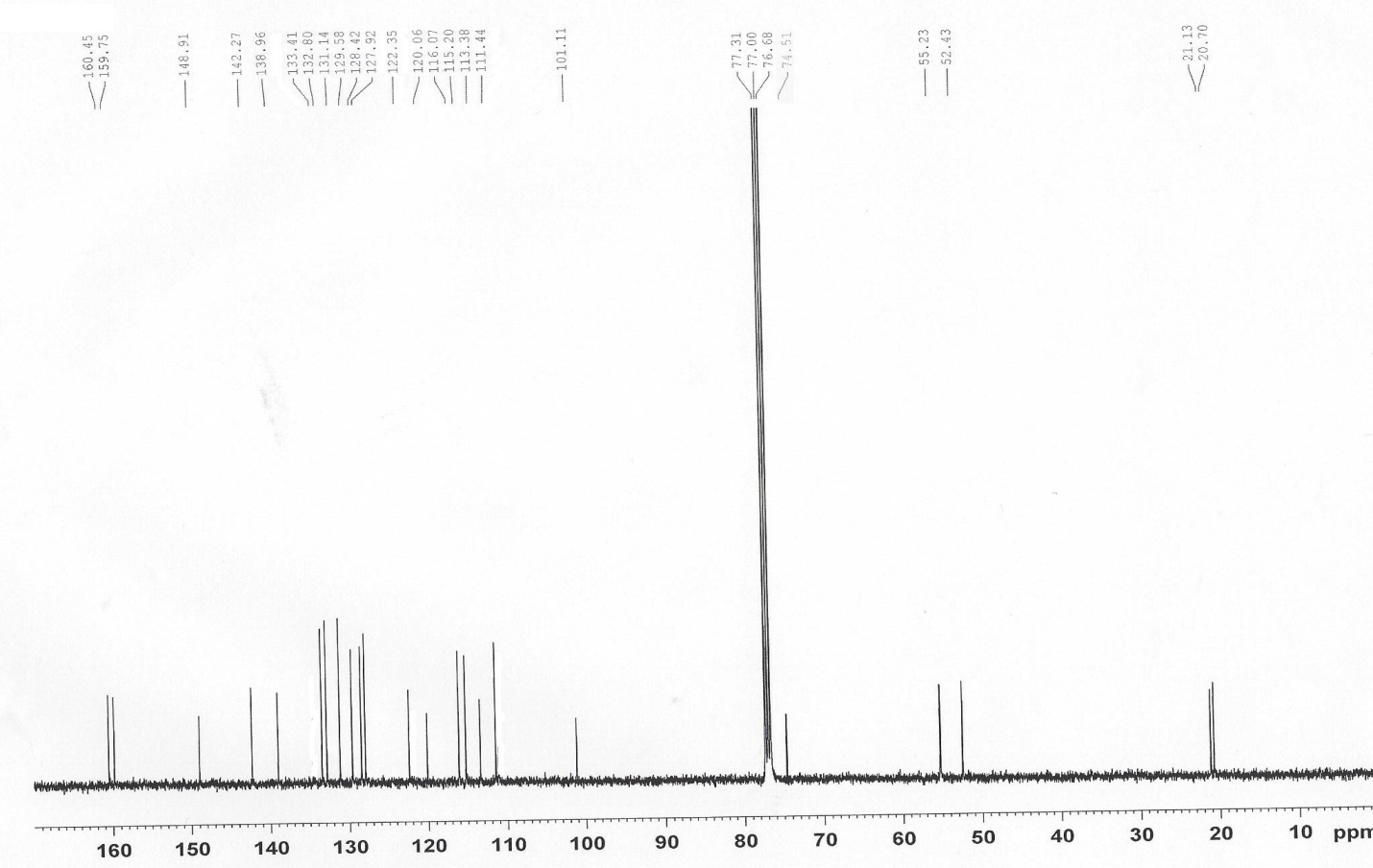


**Figure S20**. ^13^C NMR spectrum of **bad** in CDCl_3_


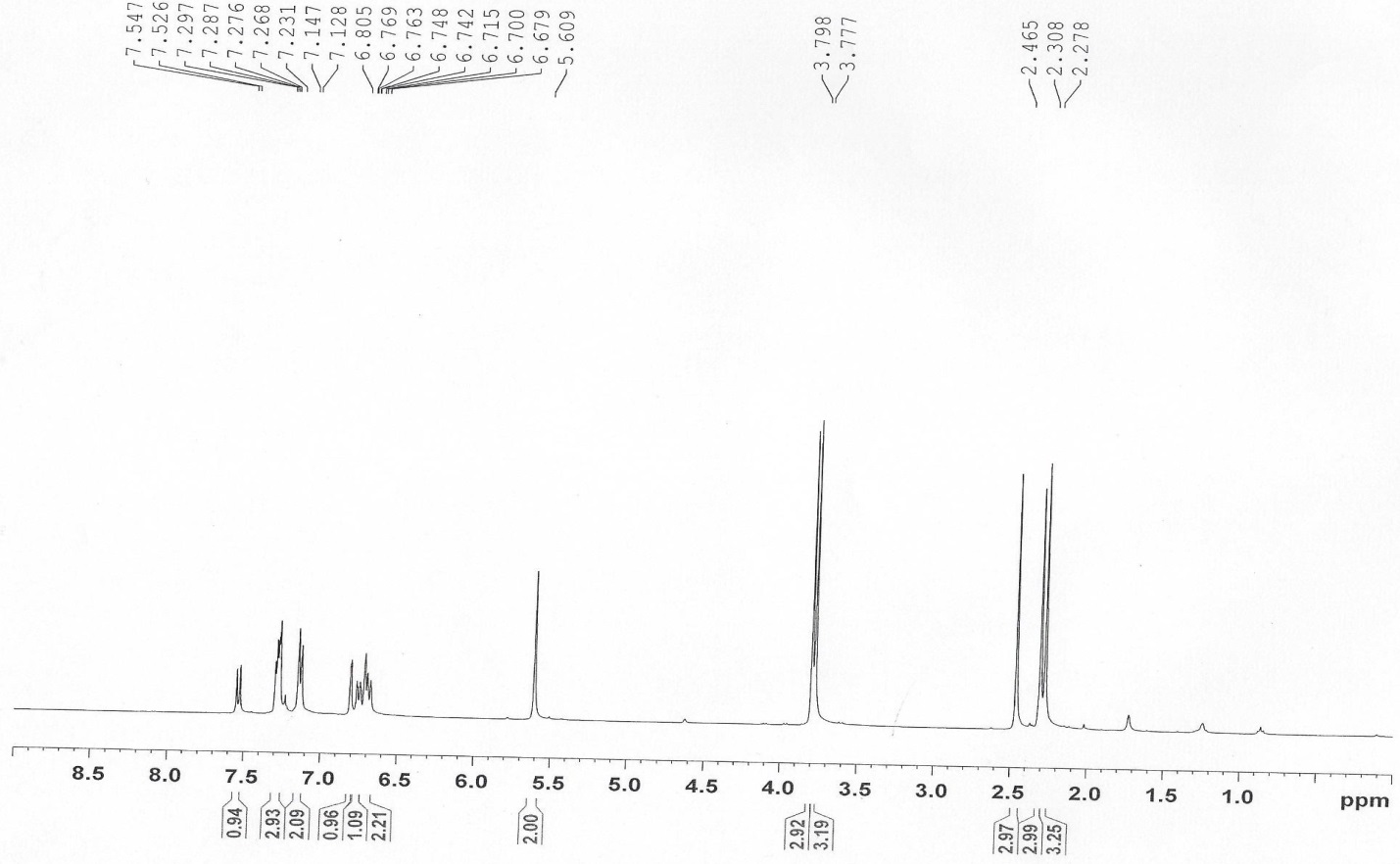


**Figure S21**. ^1^H NMR spectrum of **3cd** in CDCl_3_


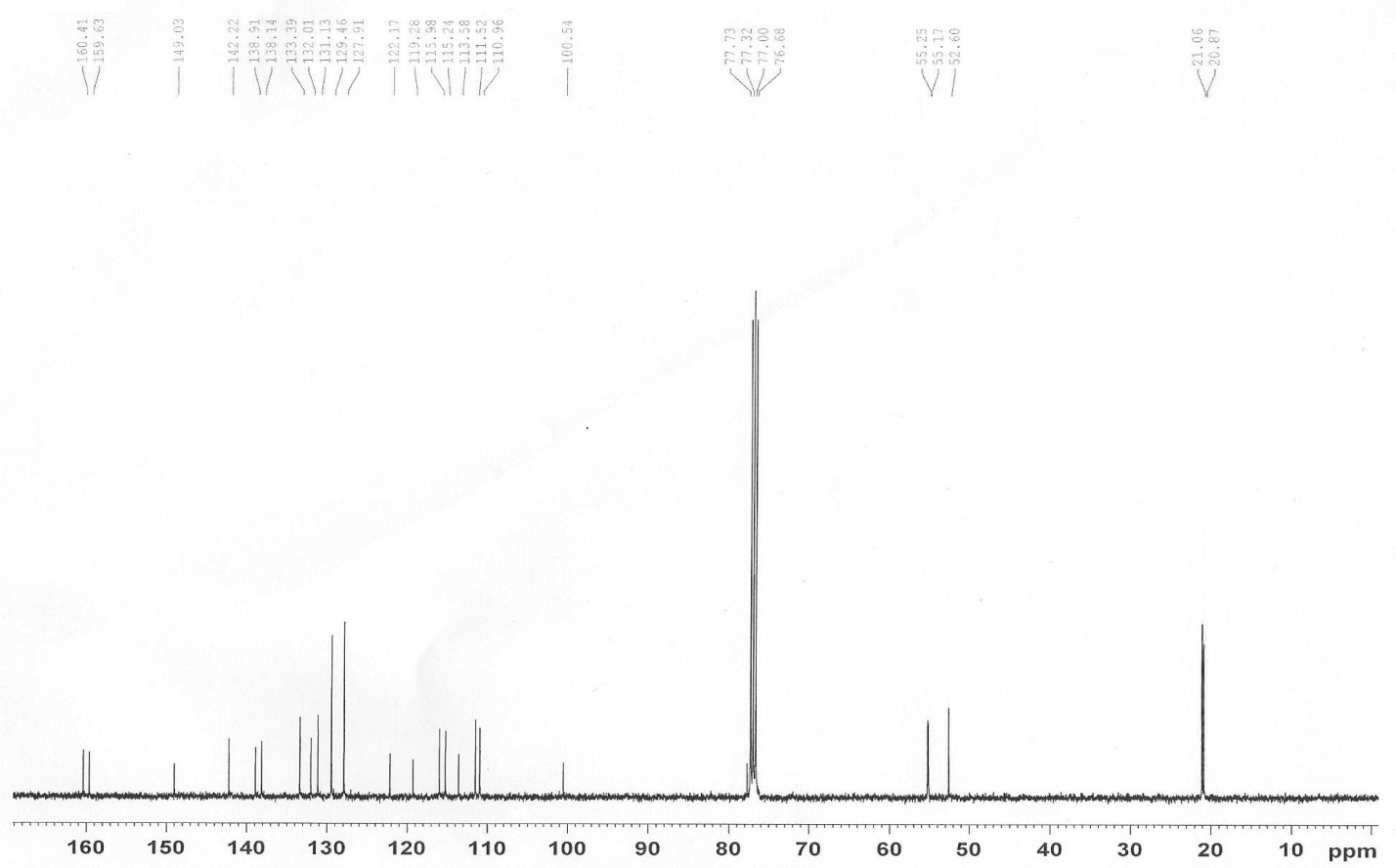


**Figure S22**. ^13^C NMR spectrum of **3cd** in CDCl_3_


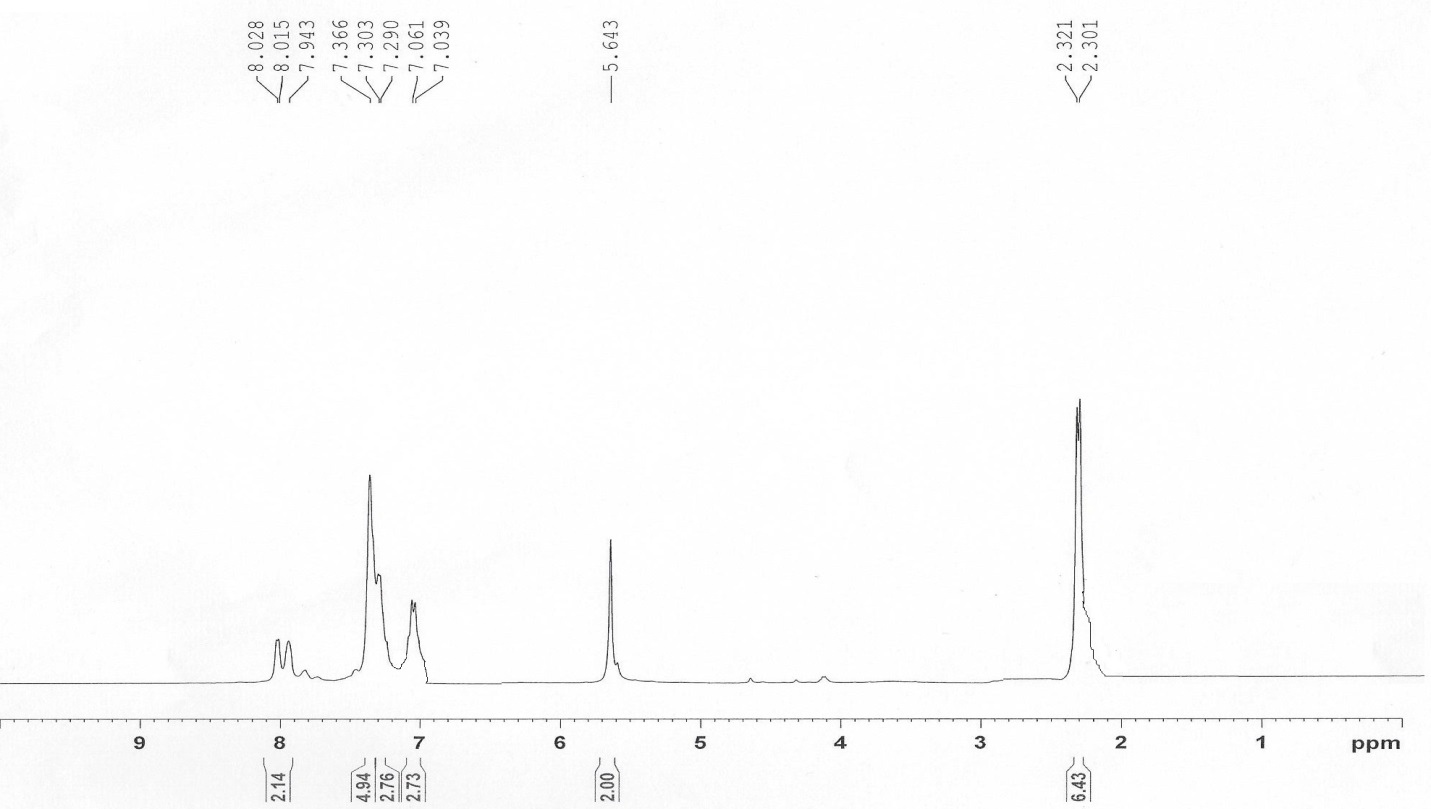


**Figure S23**. ^1^H NMR spectrum of **3ae** in CDCl_3_


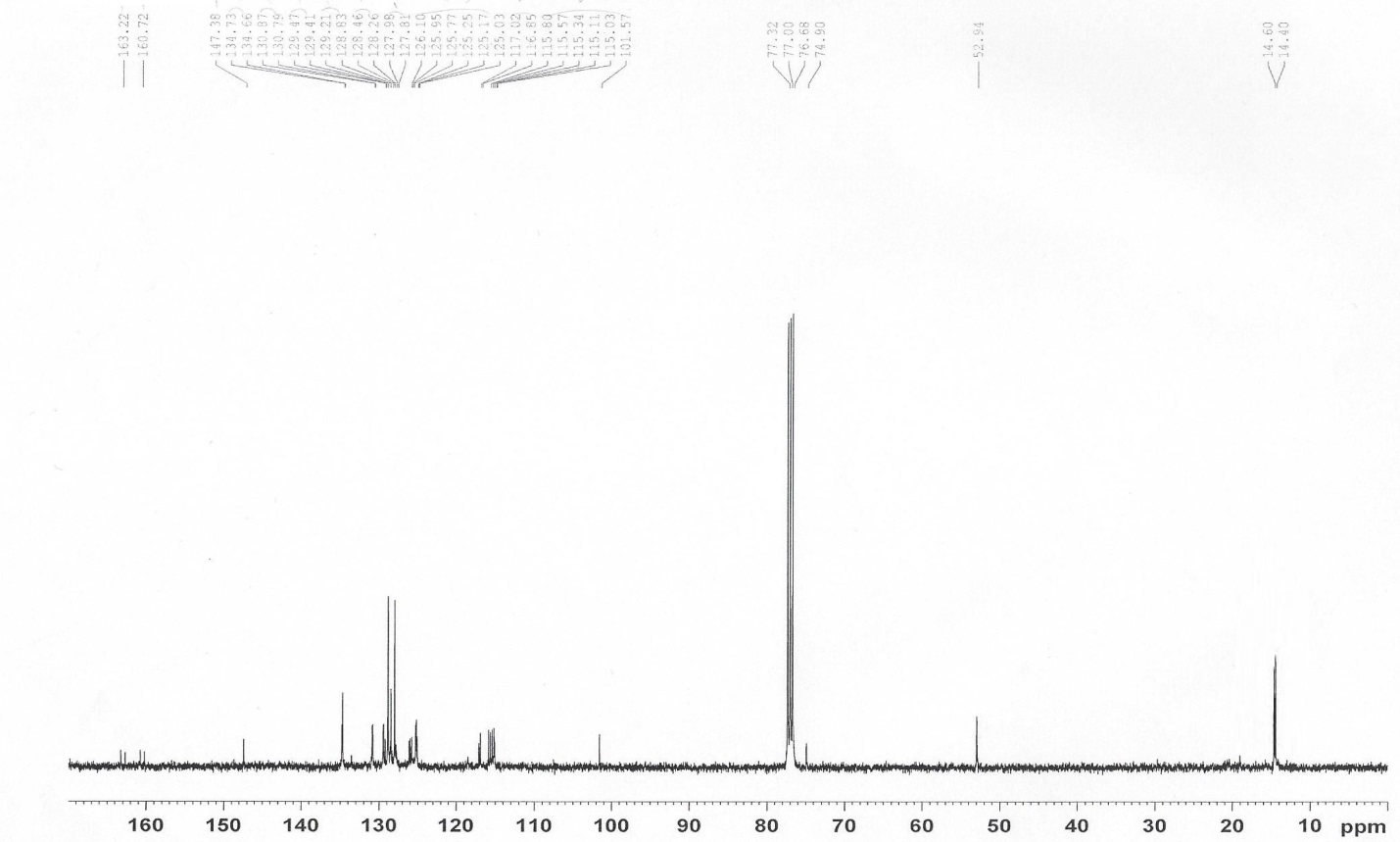


**Figure S24**. ^13^C NMR spectrum of **3ae** in CDCl_3_


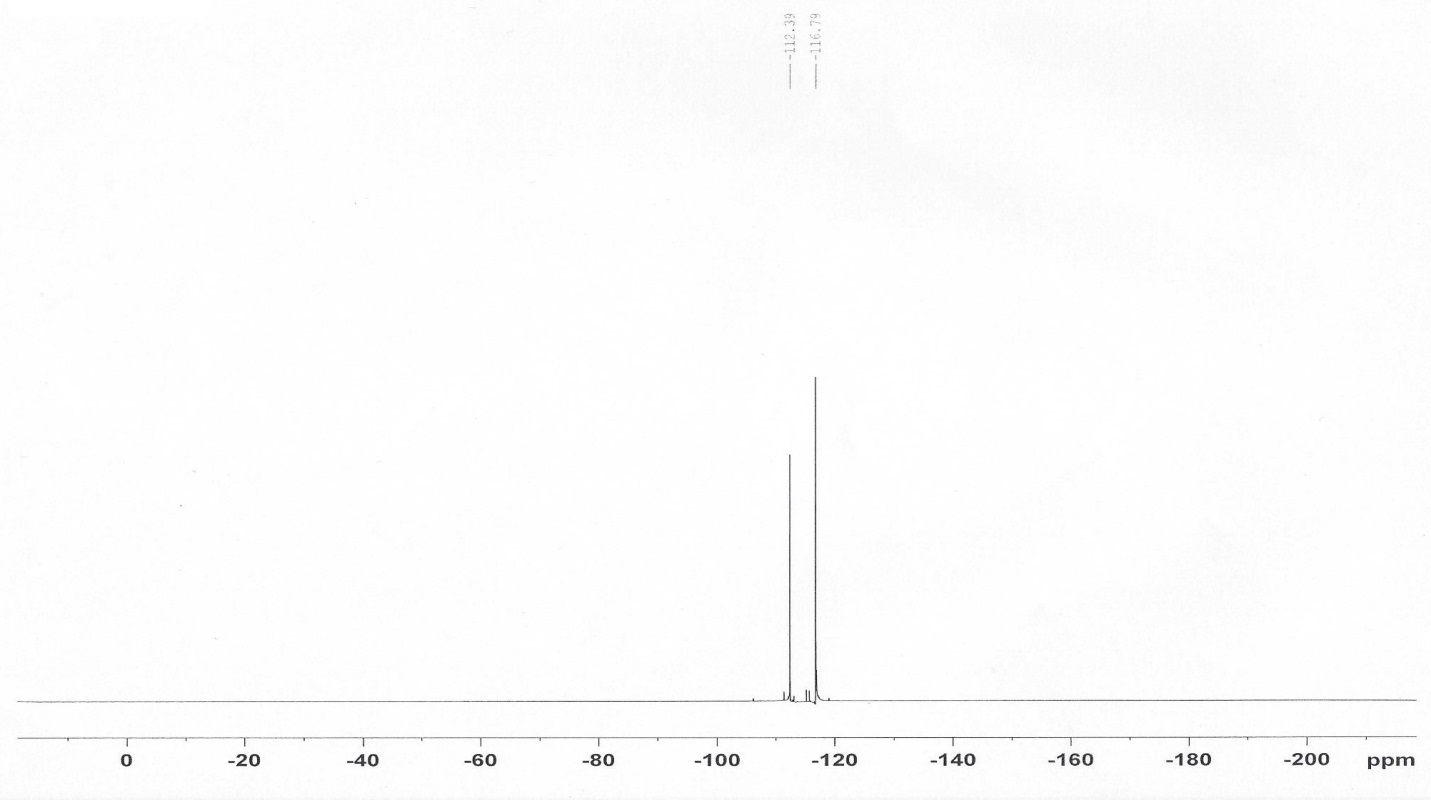


**Figure S25**. ^19^F NMR spectrum of **3ae** in CDCl_3_


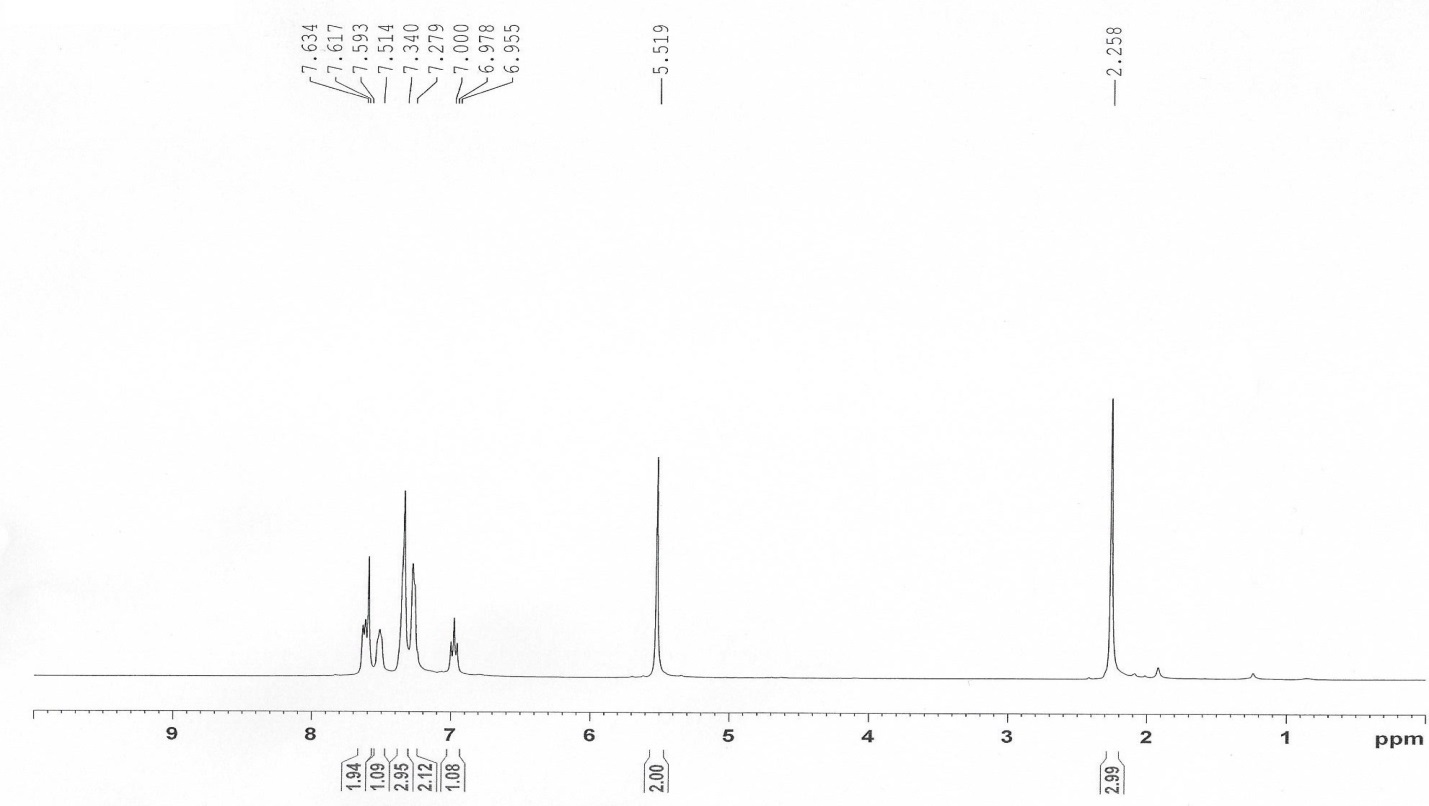


**Figure S26**. ^1^H NMR spectrum of **3ae'** in CDCl_3_


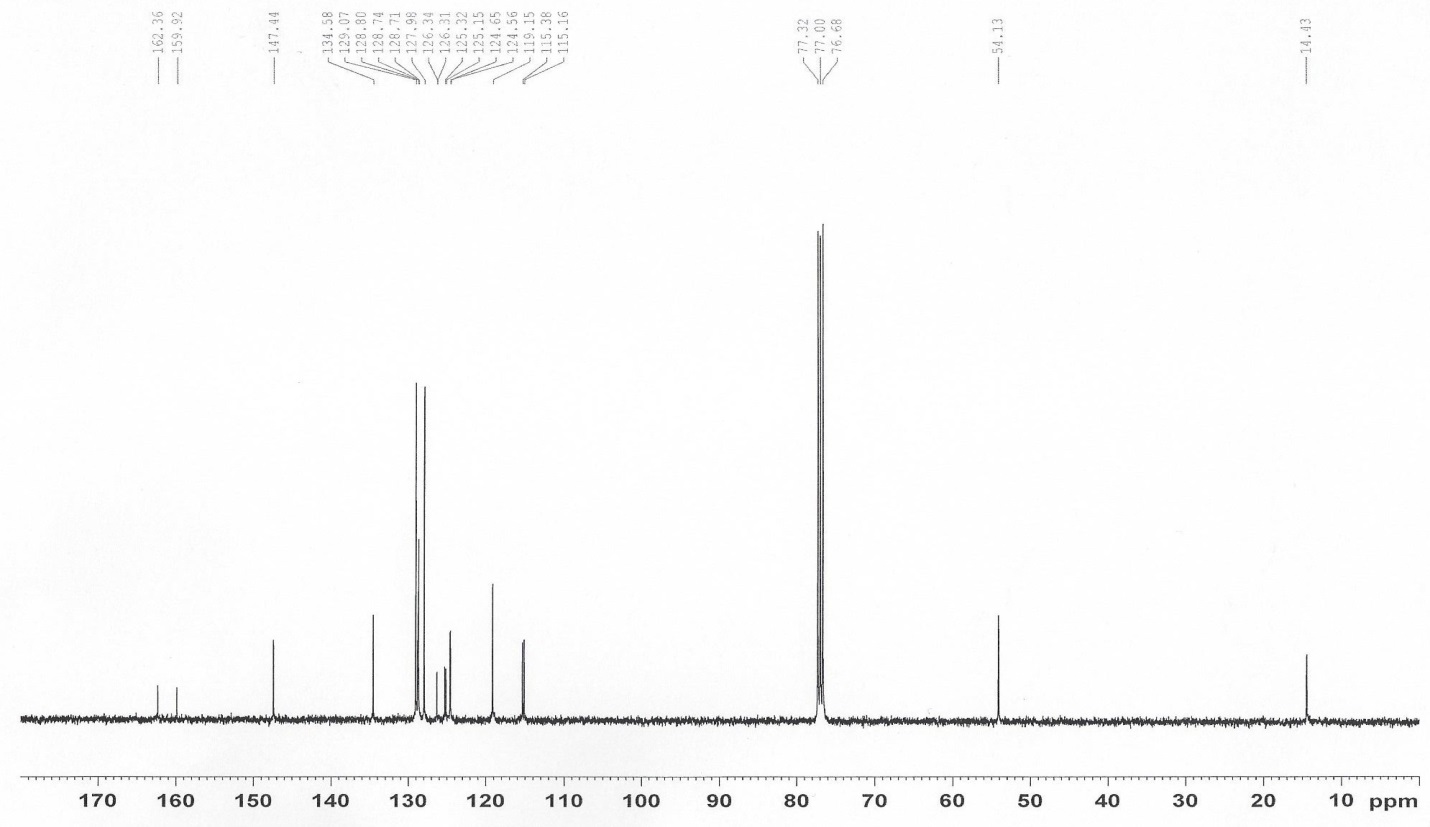


**Figure S27**. ^13^C NMR spectrum of **3ae'** in CDCl_3_


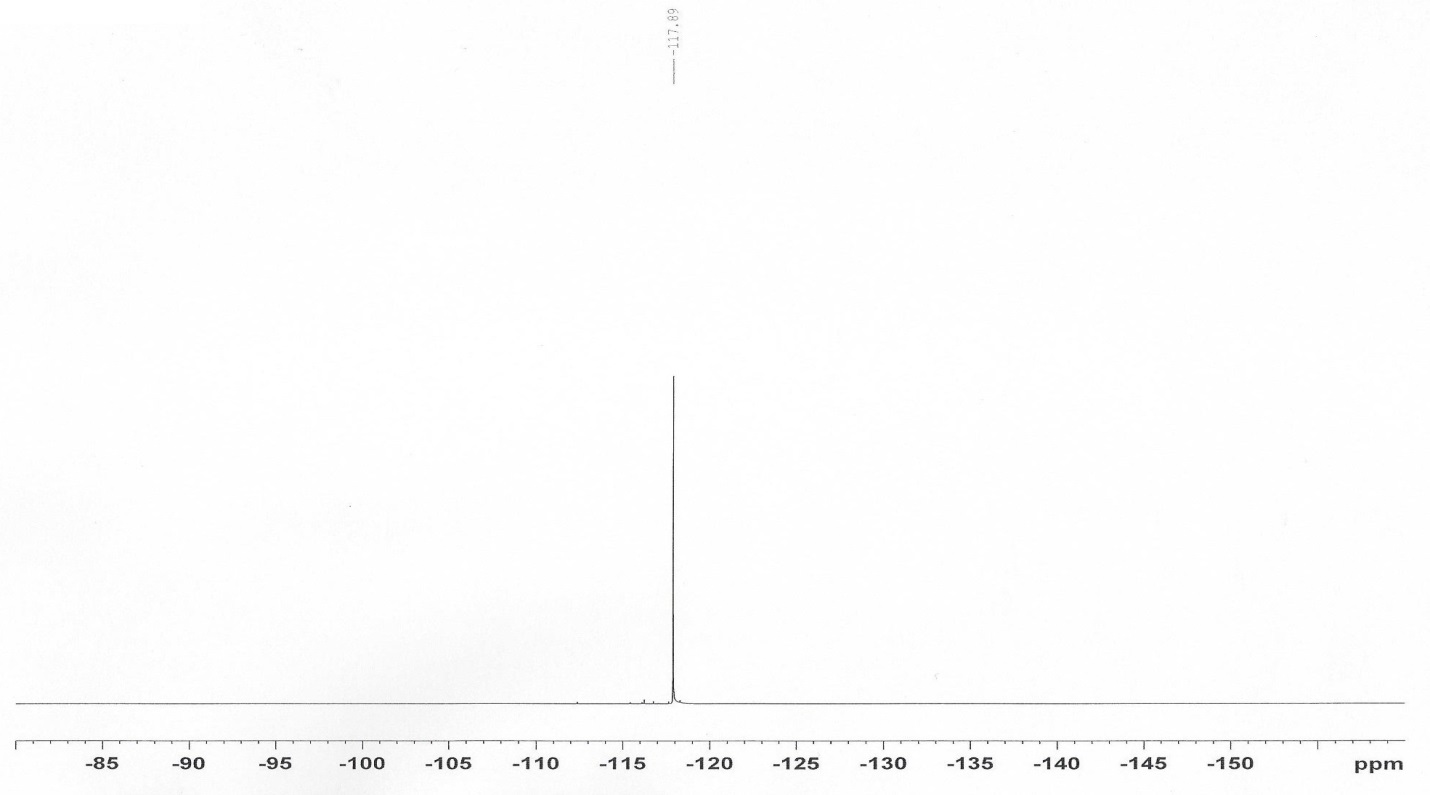


**Figure S28**. ^19^F NMR spectrum of **3ae'** in CDCl_3_


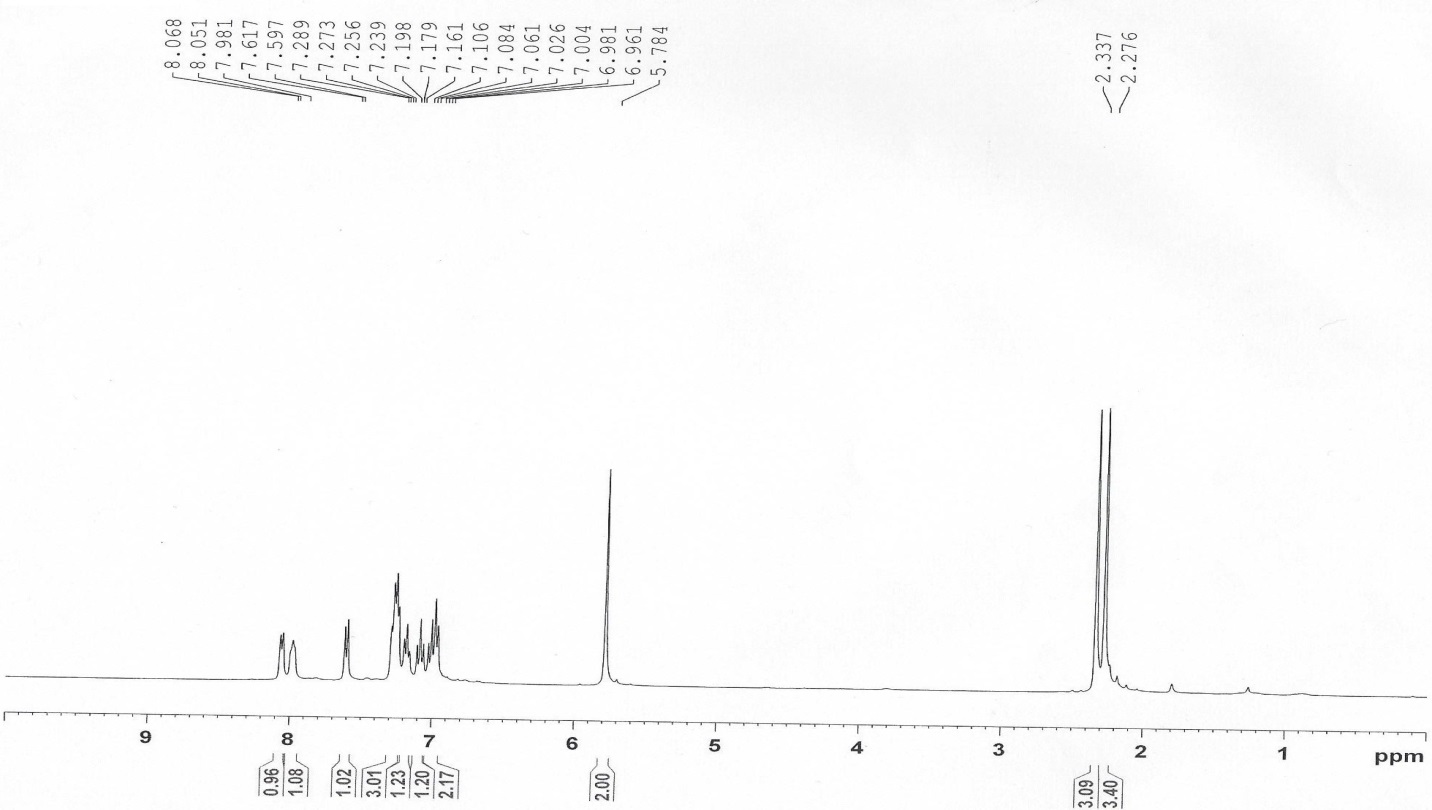


**Figure S29**. ^1^H NMR spectrum of **3be** in CDCl_3_


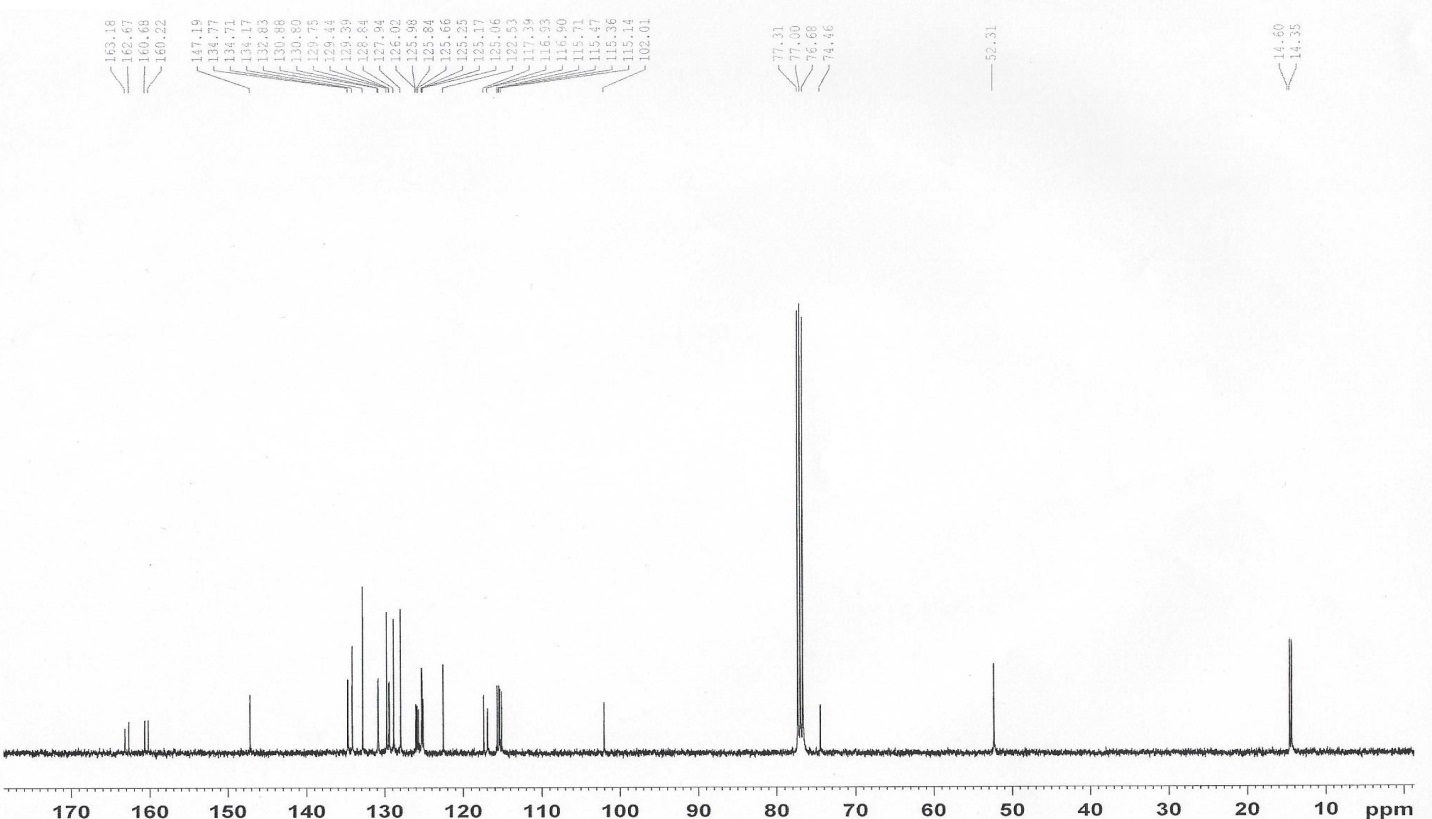


**Figure S30**. ^13^C NMR spectrum of **3be** in CDCl_3_


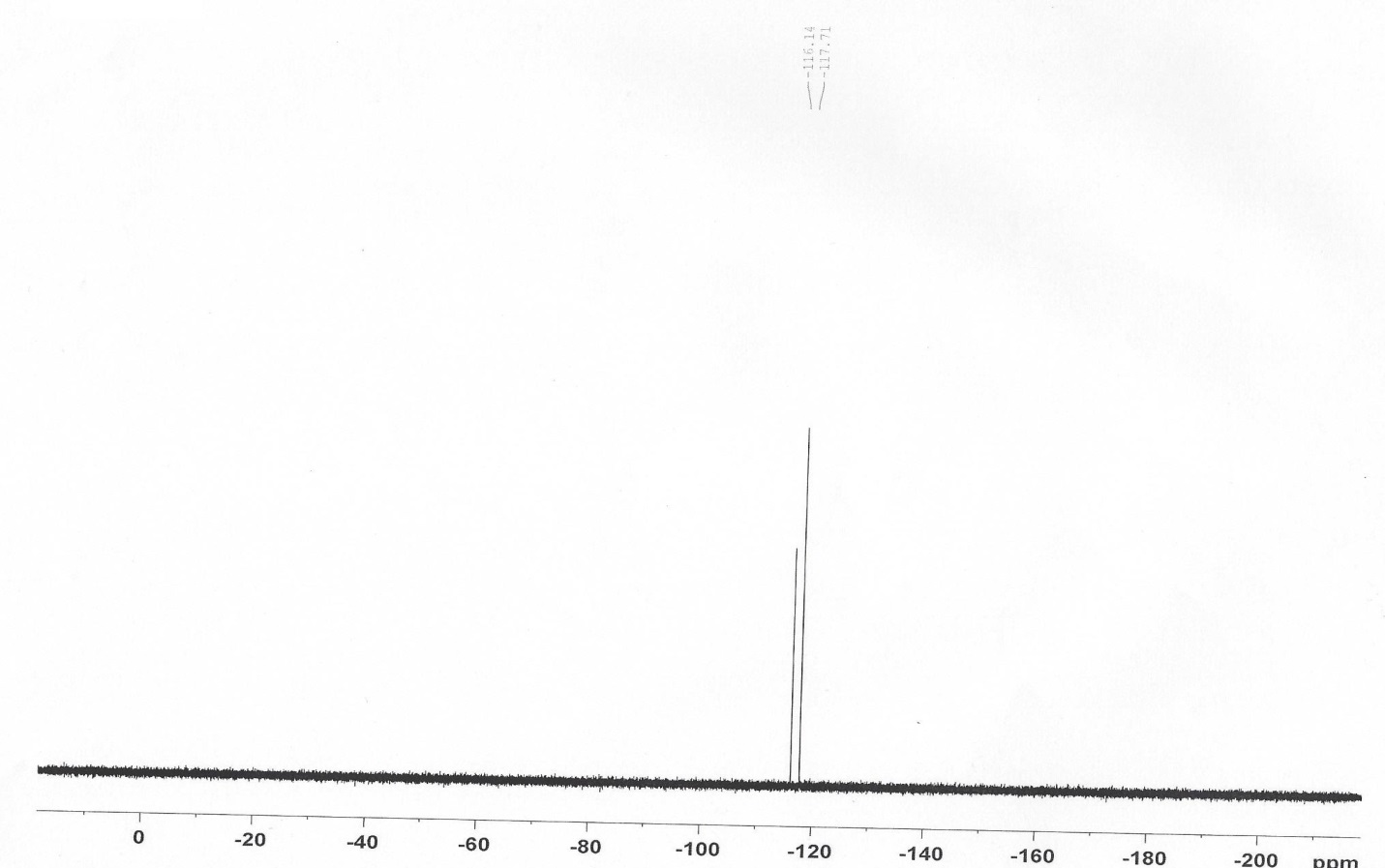


**Figure S31**. ^19^F NMR spectrum of **3be** in CDCl_3_


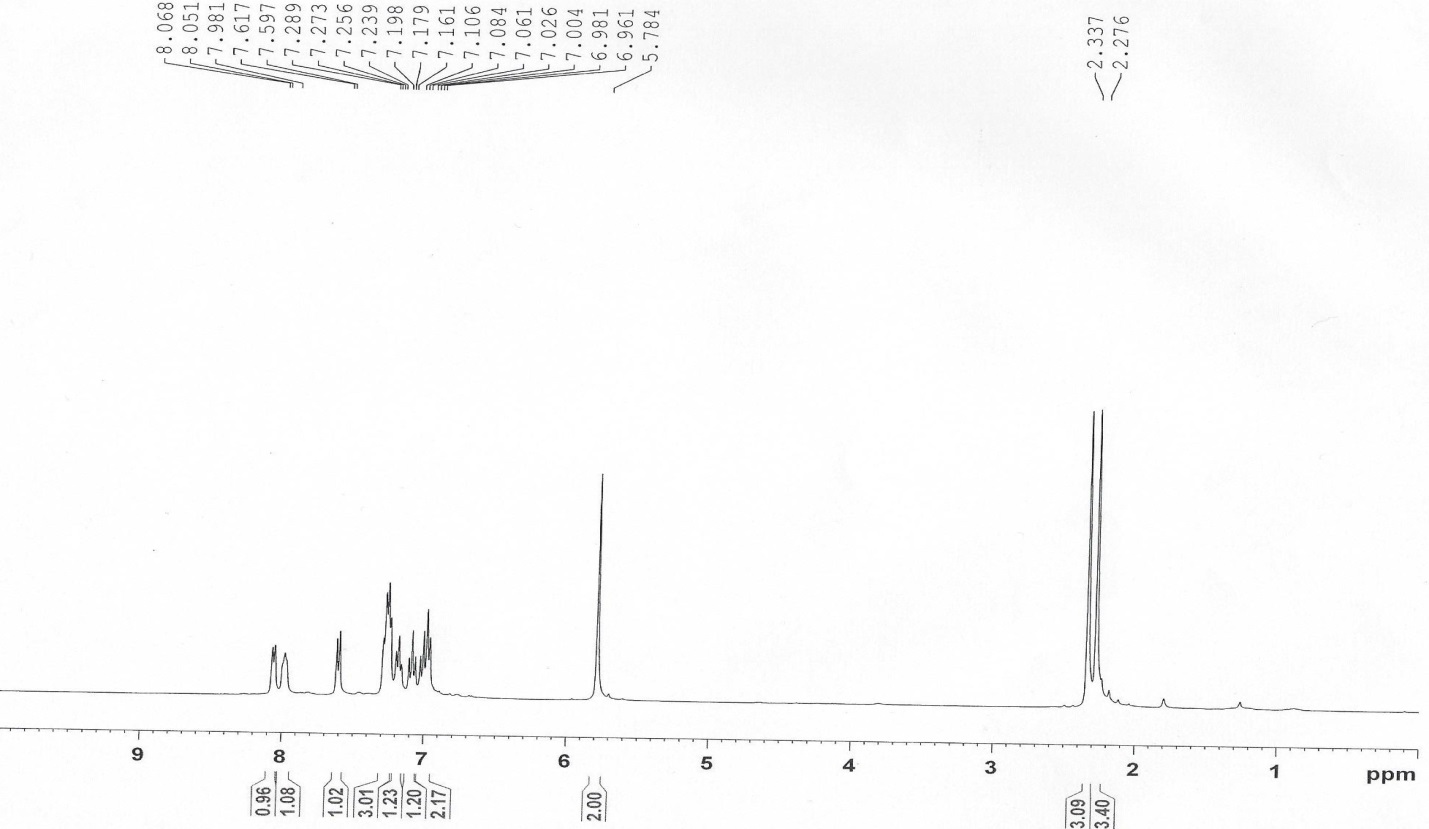


**Figure S32**. ^1^H NMR spectrum of **3ce** in CDCl_3_


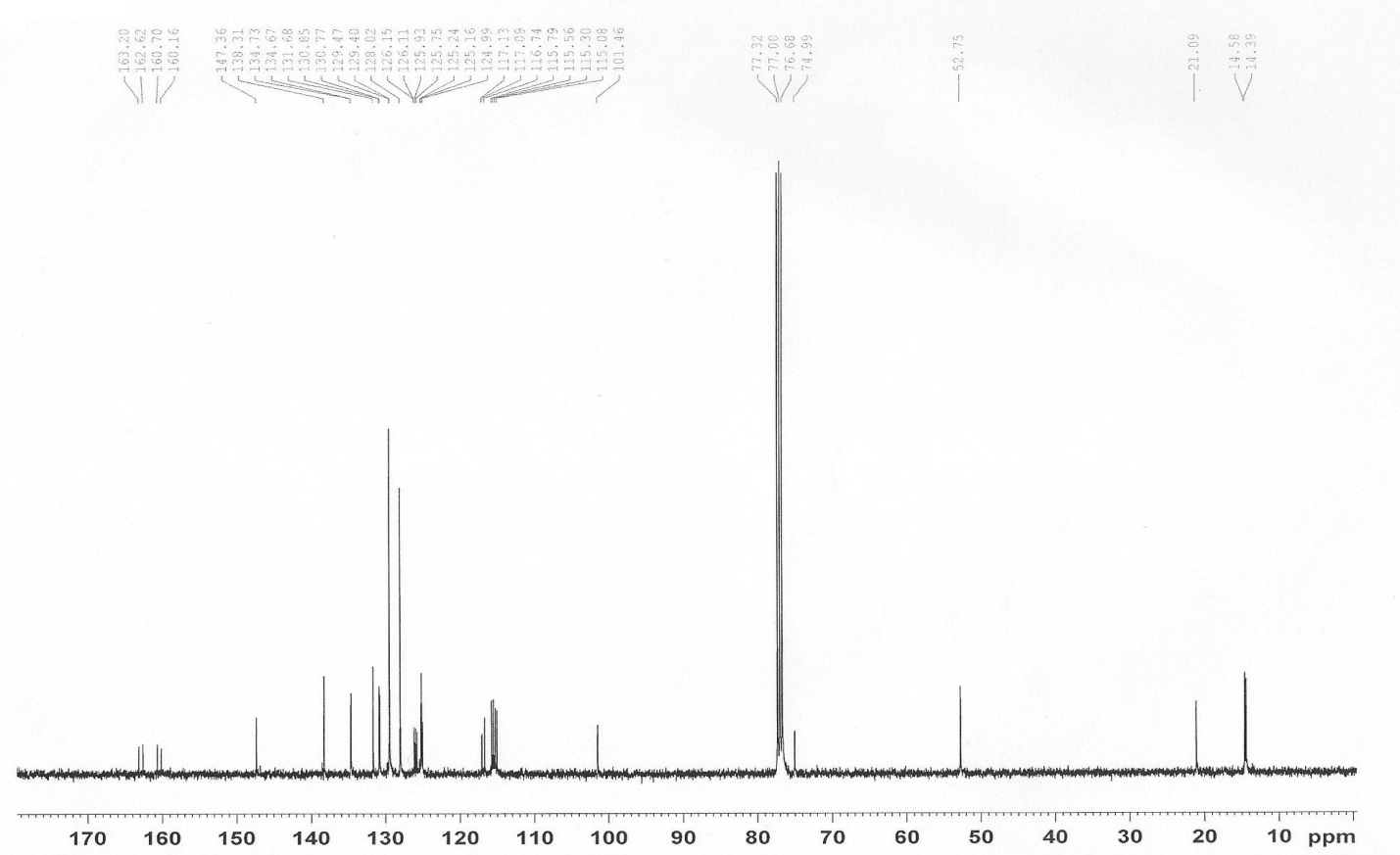


**Figure S33**. ^13^C NMR spectrum of **3ce** in CDCl_3_


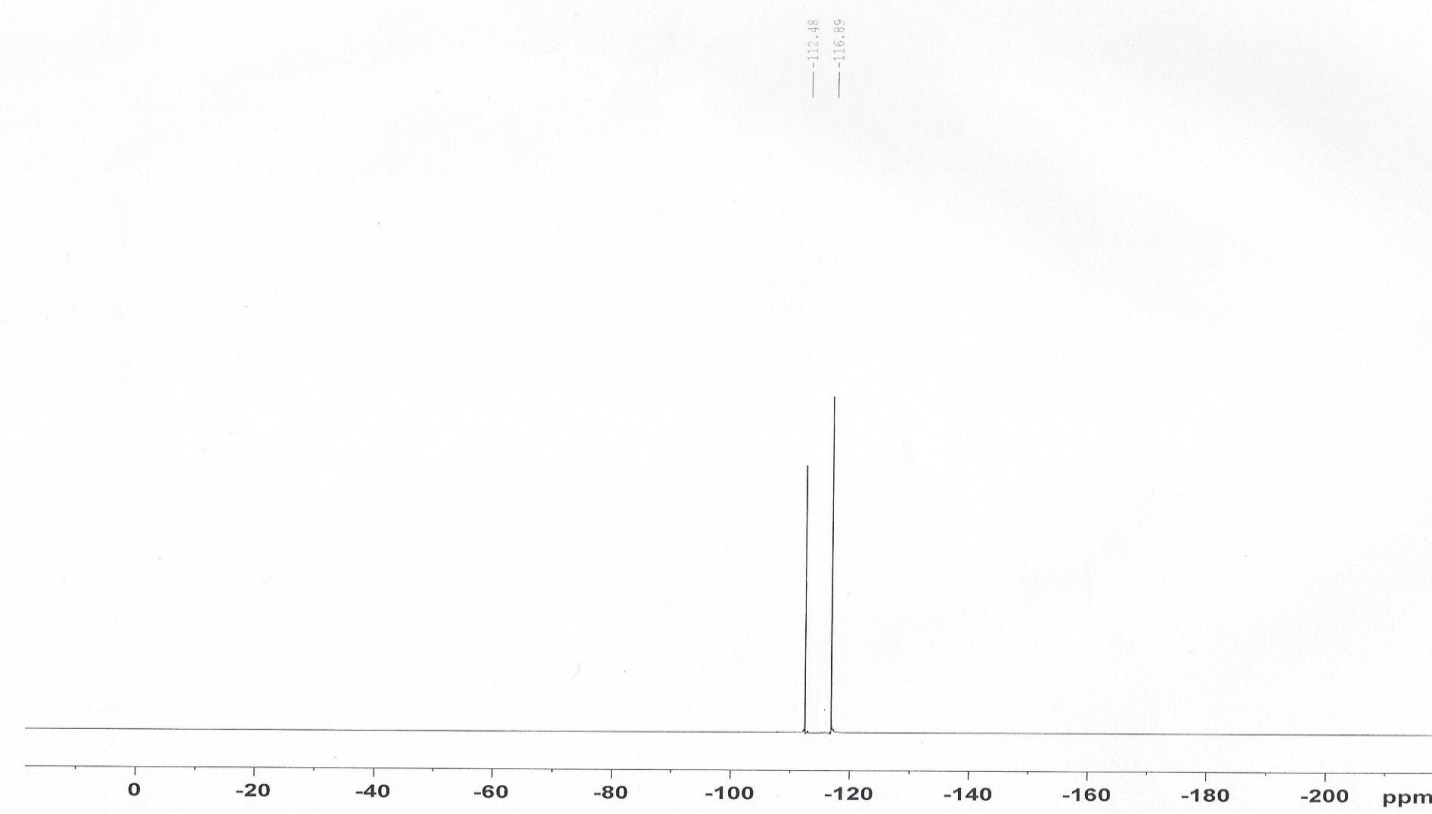


**Figure S34**. ^19^F NMR spectrum of **3be** in CDCl_3_


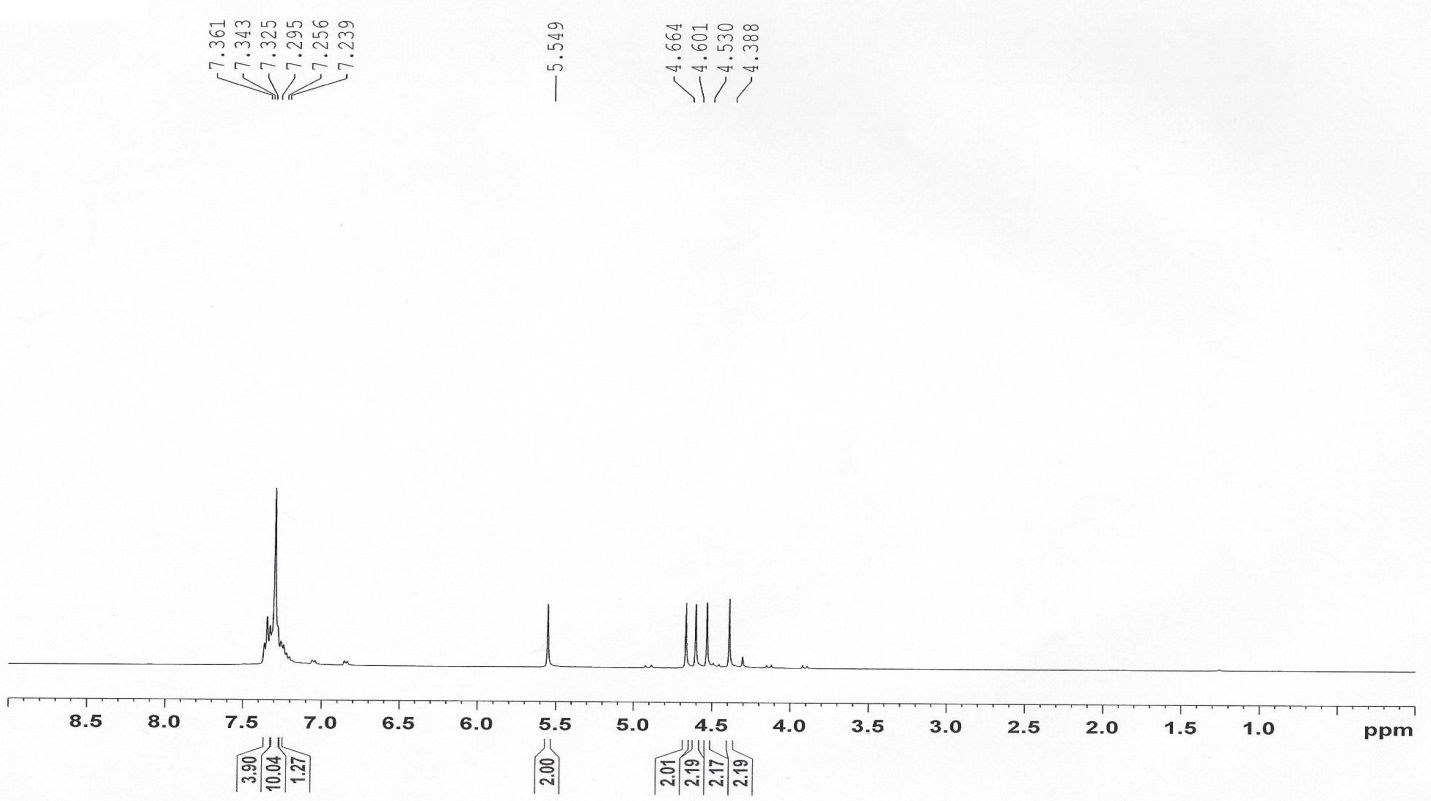


**Figure S35**. ^1^H NMR spectrum of **3af** in CDCl_3_


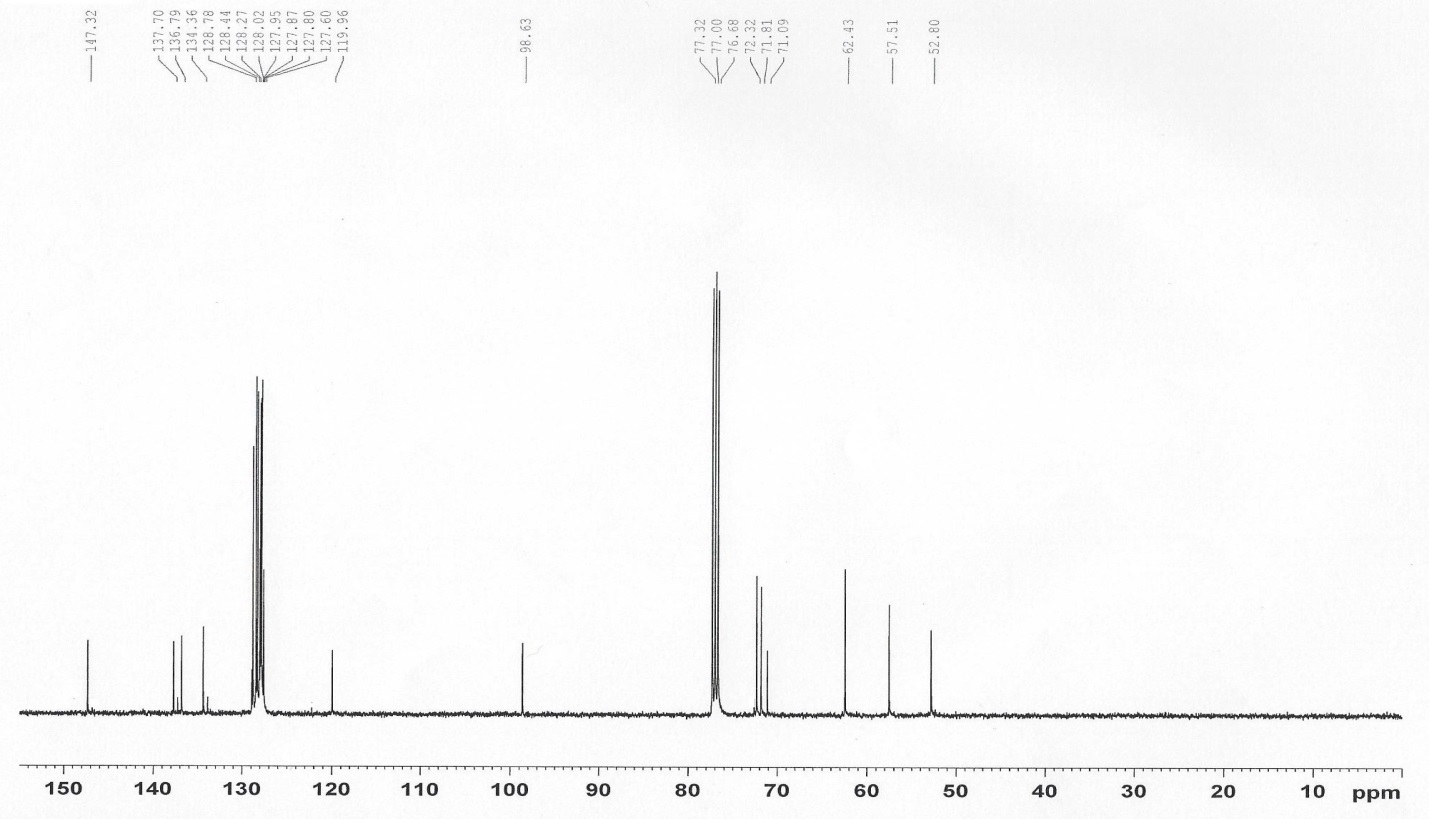


**Figure S36**. ^13^C NMR spectrum of **3af** in CDCl_3_


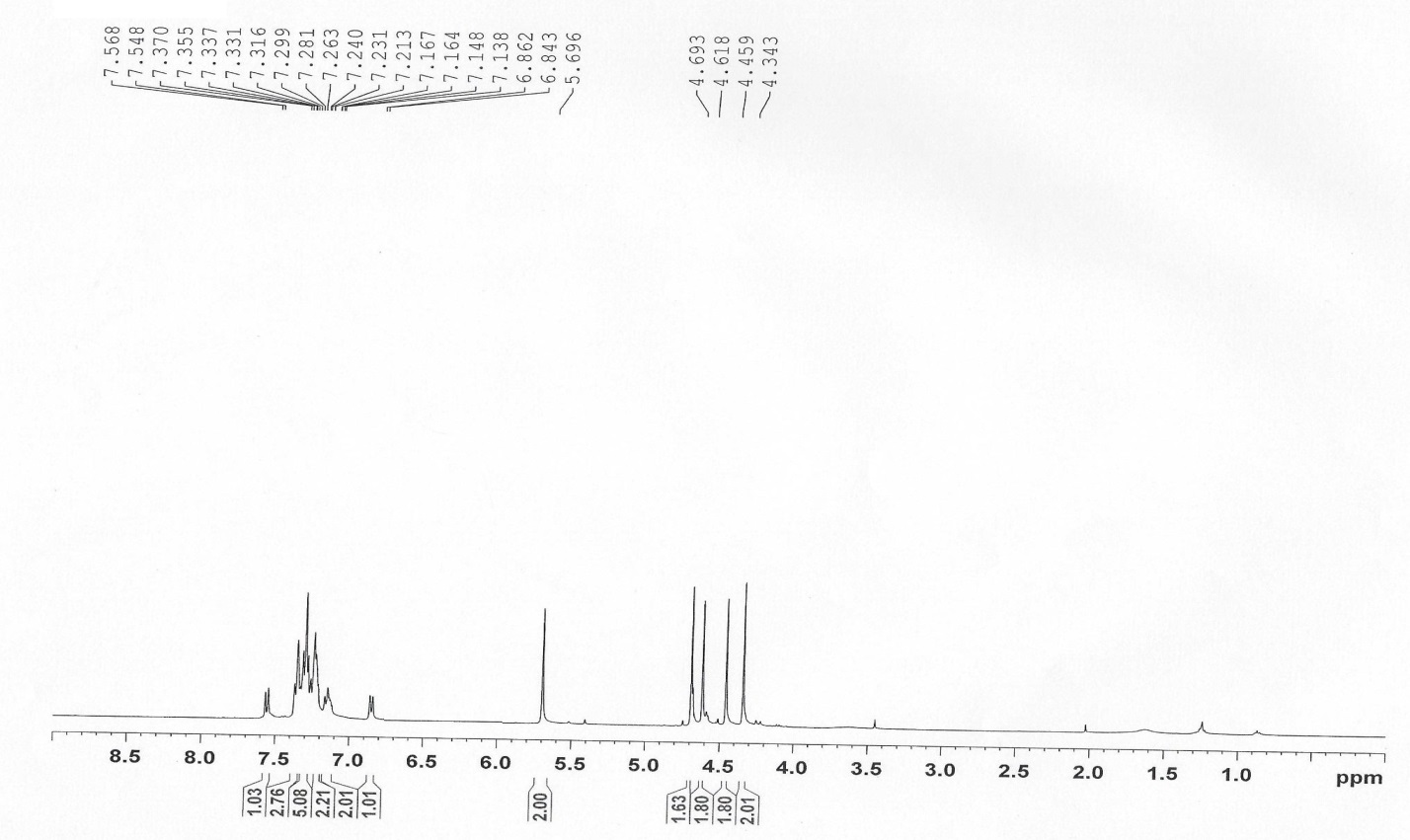


**Figure S37**. ^1^H NMR spectrum of **3bf** in CDCl_3_


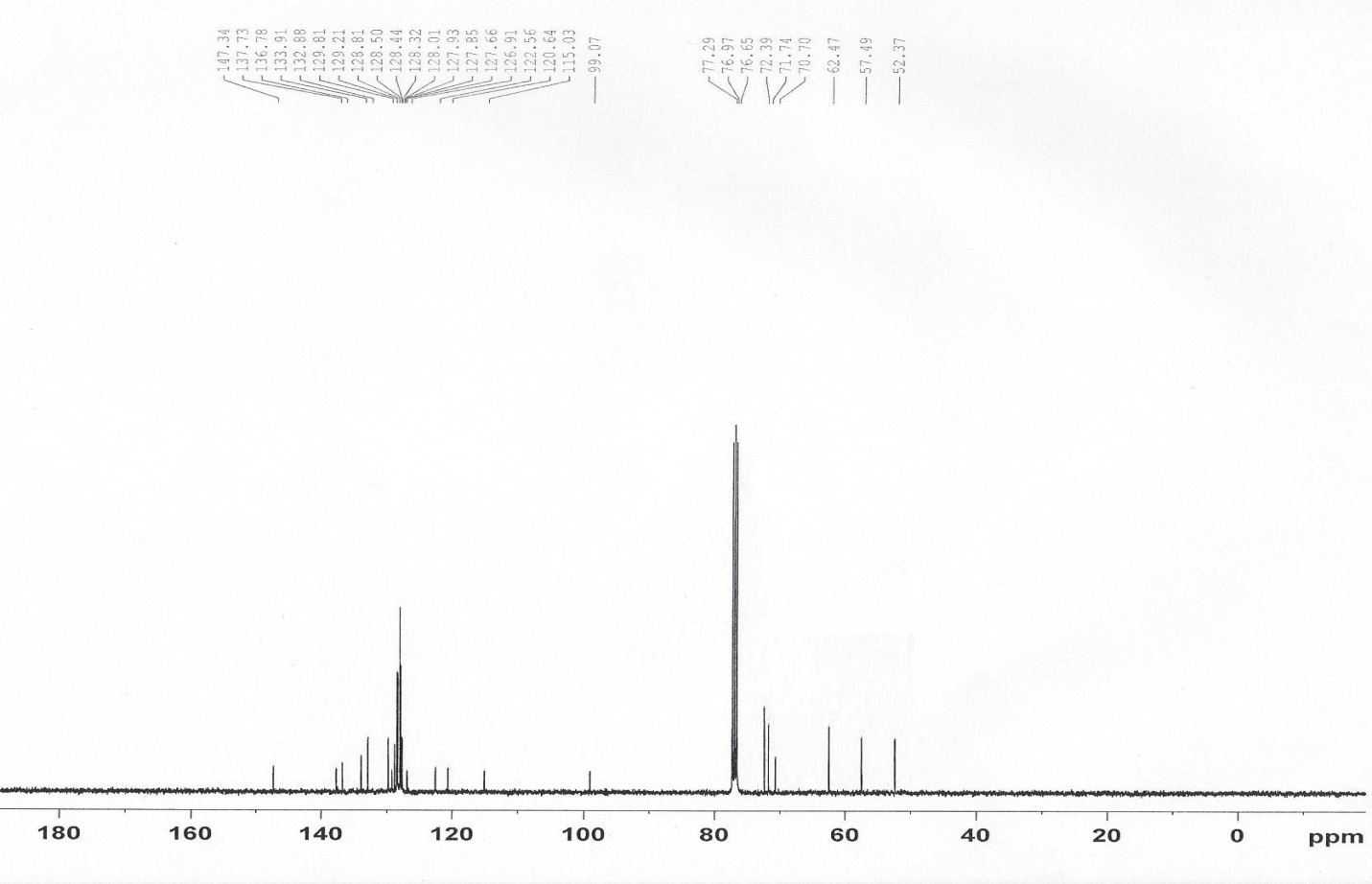


**Figure S38**. ^13^C NMR spectrum of **3bf** in CDCl_3_


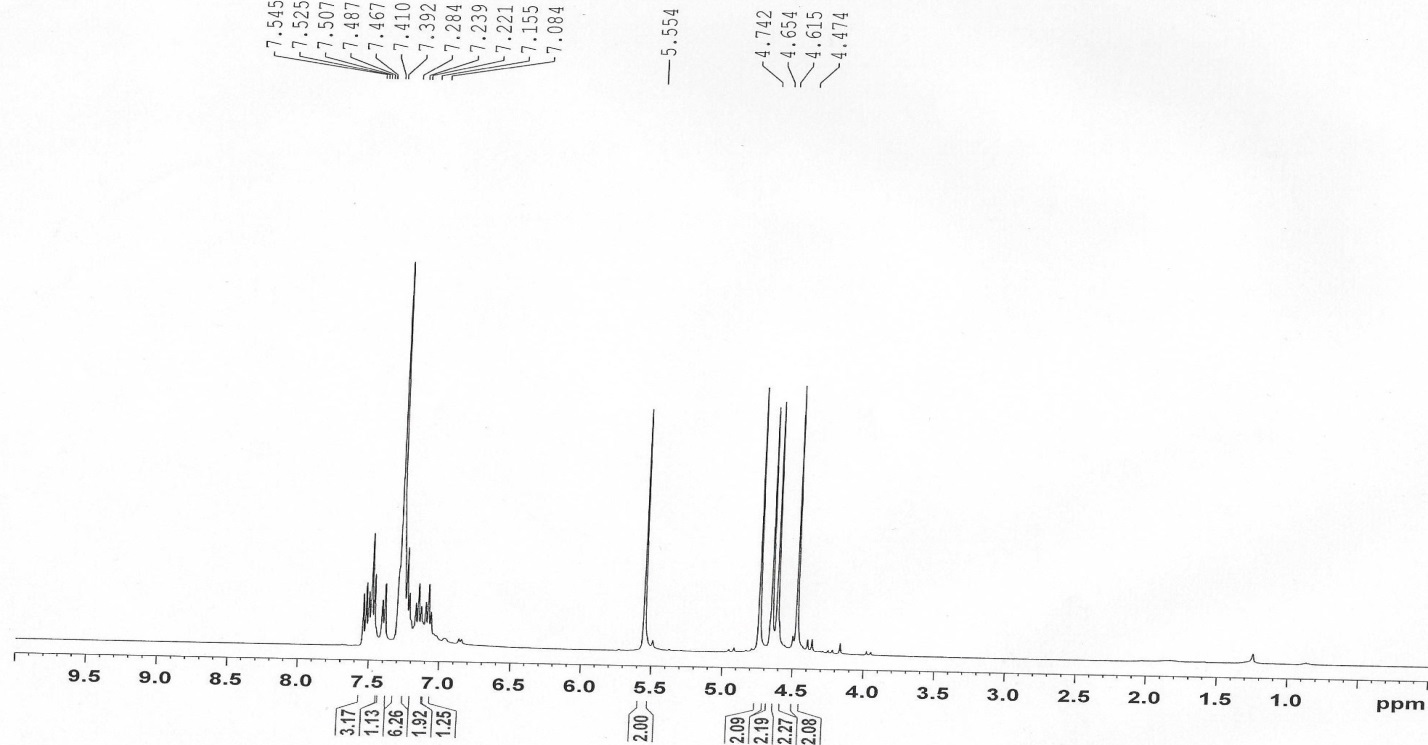


**Figure S39**. ^1^H NMR spectrum of **3ag** in CDCl_3_


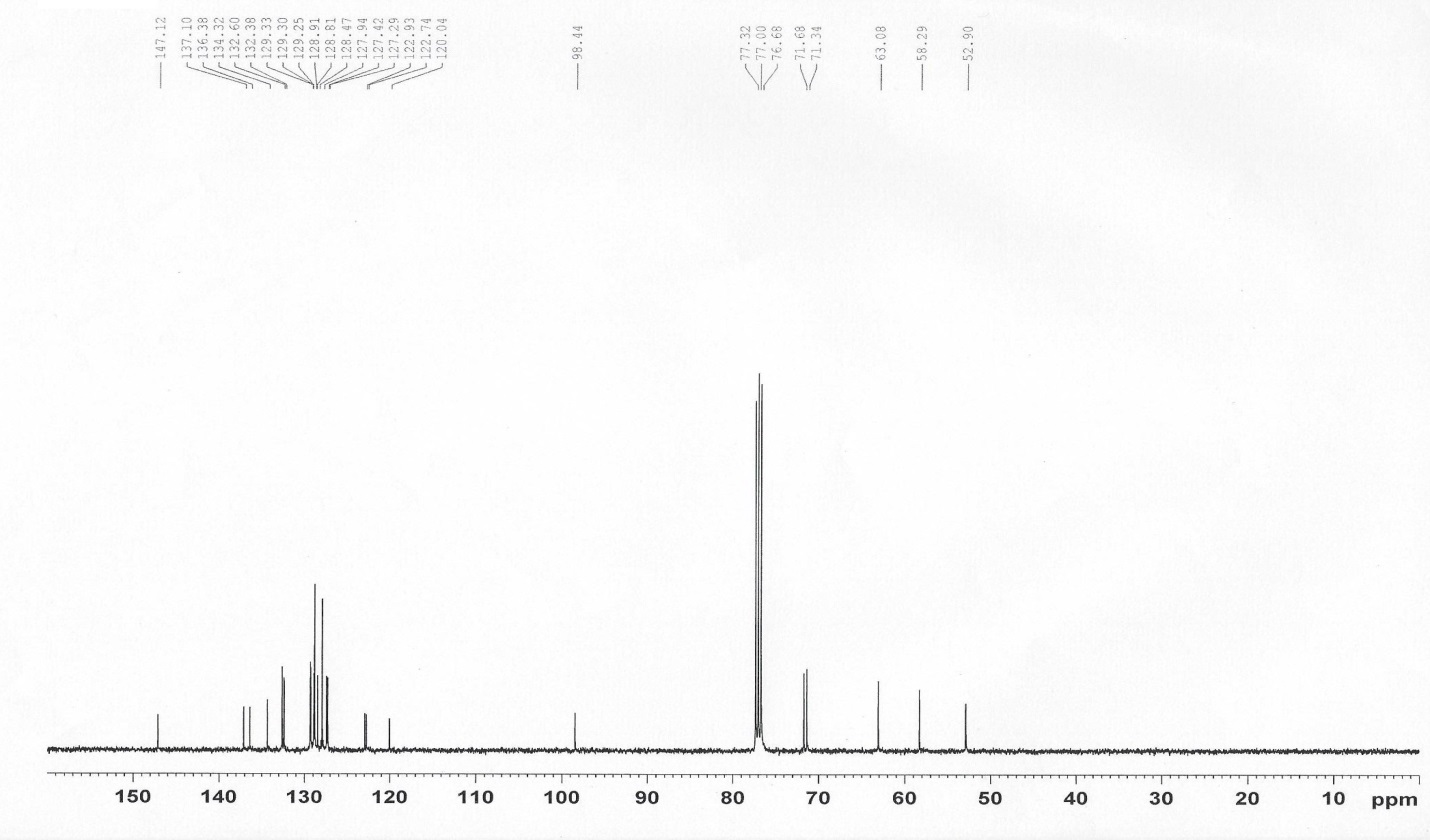


**Figure S40**. ^13^C NMR spectrum of **3ag** in CDCl_3_


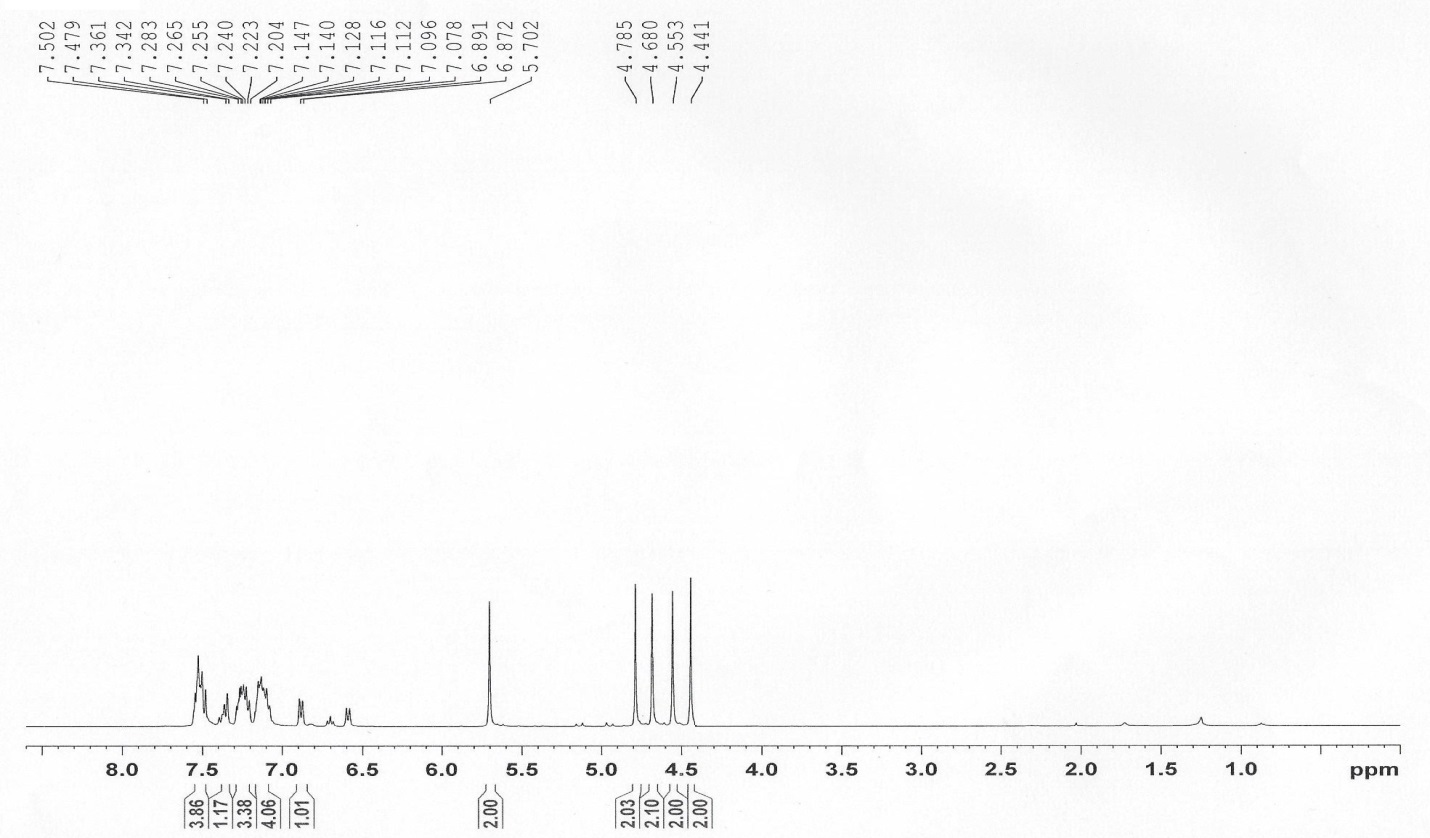


**Figure S41**. ^1^H NMR spectrum of **3bg** in CDCl_3_


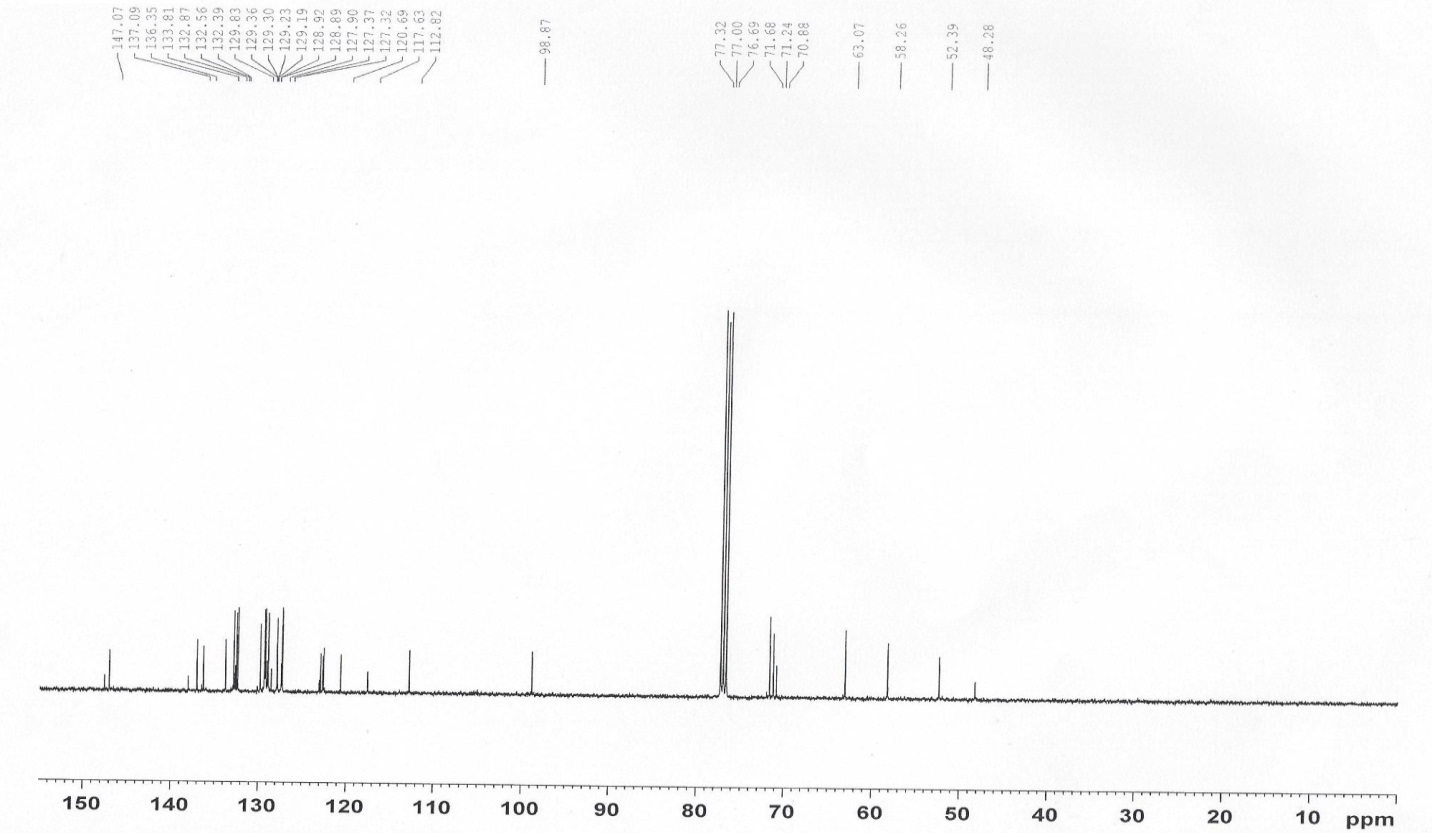


**Figure S42**. ^13^C NMR spectrum of **3bg** in CDCl_3_


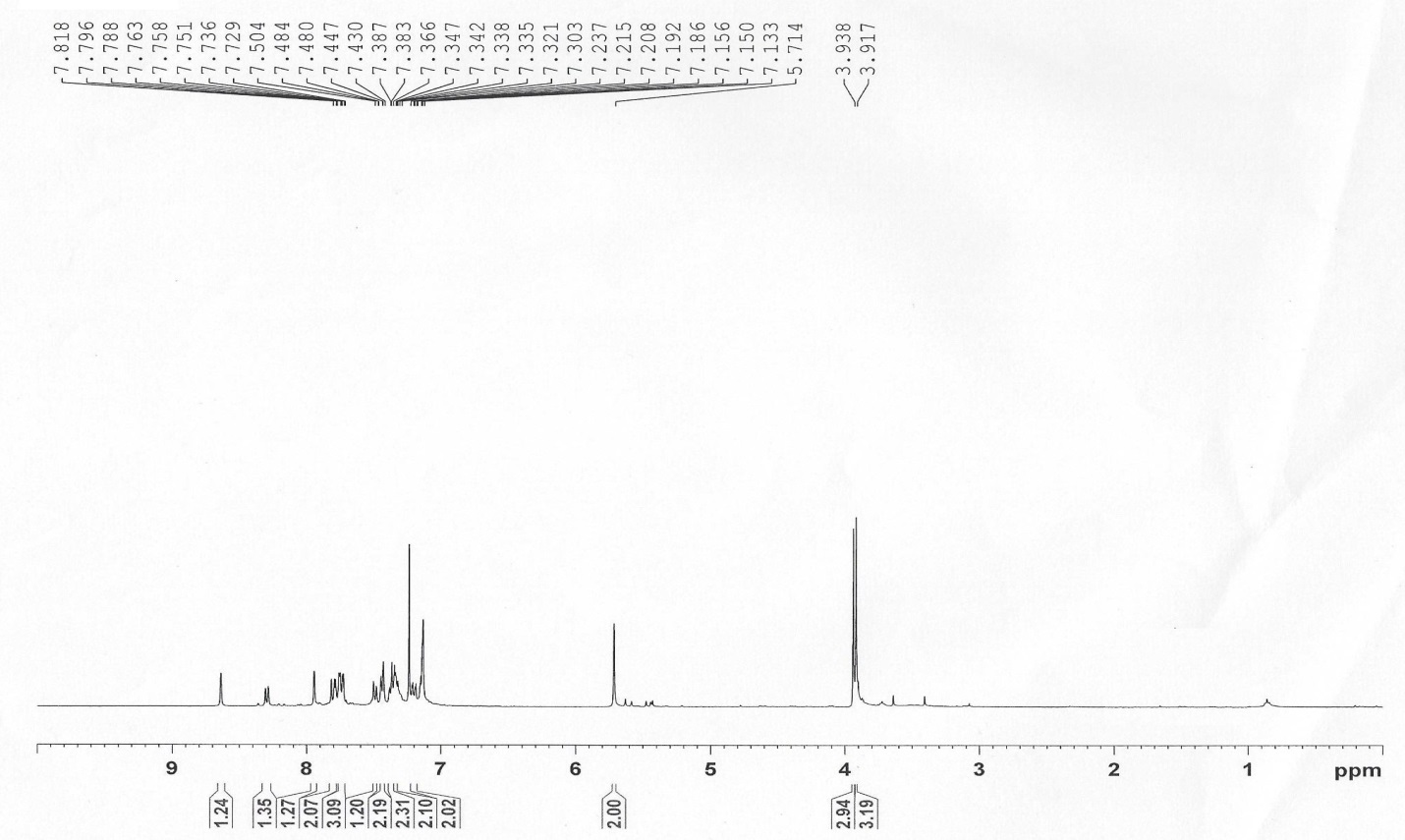


**Figure S43**. ^1^H NMR spectrum of **3ah** in CDCl_3_


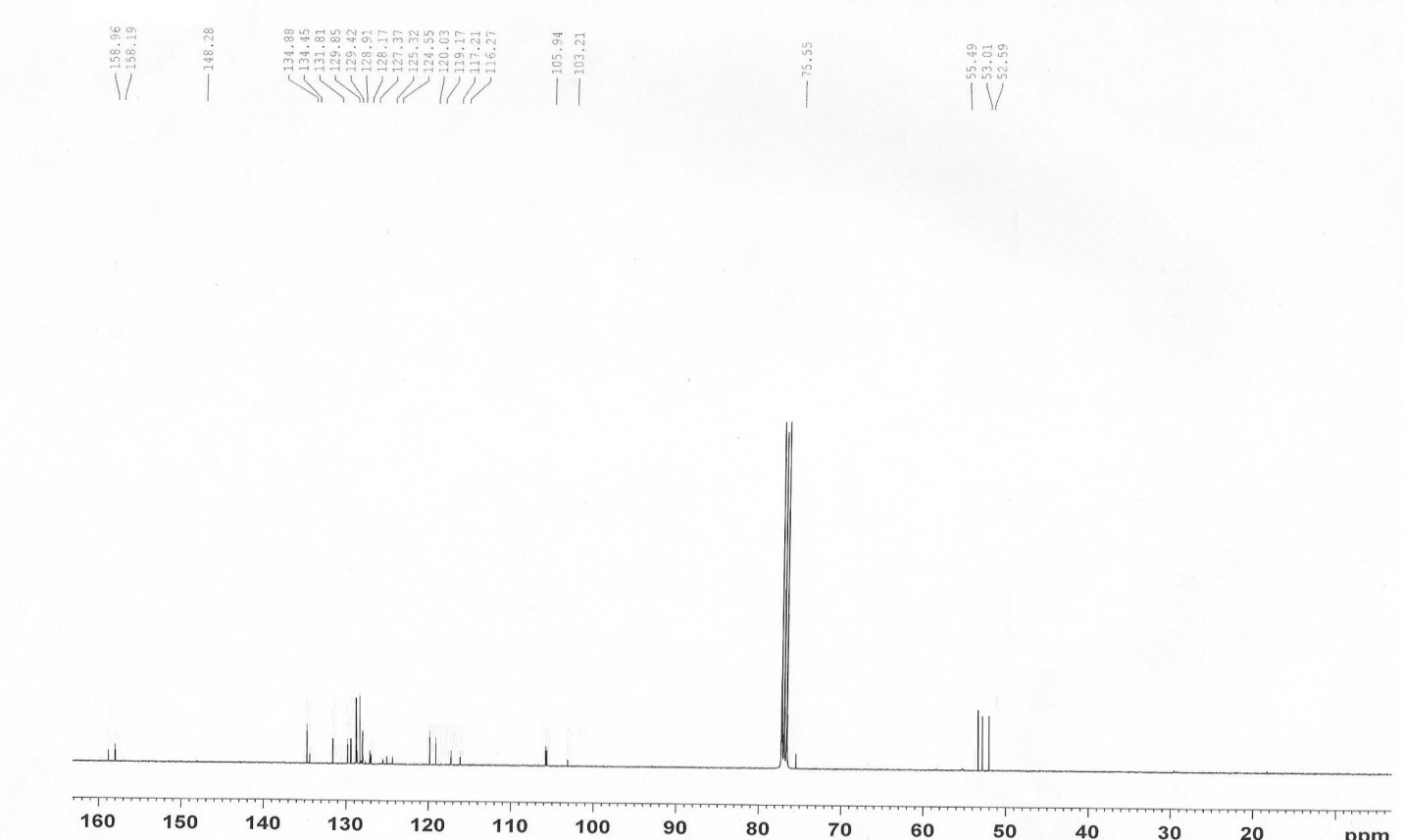


**Figure S44**. ^13^C NMR spectrum of **3ah** in CDCl_3_


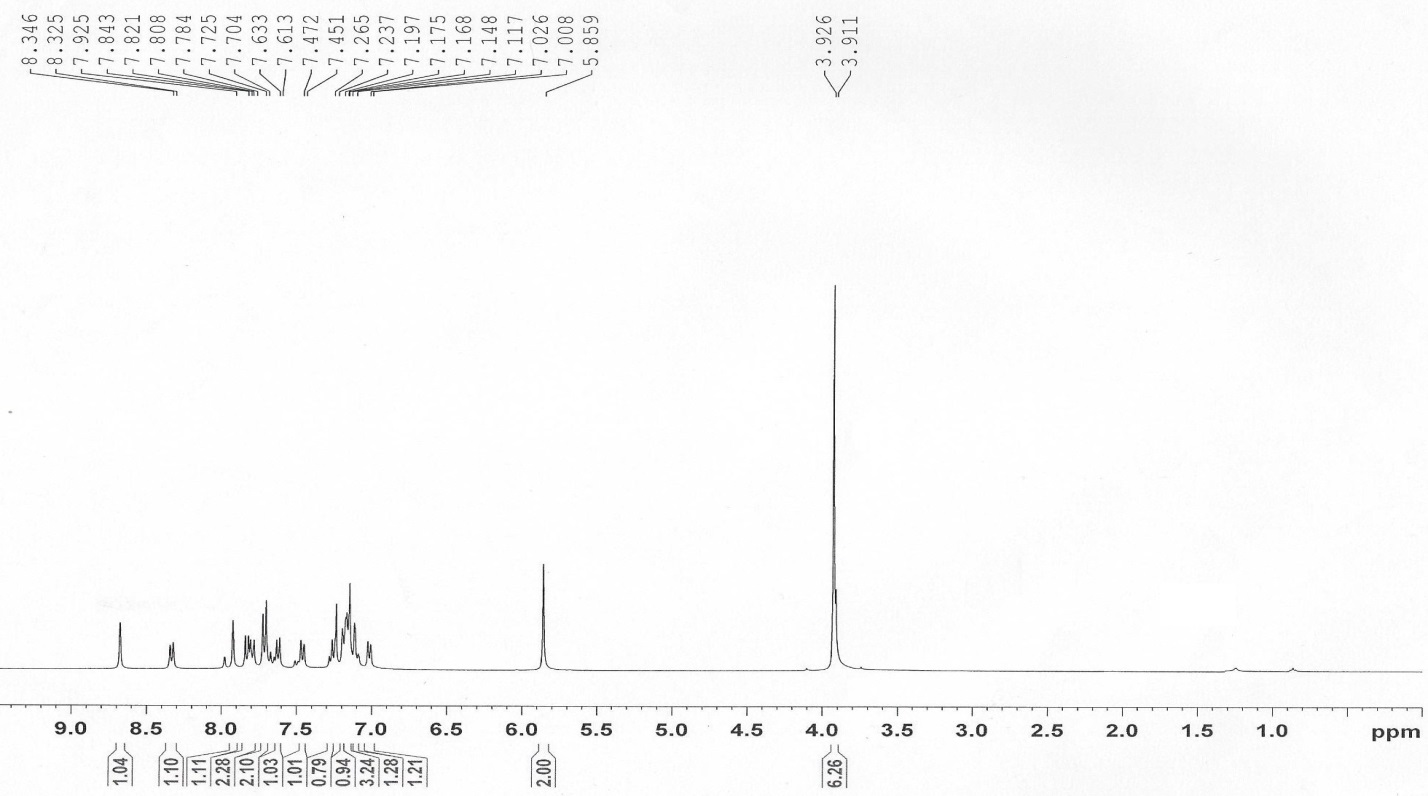


**Figure S45**. ^1^H NMR spectrum of **3bh** in CDCl_3_


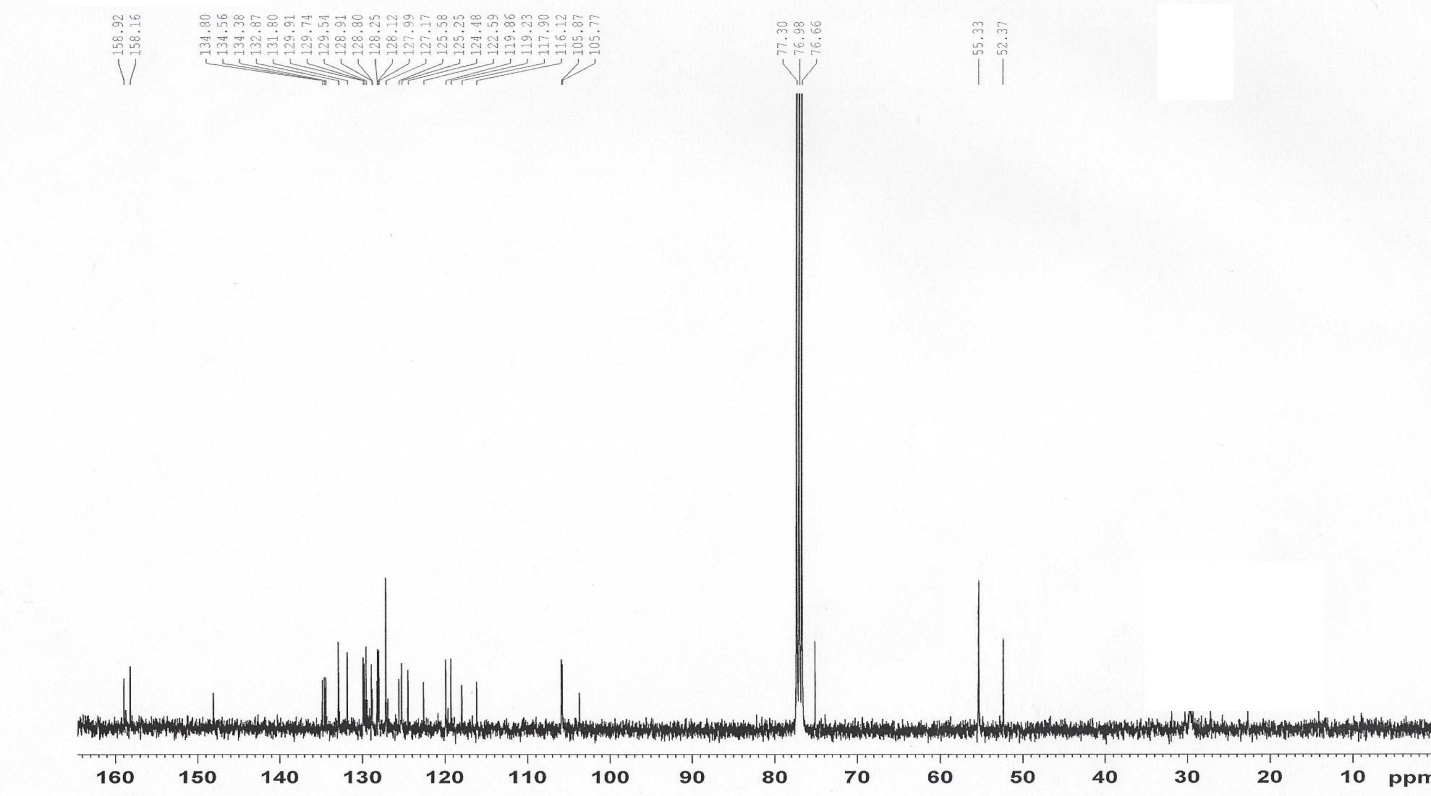


**Figure S46**. ^13^C NMR spectrum of **3bh** in CDCl_3_


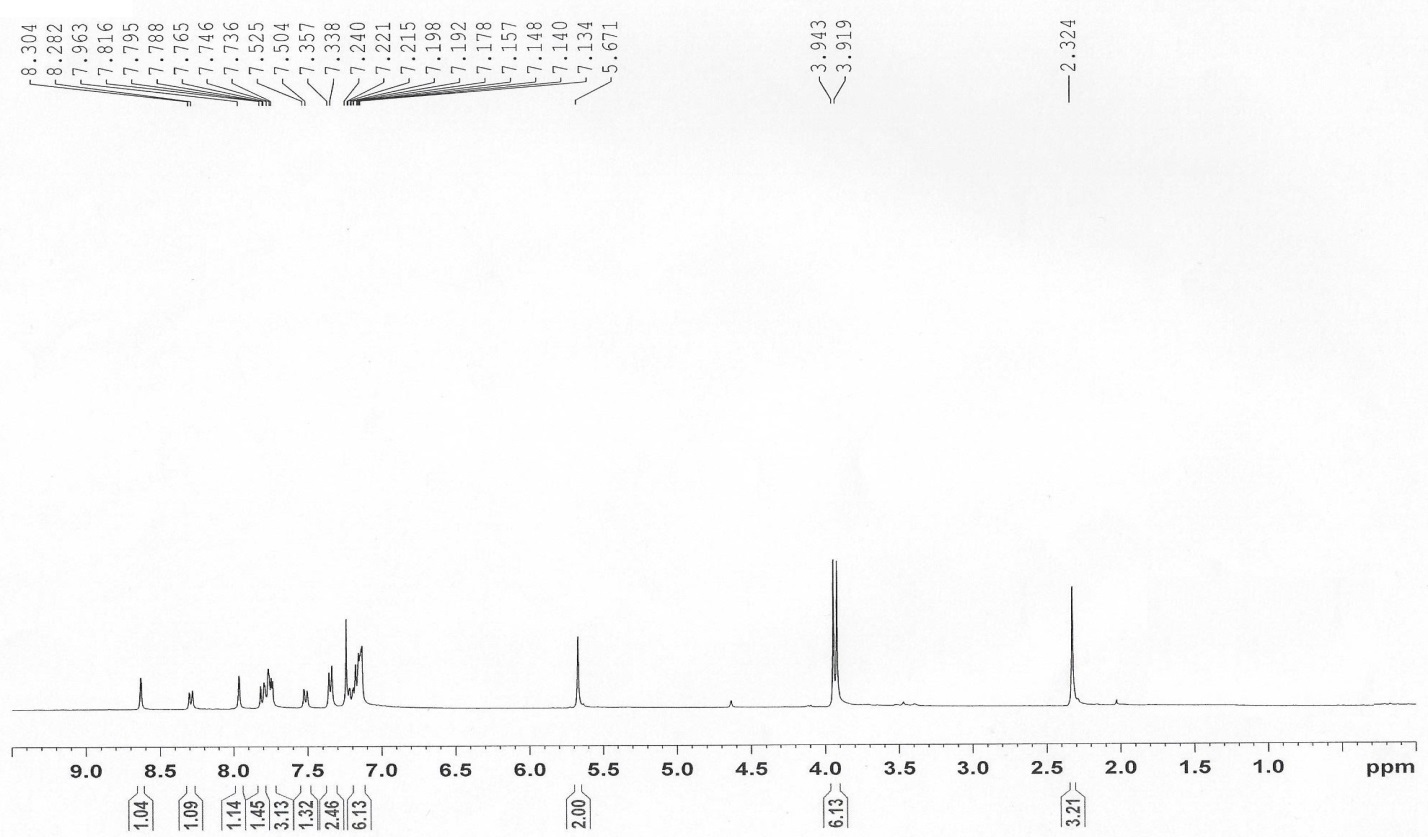


**Figure S47**. ^1^H NMR spectrum of **3ch** in CDCl_3_


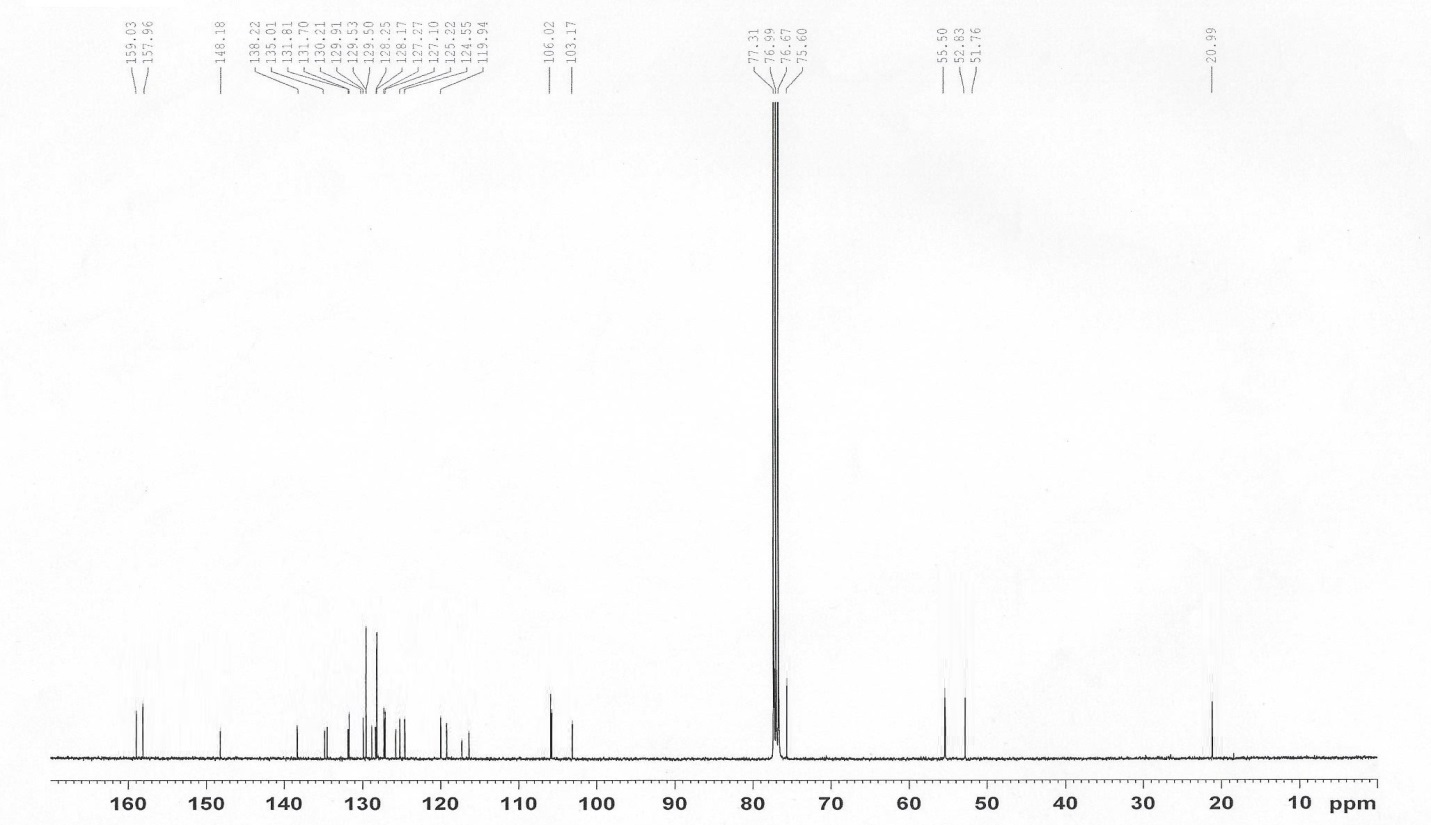


**Figure S48**. ^13^C NMR spectrum of **3ch** in CDCl_3_


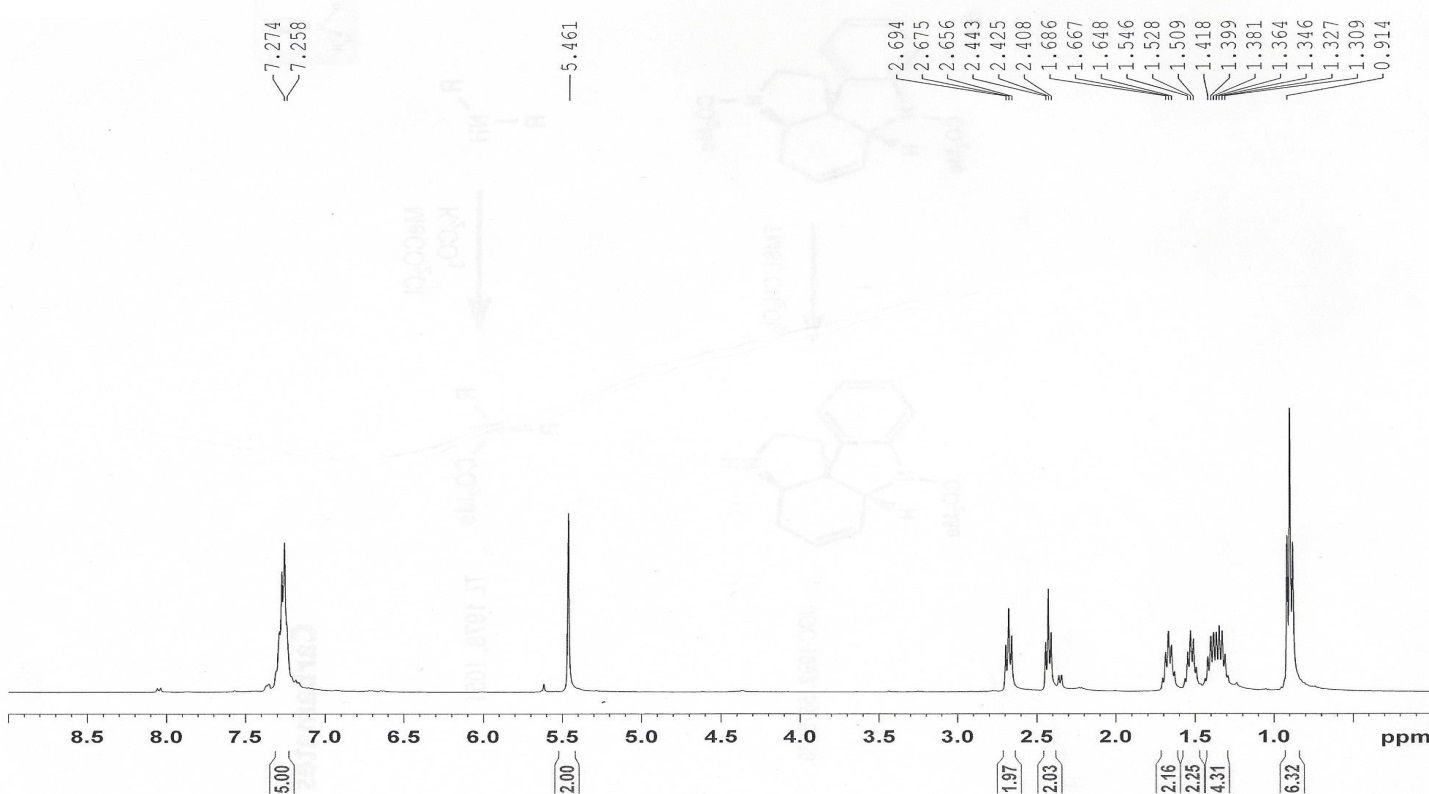


**Figure S49**. ^1^H NMR spectrum of **3ch** in CDCl_3_


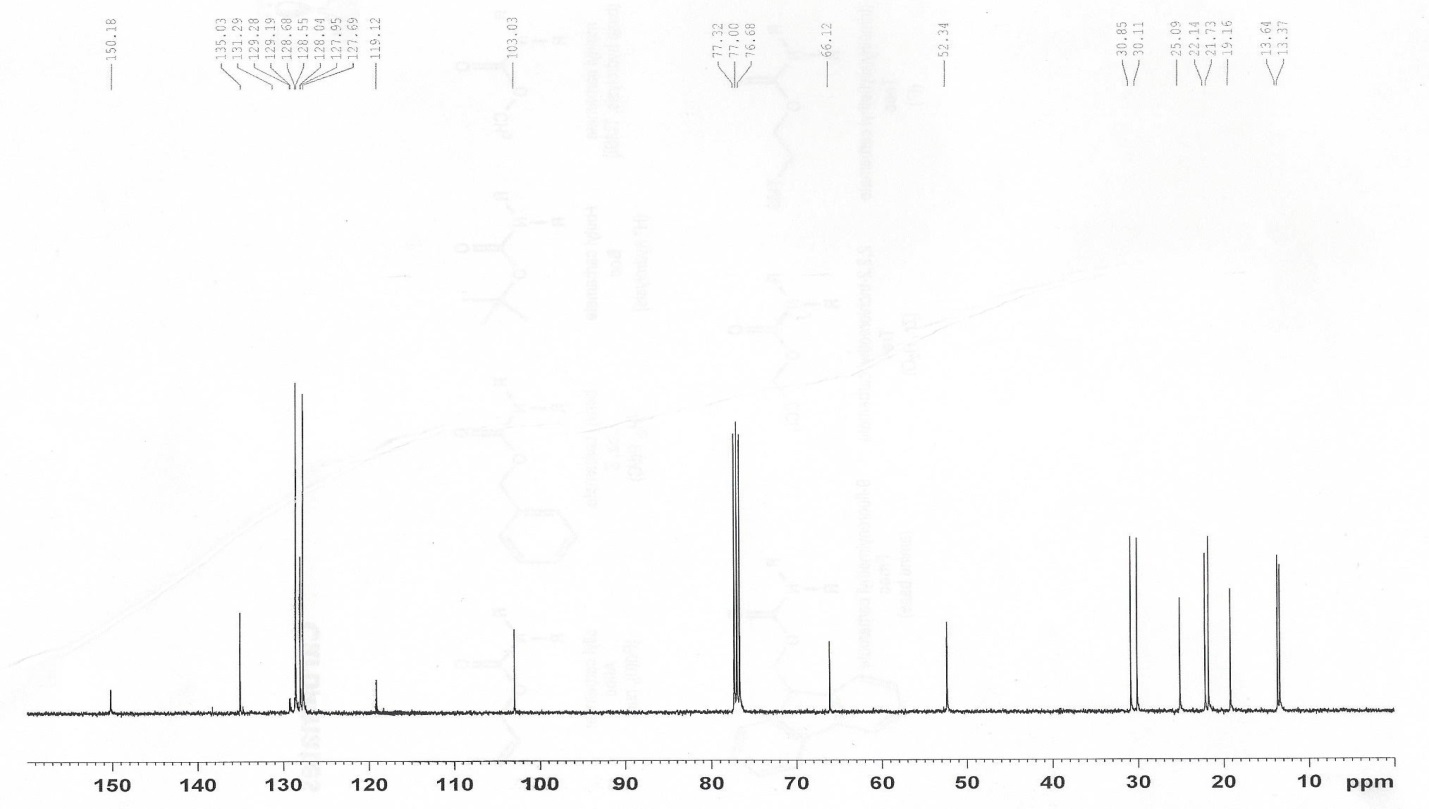


**Figure S50**. ^13^C NMR spectrum of **3ch** in CDCl_3_


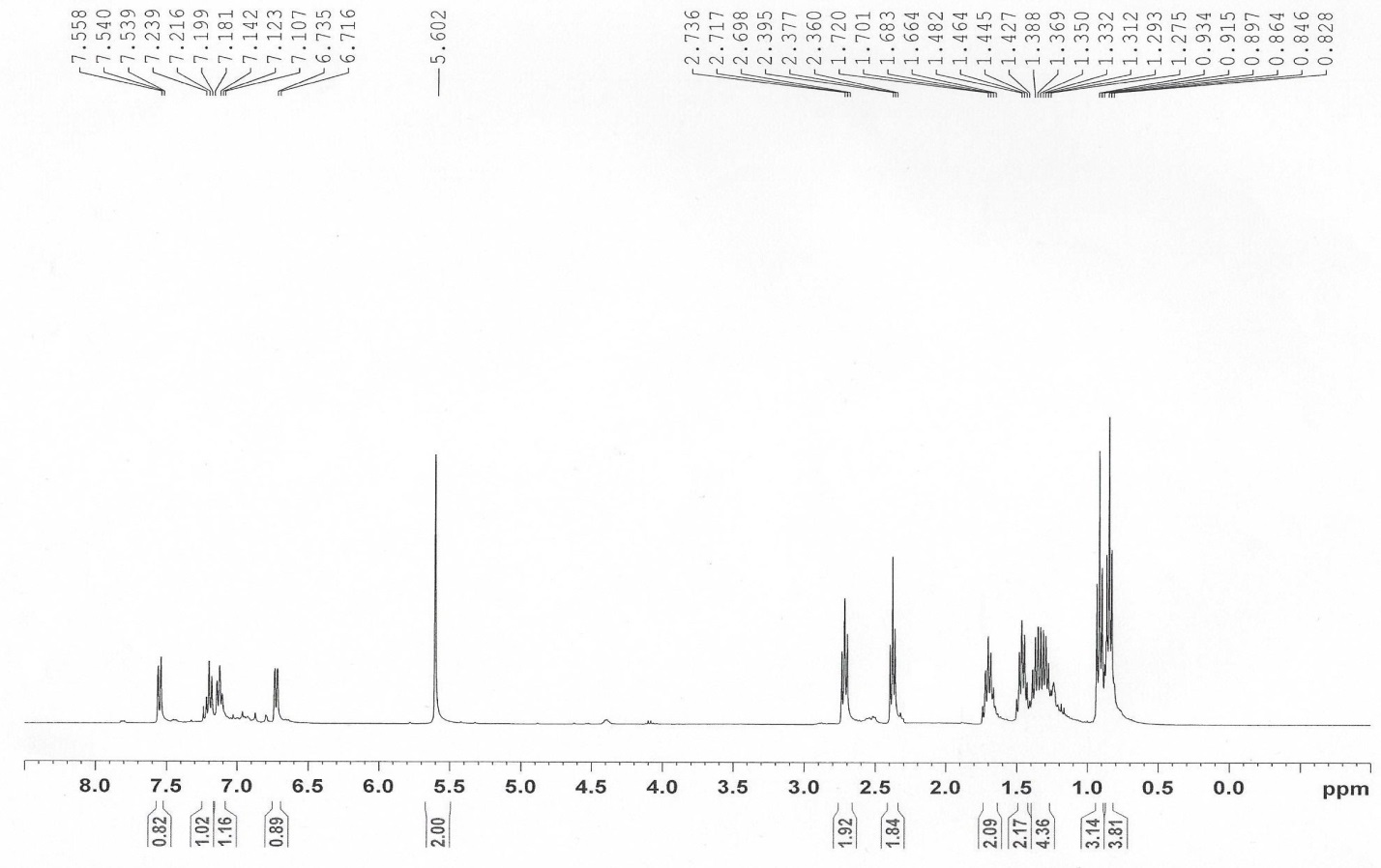


**Figure S51**. ^1^H NMR spectrum of **3bi** in CDCl_3_


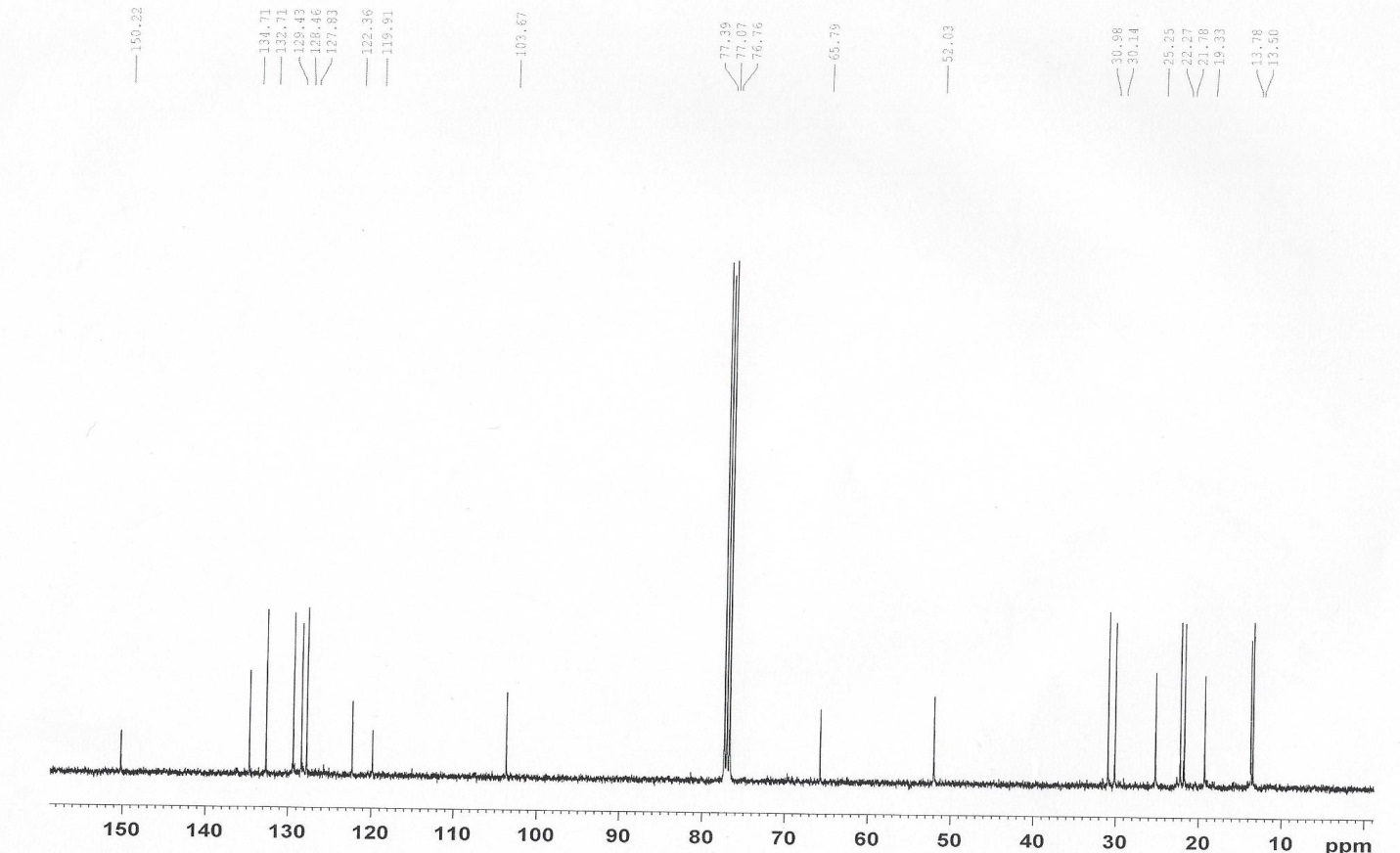


**Figure S52**. ^13^C NMR spectrum of **3ch** in CDCl_3_


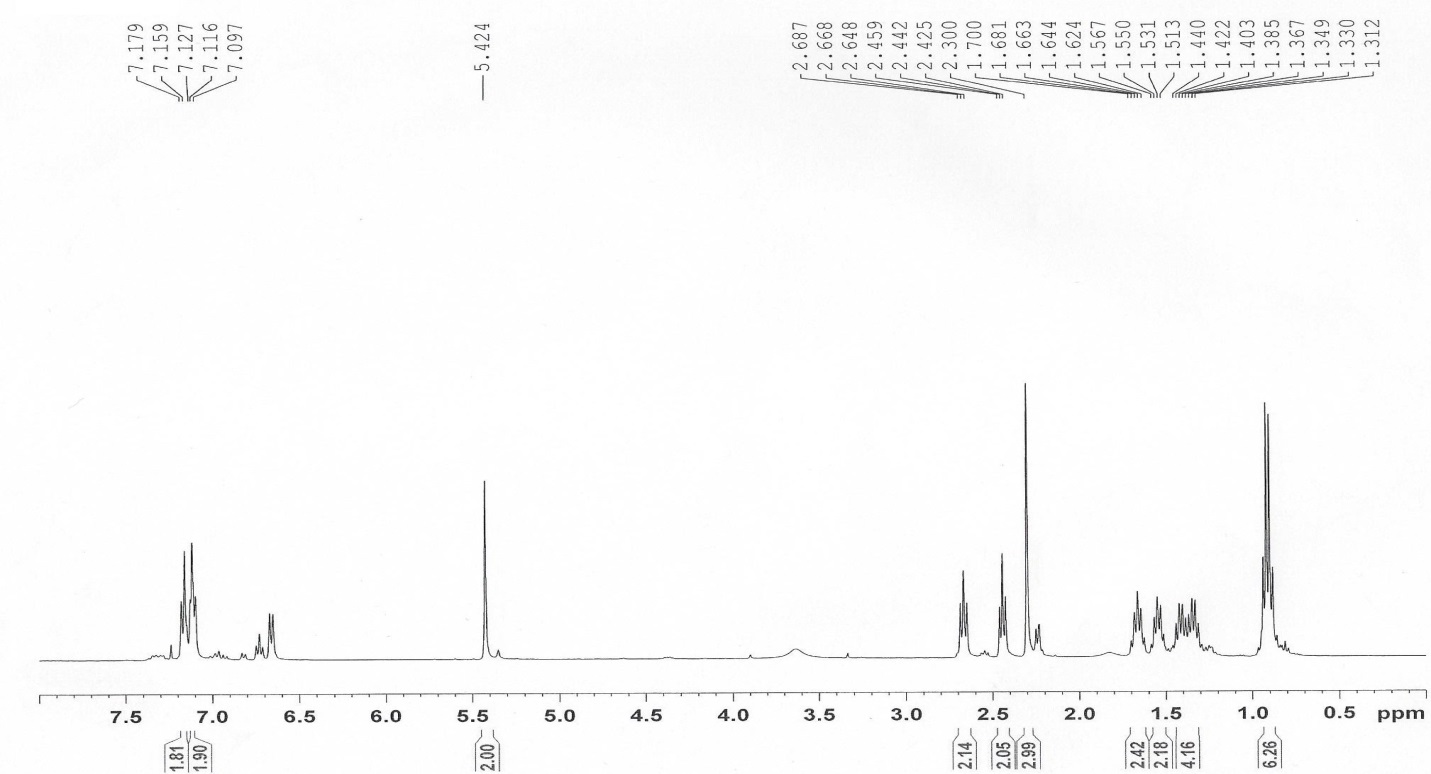


**Figure S53**. ^1^H NMR spectrum of **3ci** in CDCl_3_


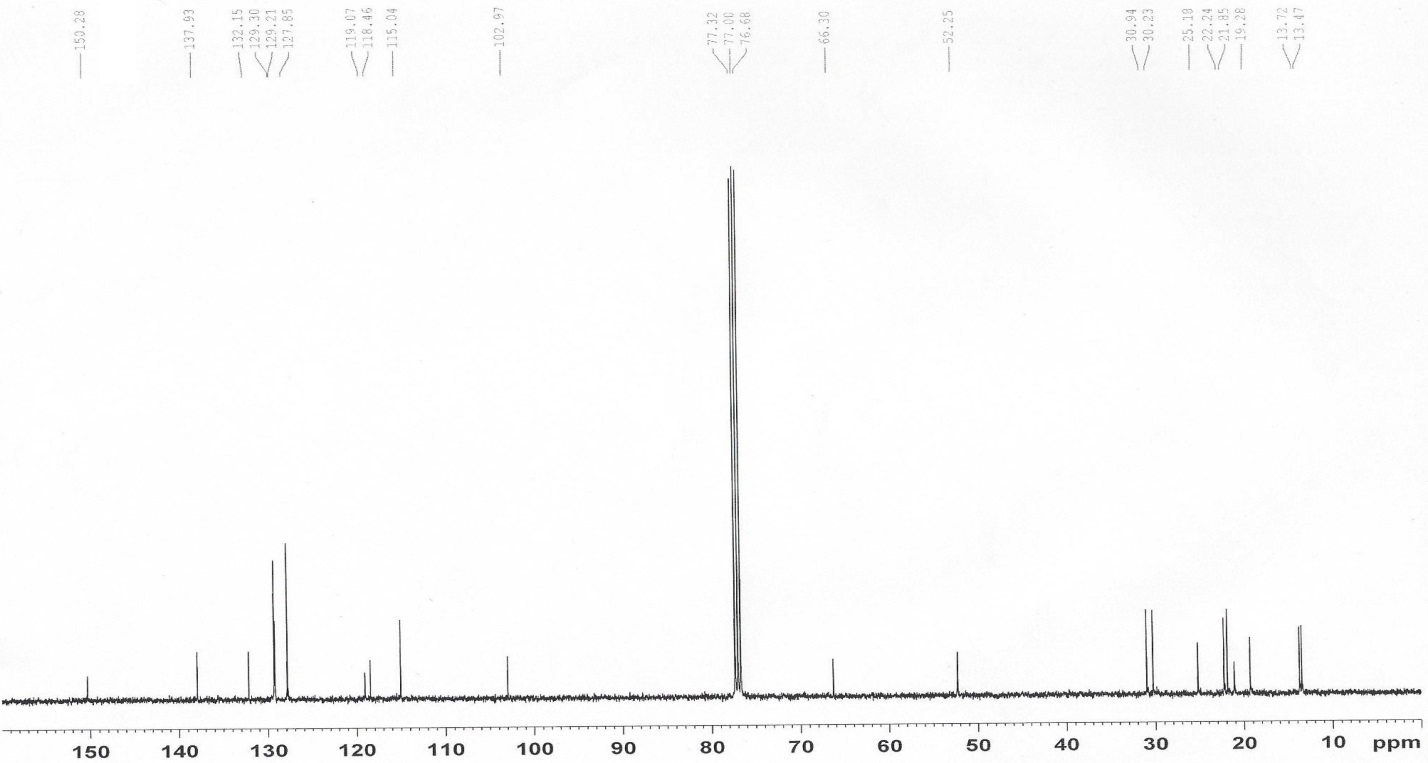


**Figure S54**. ^13^C NMR spectrum of **3ci** in CDCl_3_


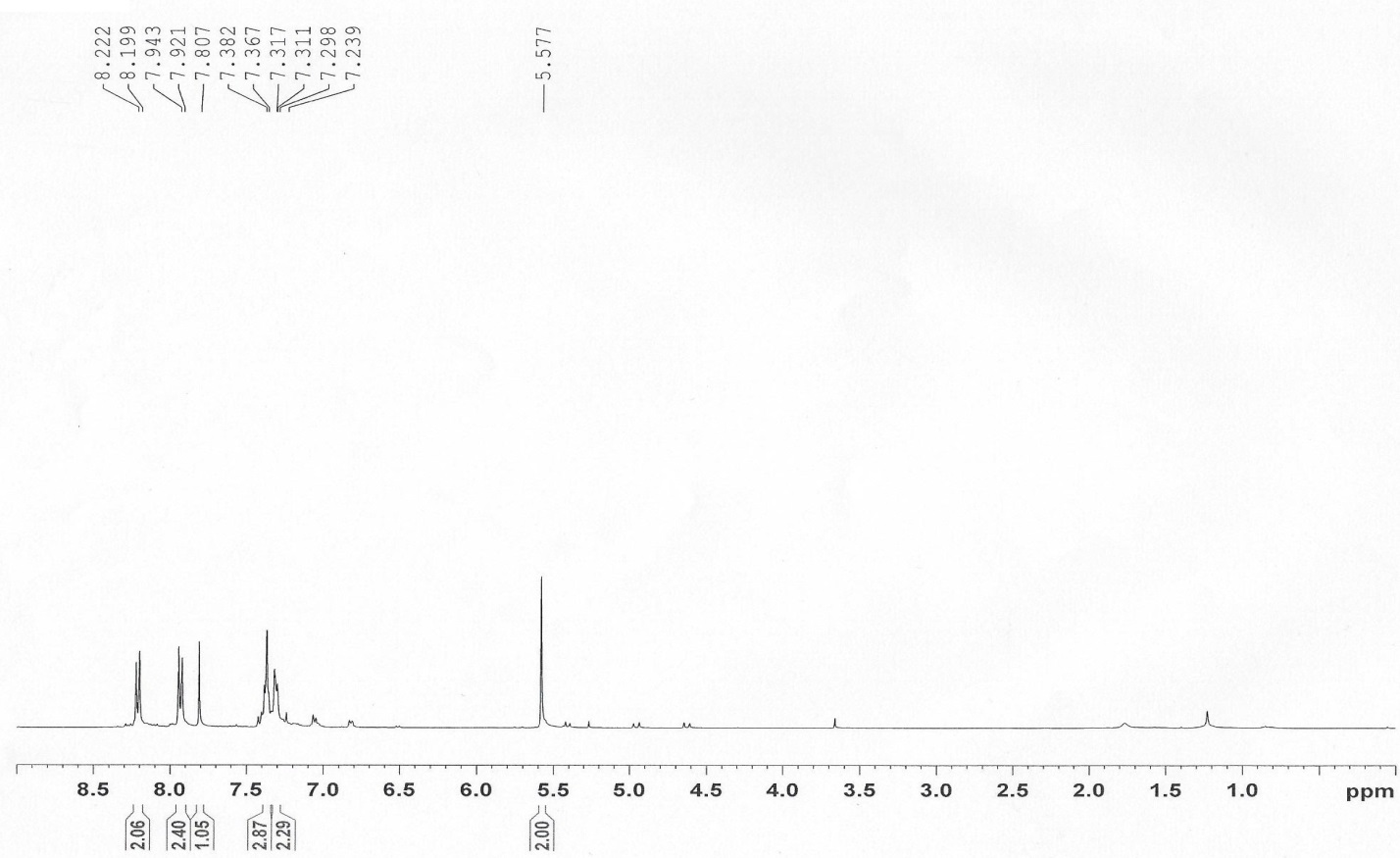


**Figure S55**. ^1^H NMR spectrum of **4aj** in CDCl_3_


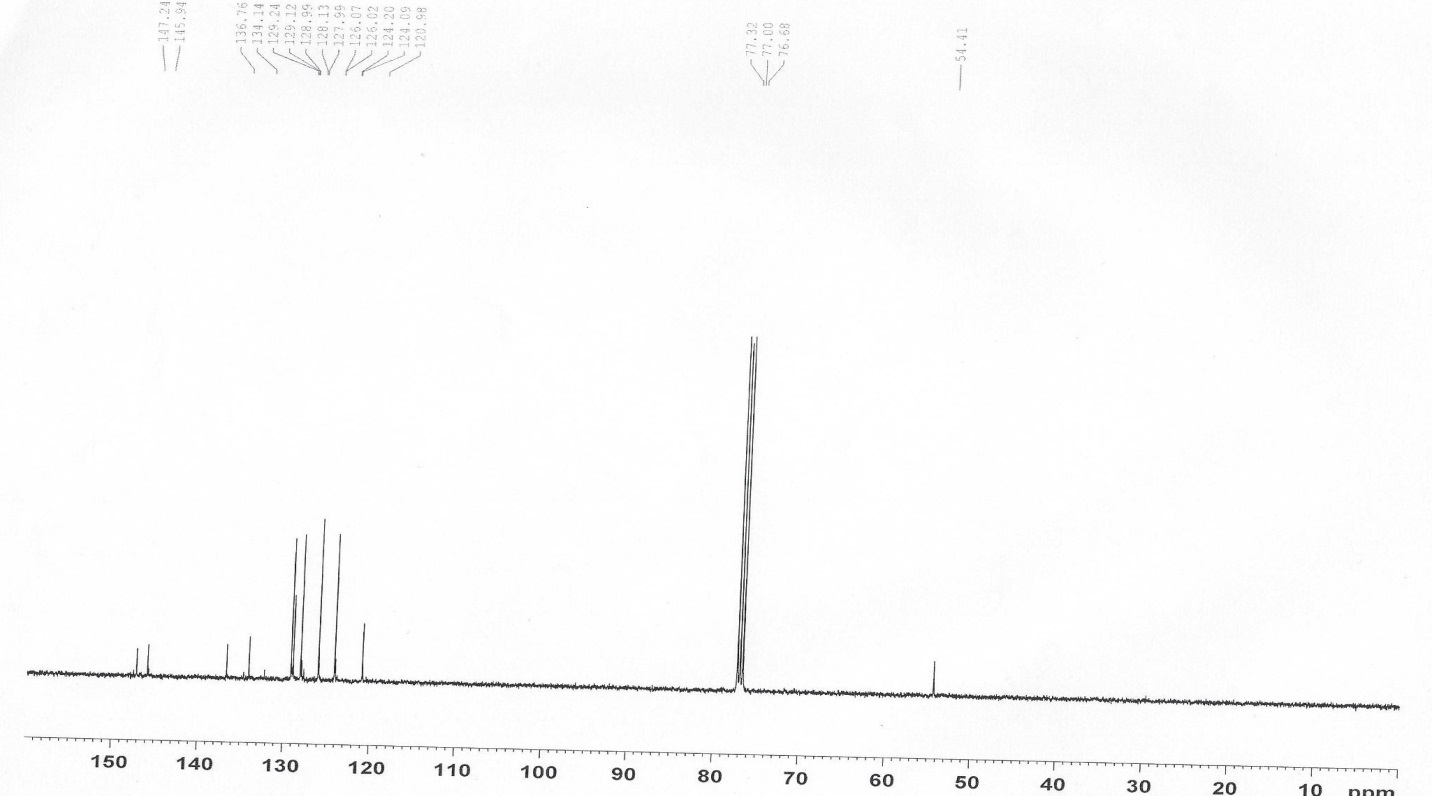


**Figure S56**. ^13^C NMR spectrum of **3ci** in CDCl_3_


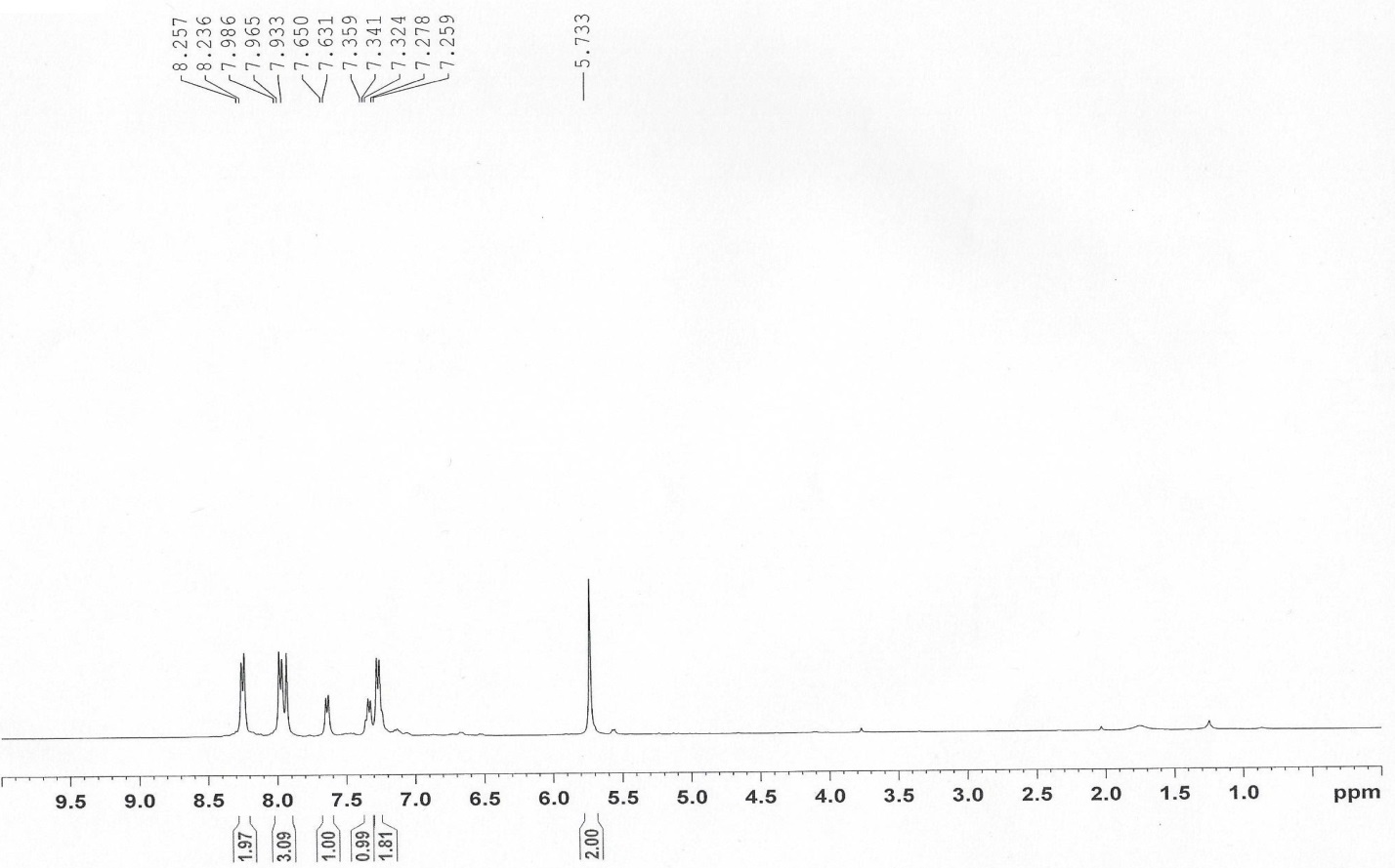


**Figure S57**. ^1^H NMR spectrum of **4bj** in CDCl_3_


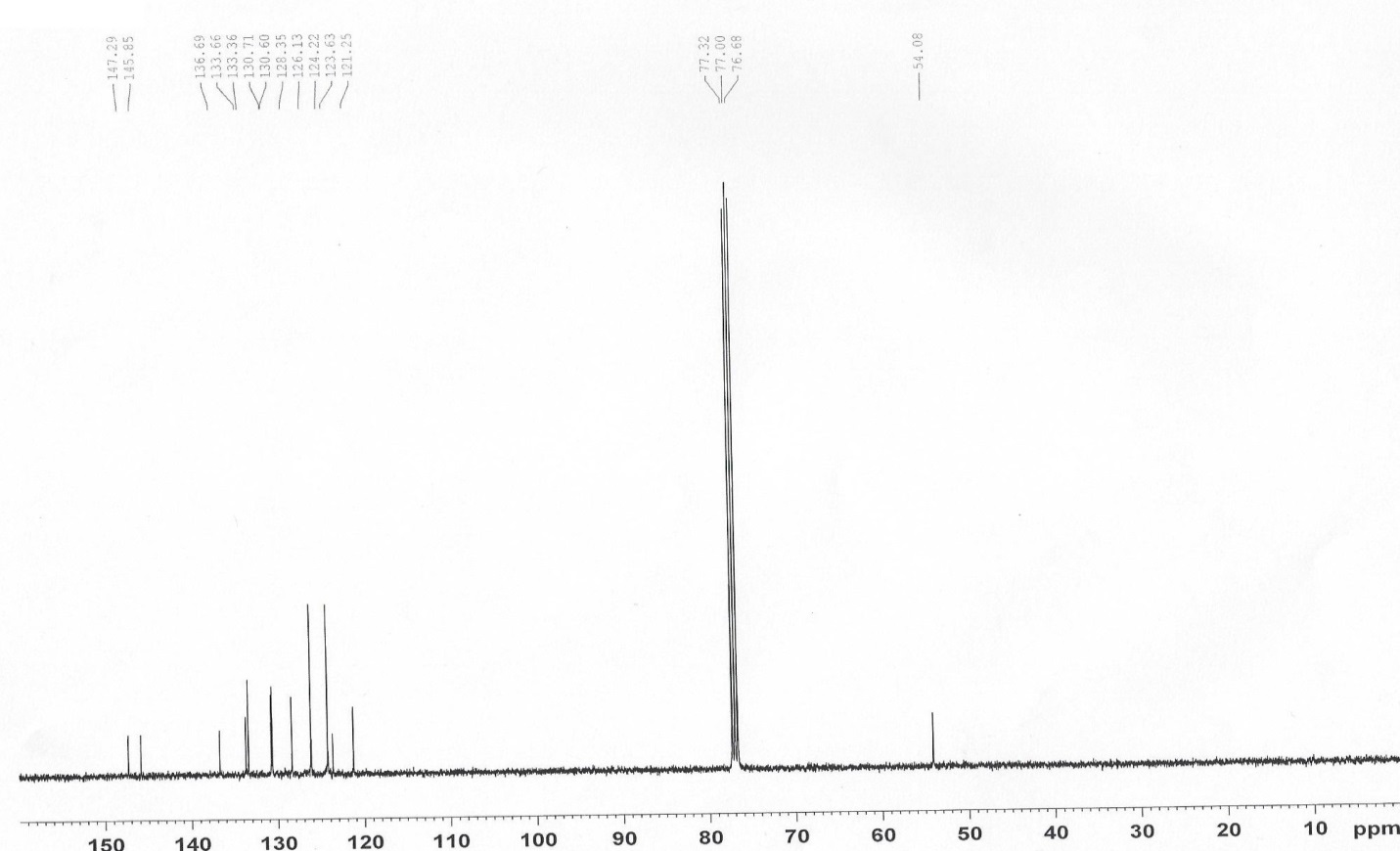


**Figure S58**. ^13^C NMR spectrum of **4bj** in CDCl_3_


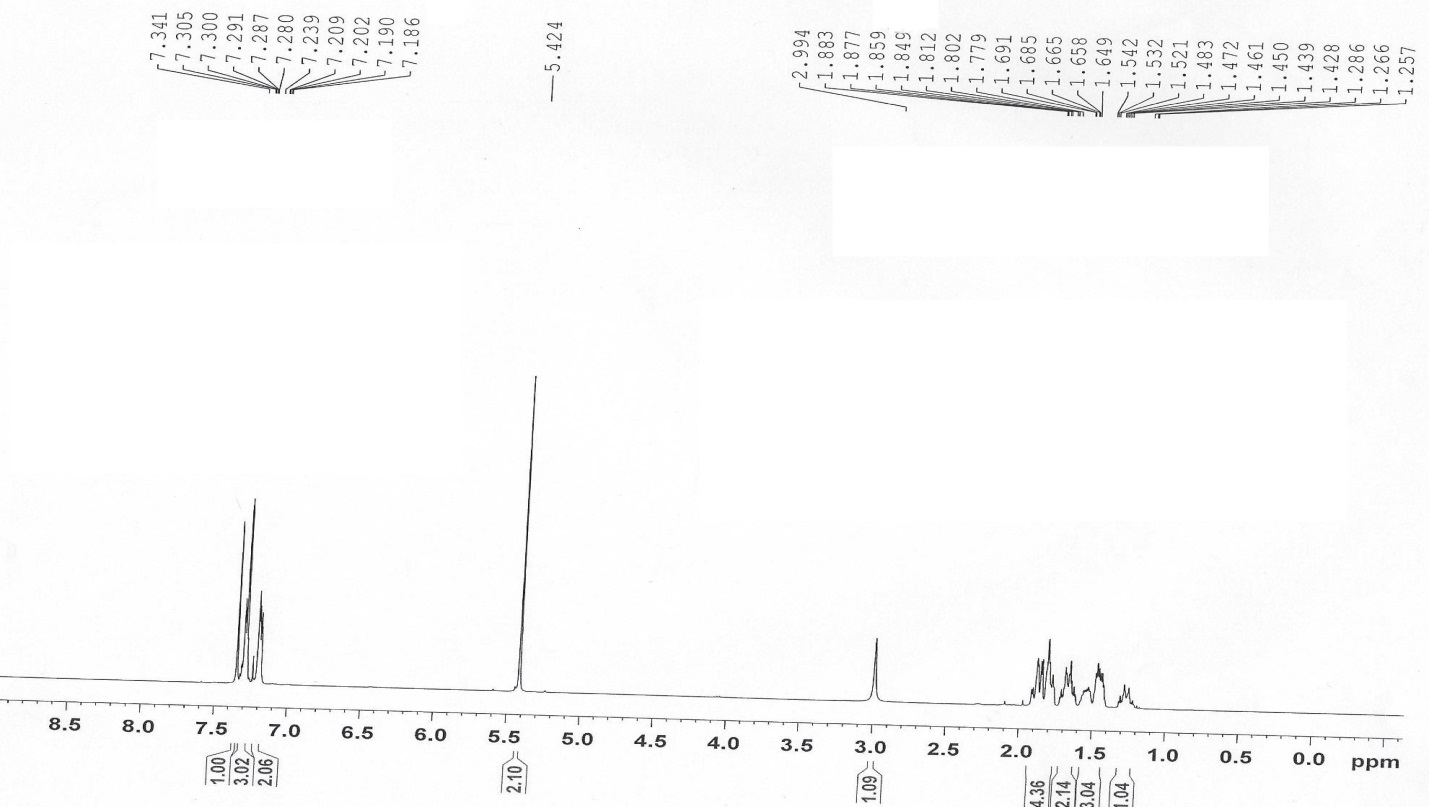


**Figure S59**. ^1^H NMR spectrum of **4ak** in CDCl_3_


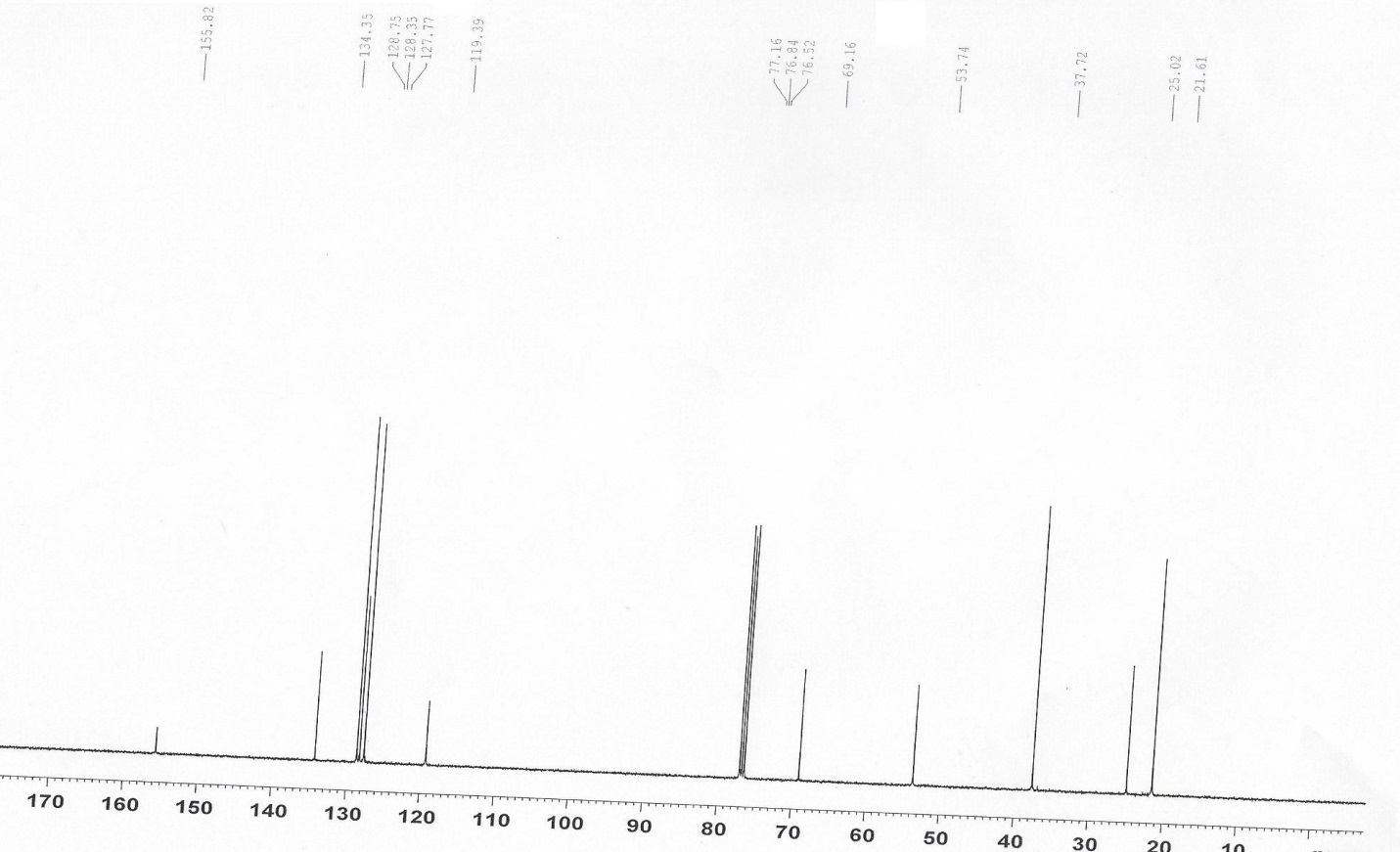


**Figure S60**. ^13^C NMR spectrum of **4ak** in CDCl_3_


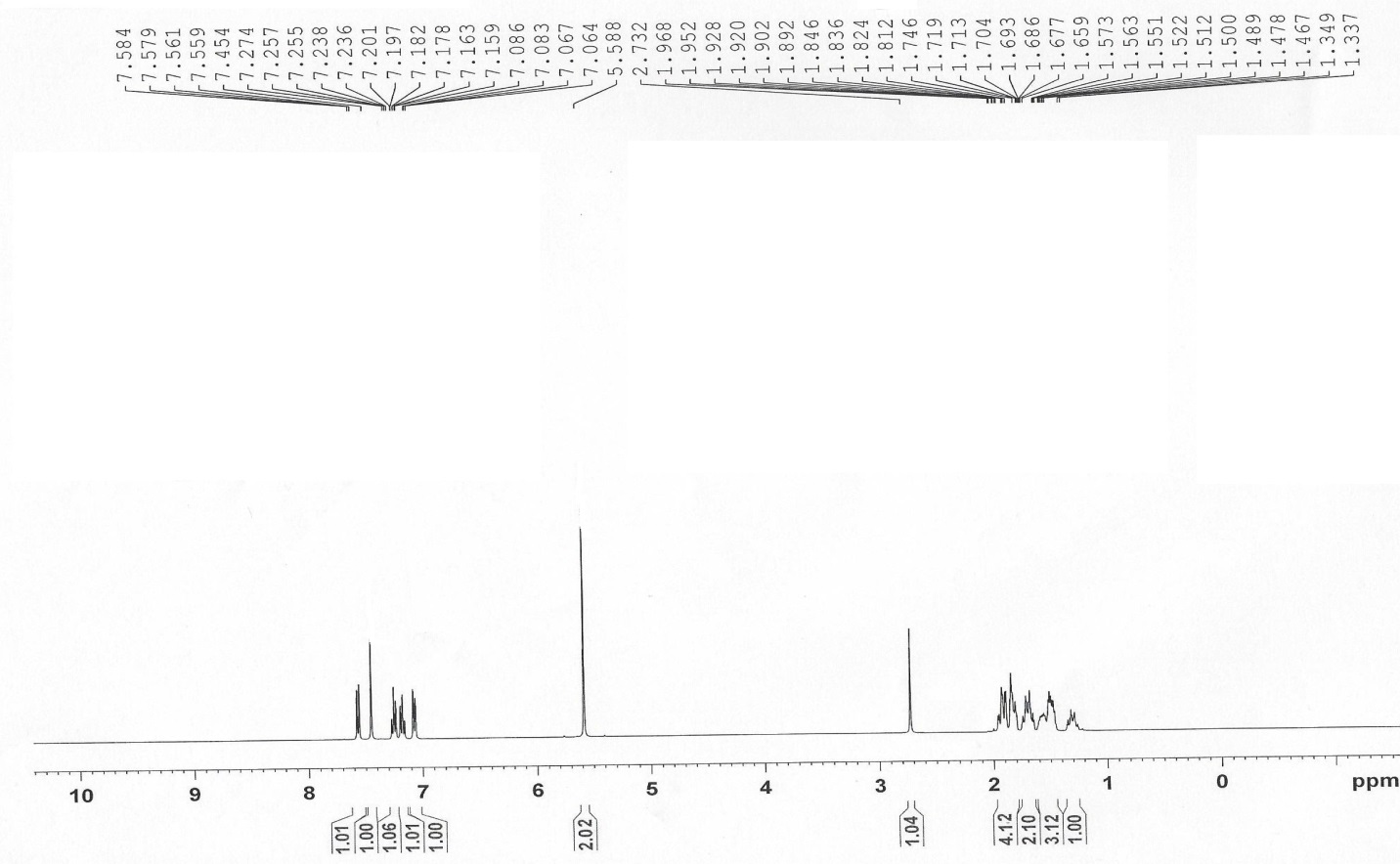


**Figure S61**. ^1^H NMR spectrum of **4bk** in CDCl_3_


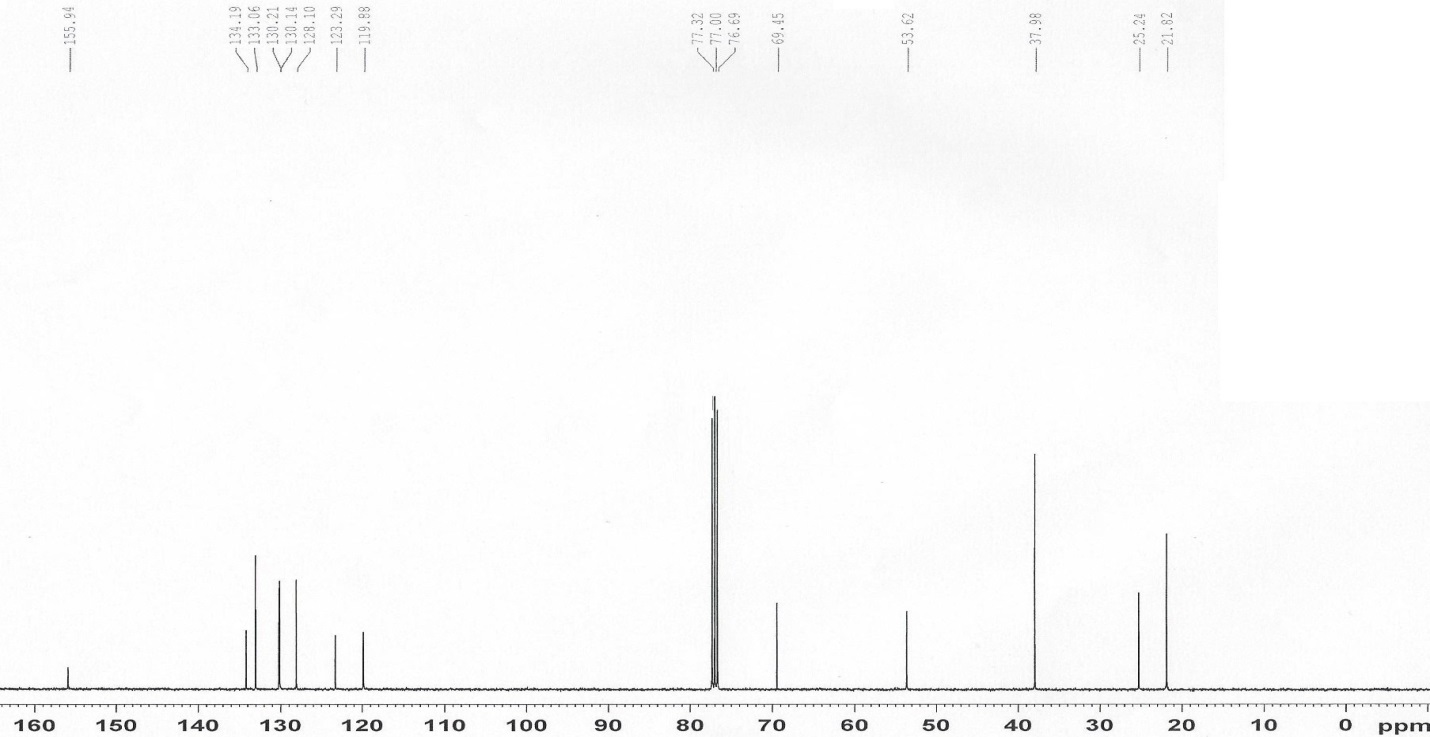


**Figure S62**. ^13^C NMR spectrum of **4bk** in CDCl_3_


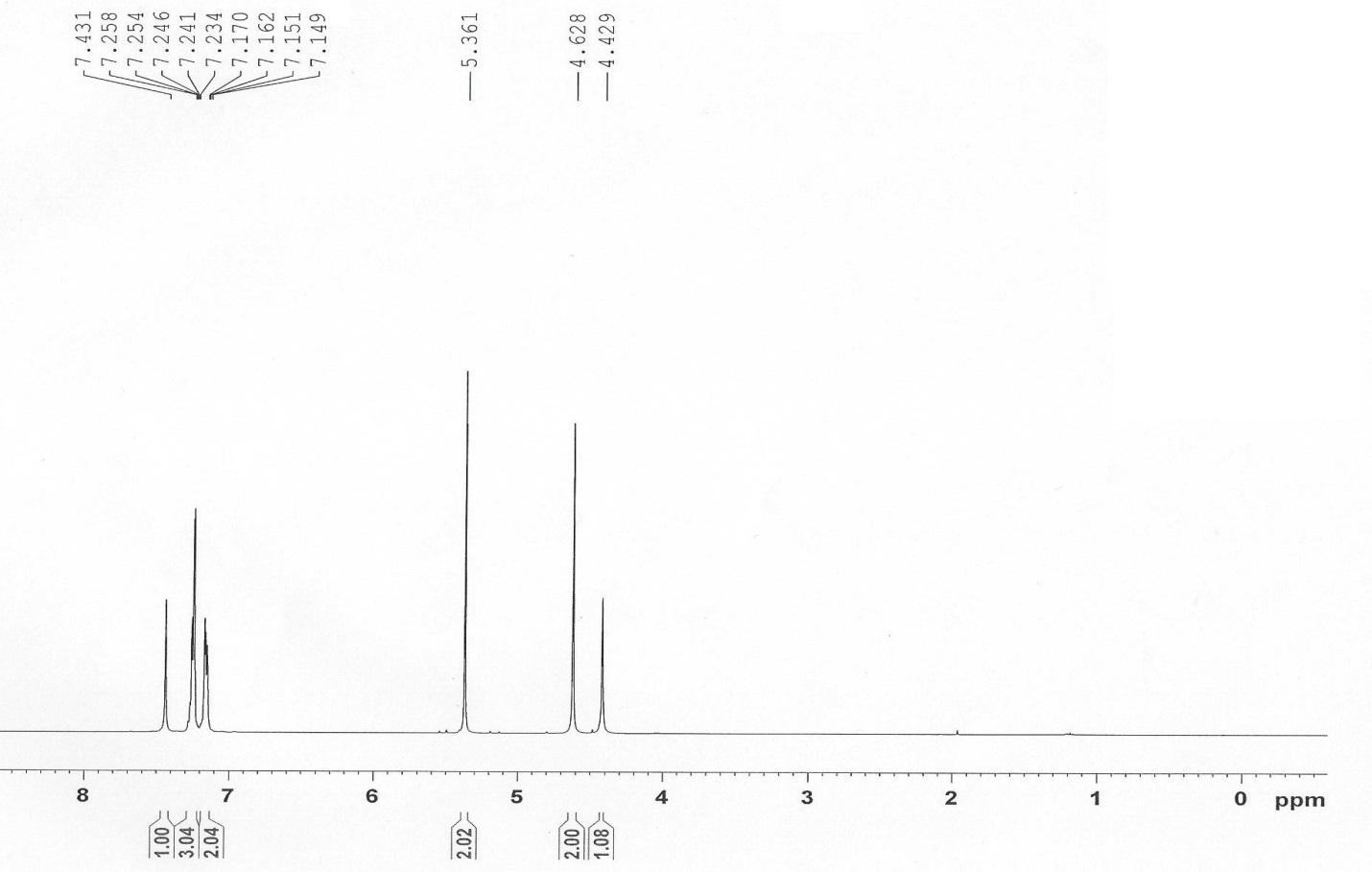


**Figure S63**. ^1^H NMR spectrum of **4al** in CDCl_3_


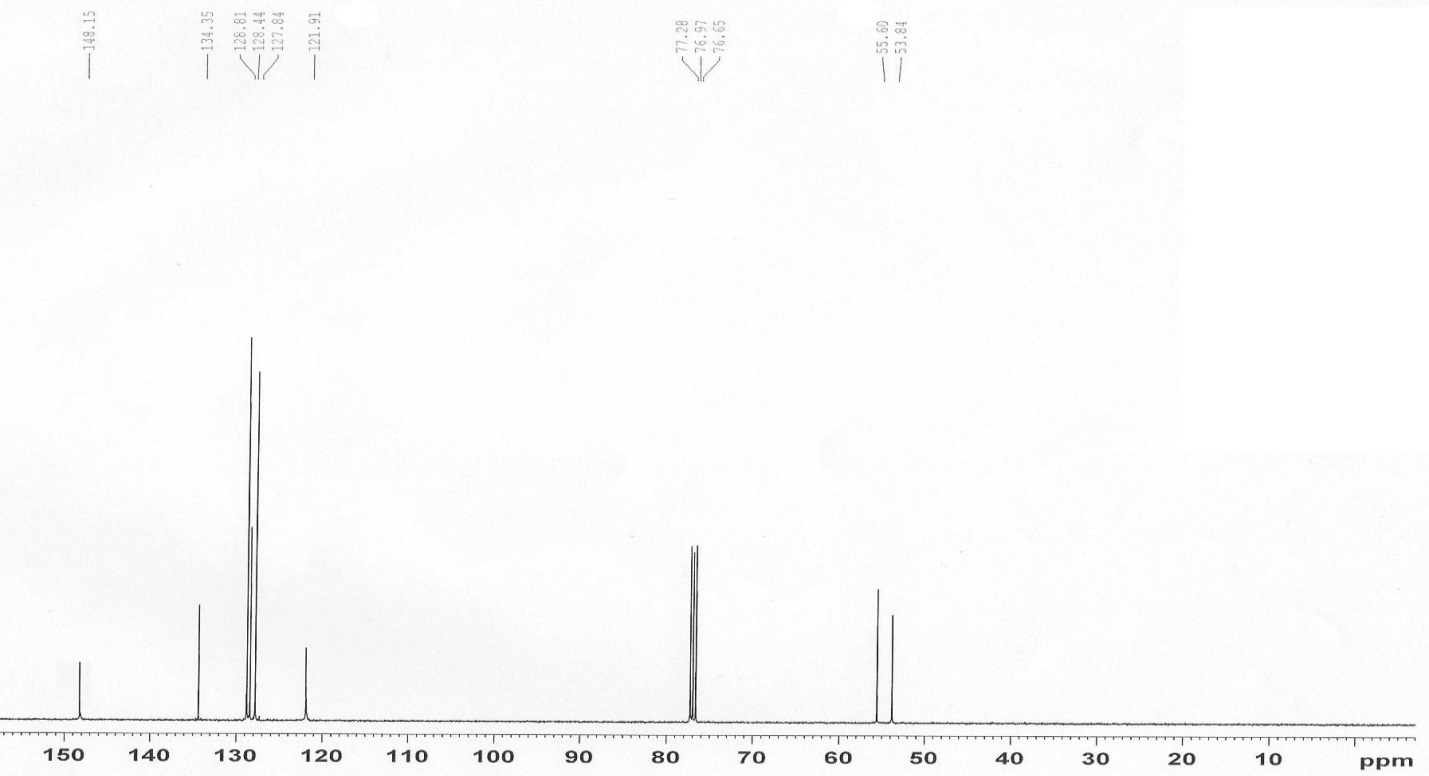


**Figure S64**. ^13^C NMR spectrum of **4al** in CDCl_3_


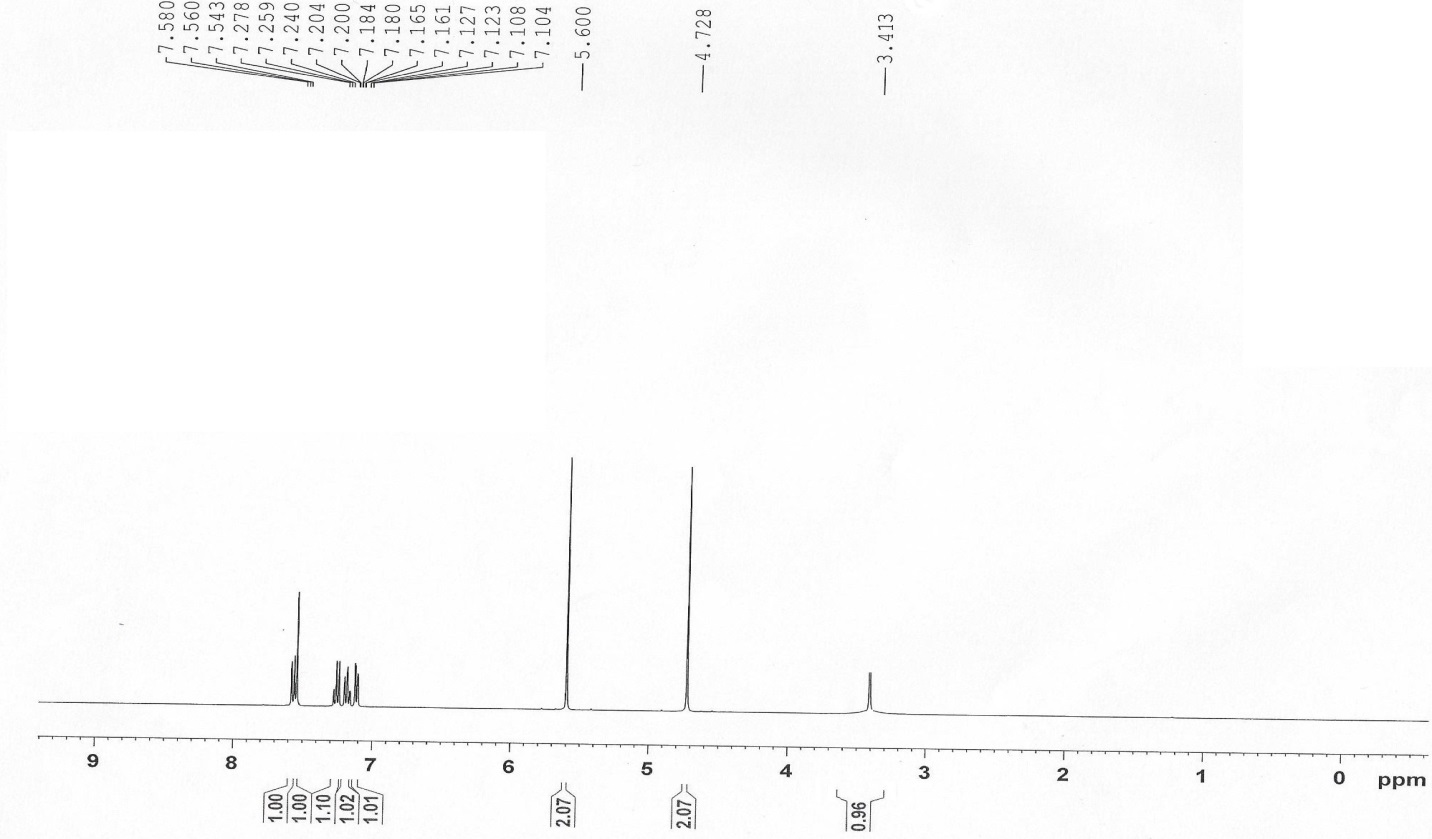


**Figure S65**. ^1^H NMR spectrum of **4bl** in CDCl_3_


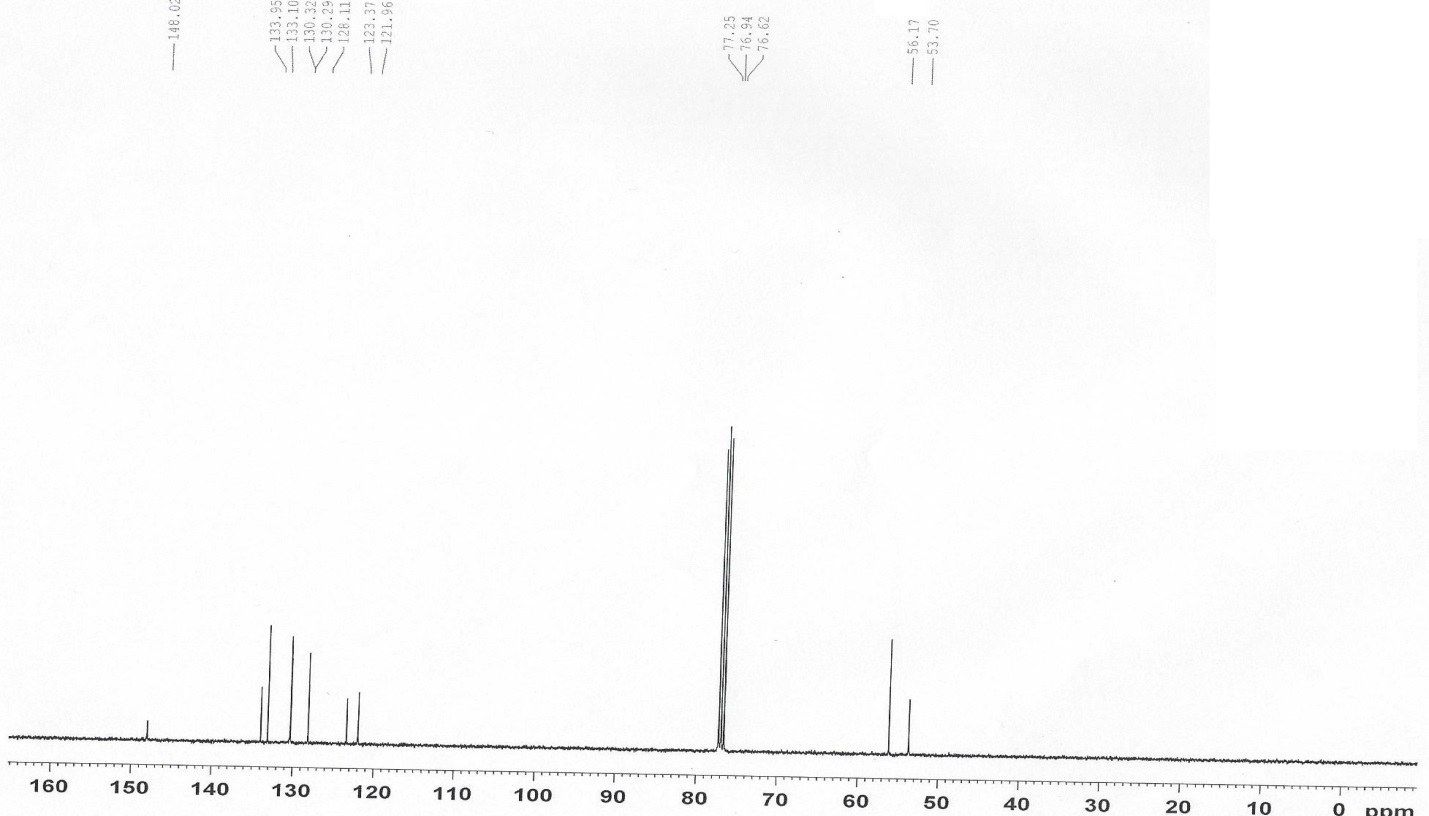


**Figure S66**. ^13^C NMR spectrum of **4bl** in CDCl_3_


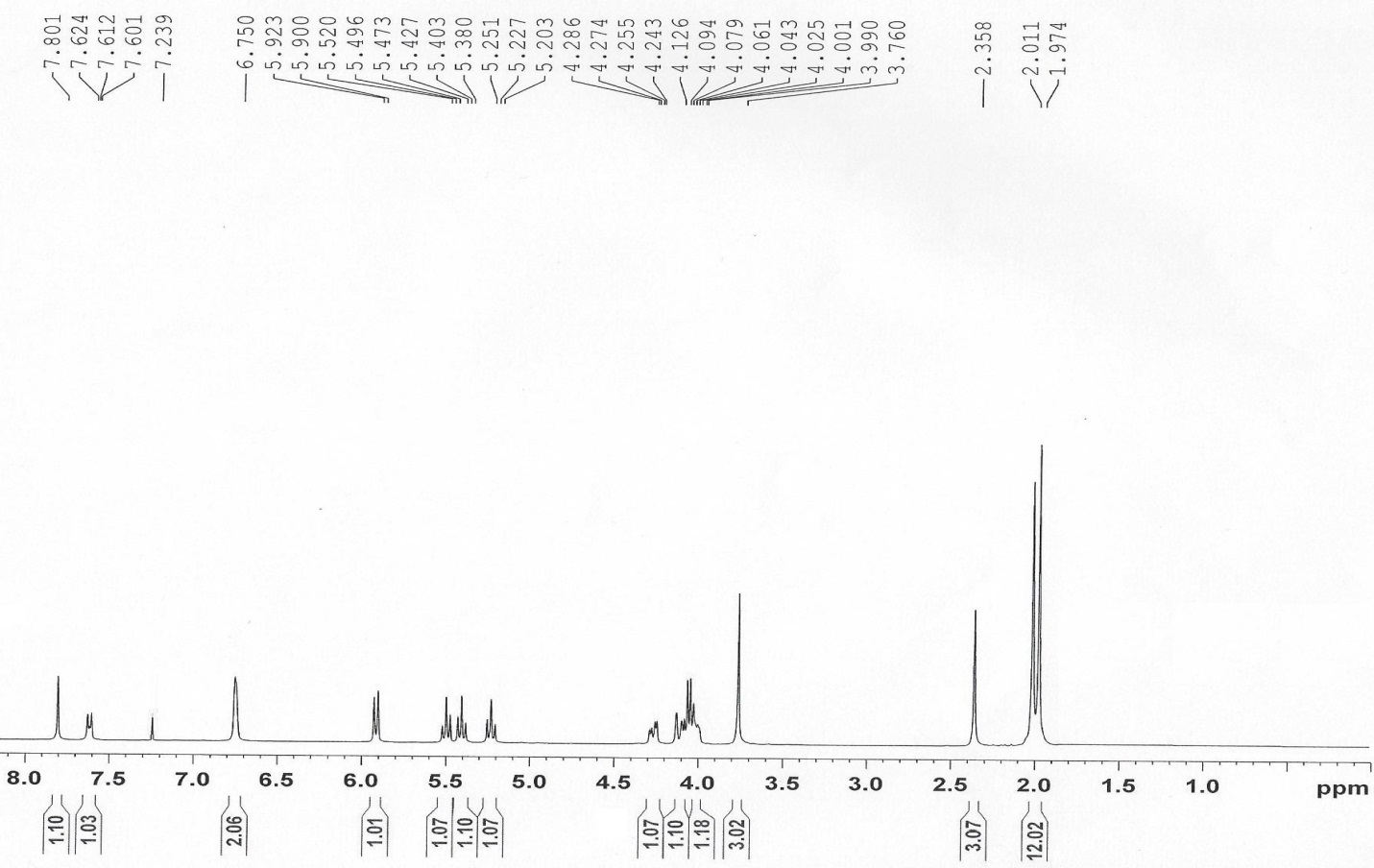


**Figure S67**. ^1^H NMR spectrum of **6ad** in CDCl_3_


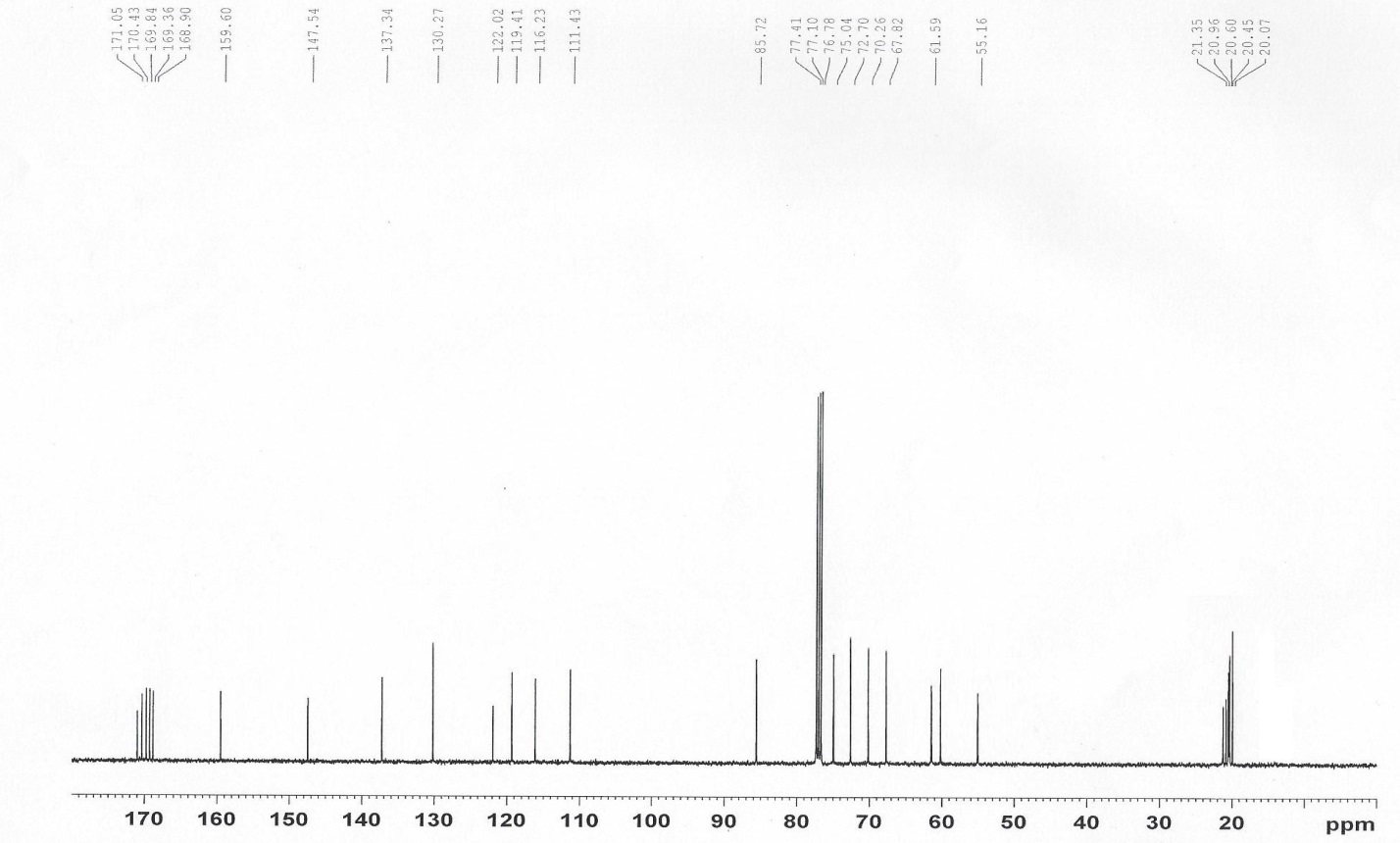


**Figure S68**. ^13^C NMR spectrum of **6ad** in CDCl_3_


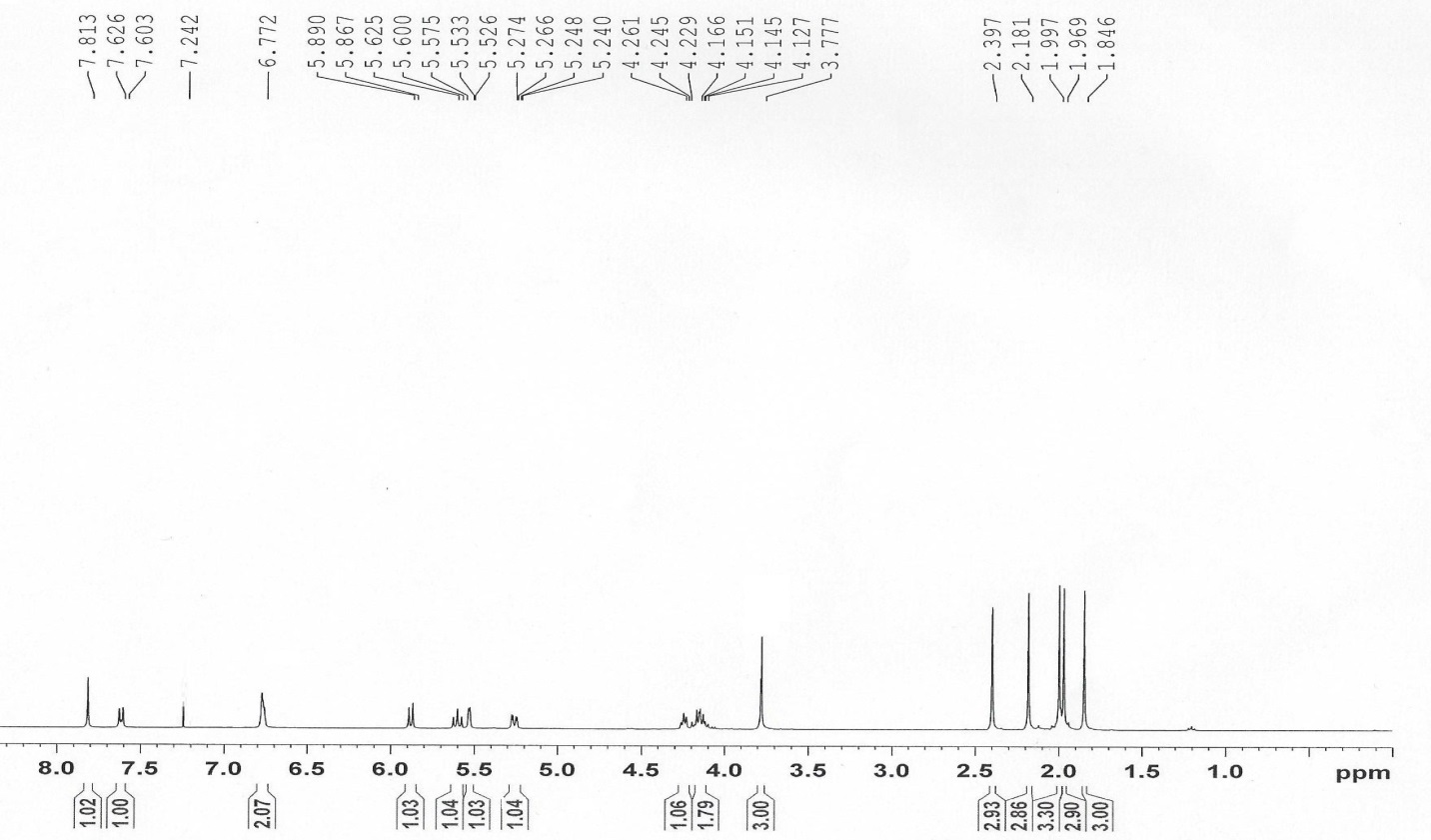


**Figure S69**. ^1^H NMR spectrum of **6bd** in CDCl_3_


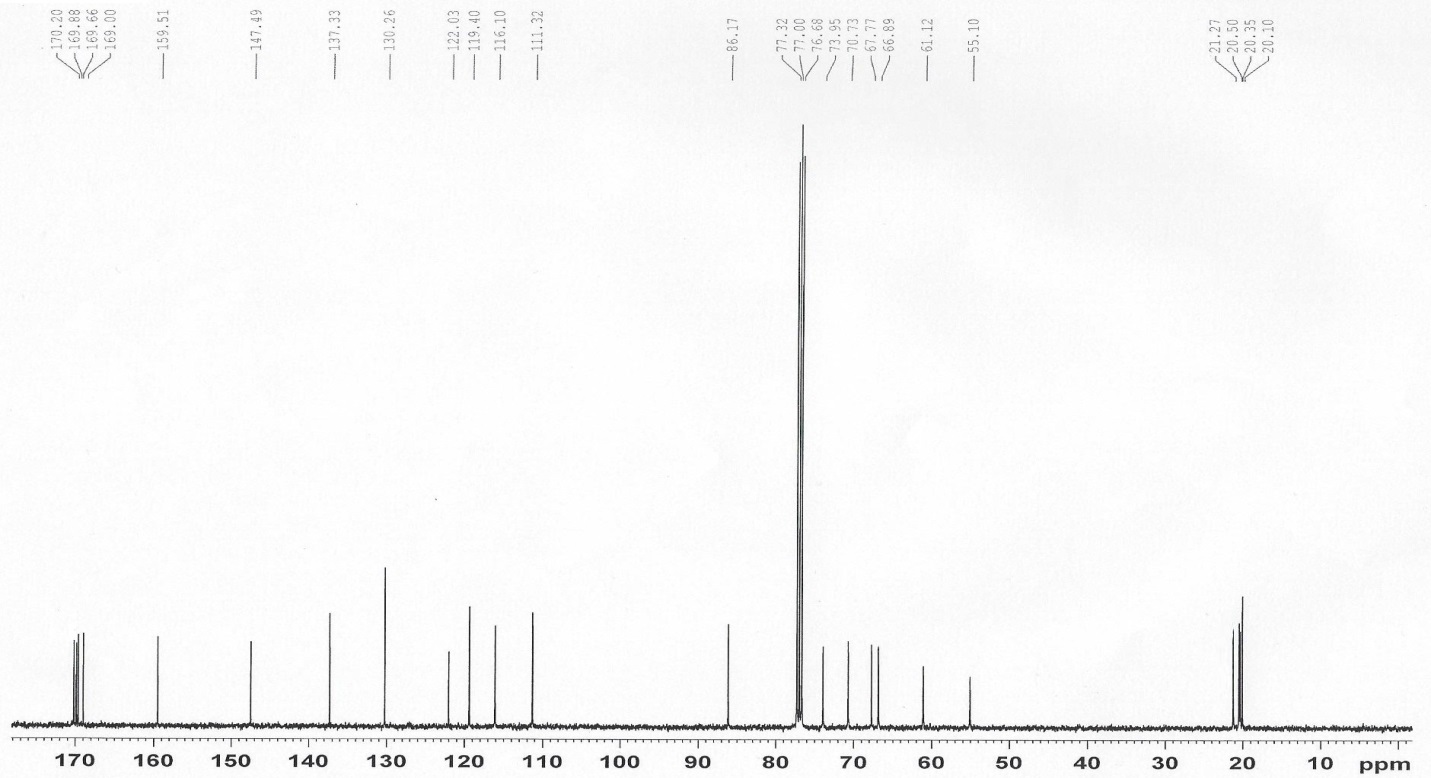


**Figure S70**. ^13^C NMR spectrum of **6bd** in CDCl_3_


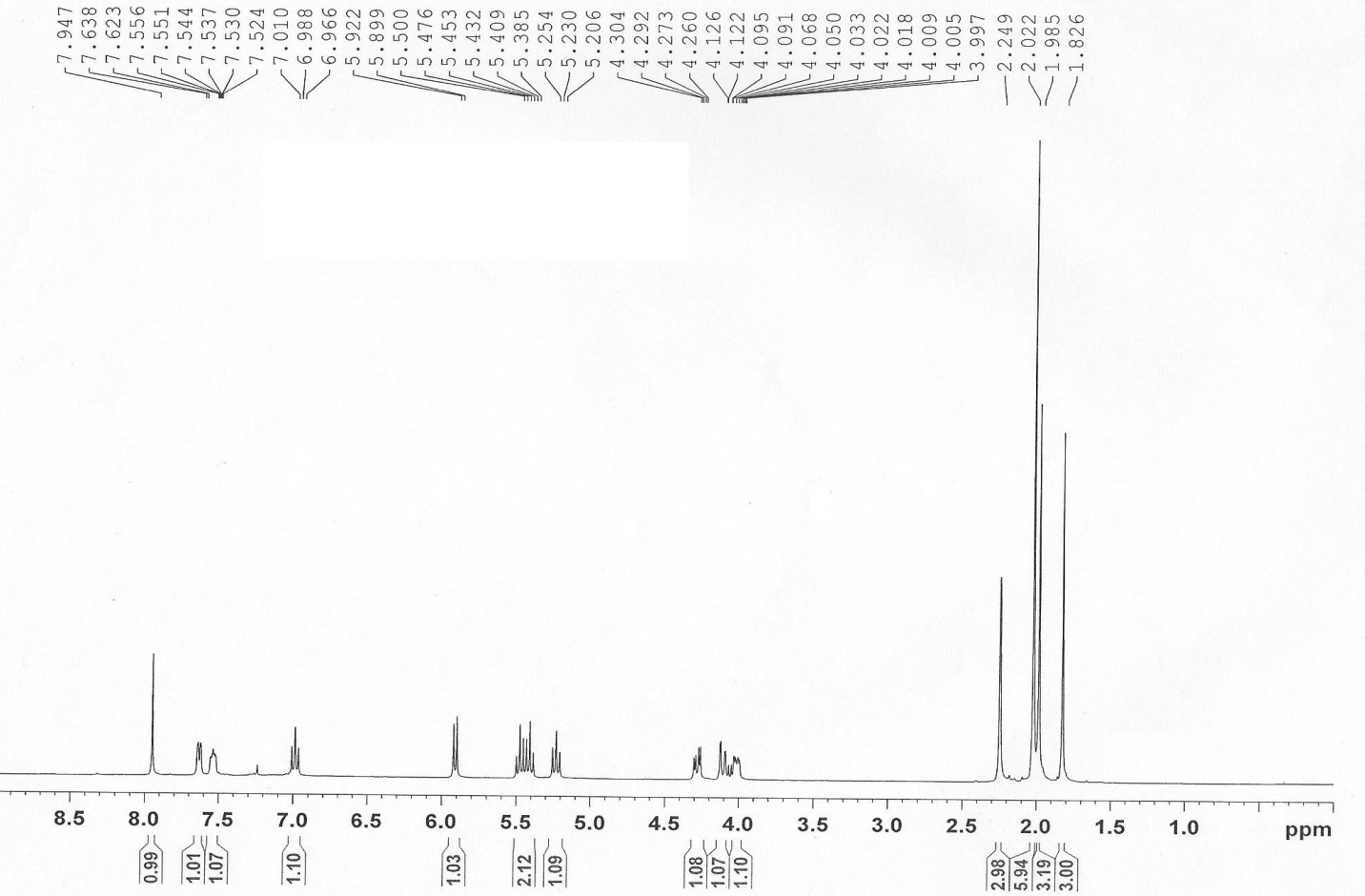


**Figure S71**. ^1^H NMR spectrum of **6ae** in CDCl_3_


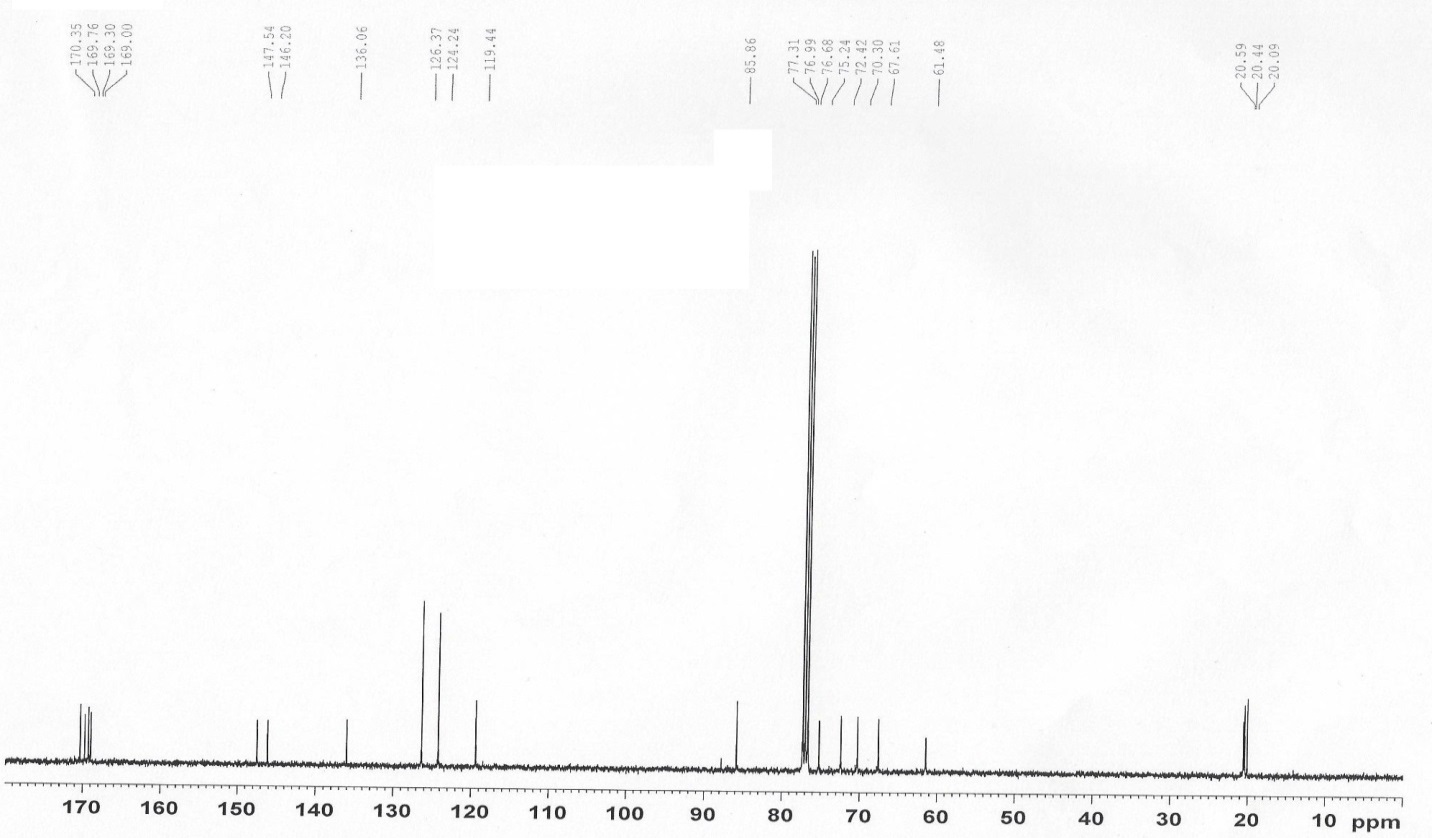


**Figure S72**. ^13^C NMR spectrum of **6ae** in CDCl_3_


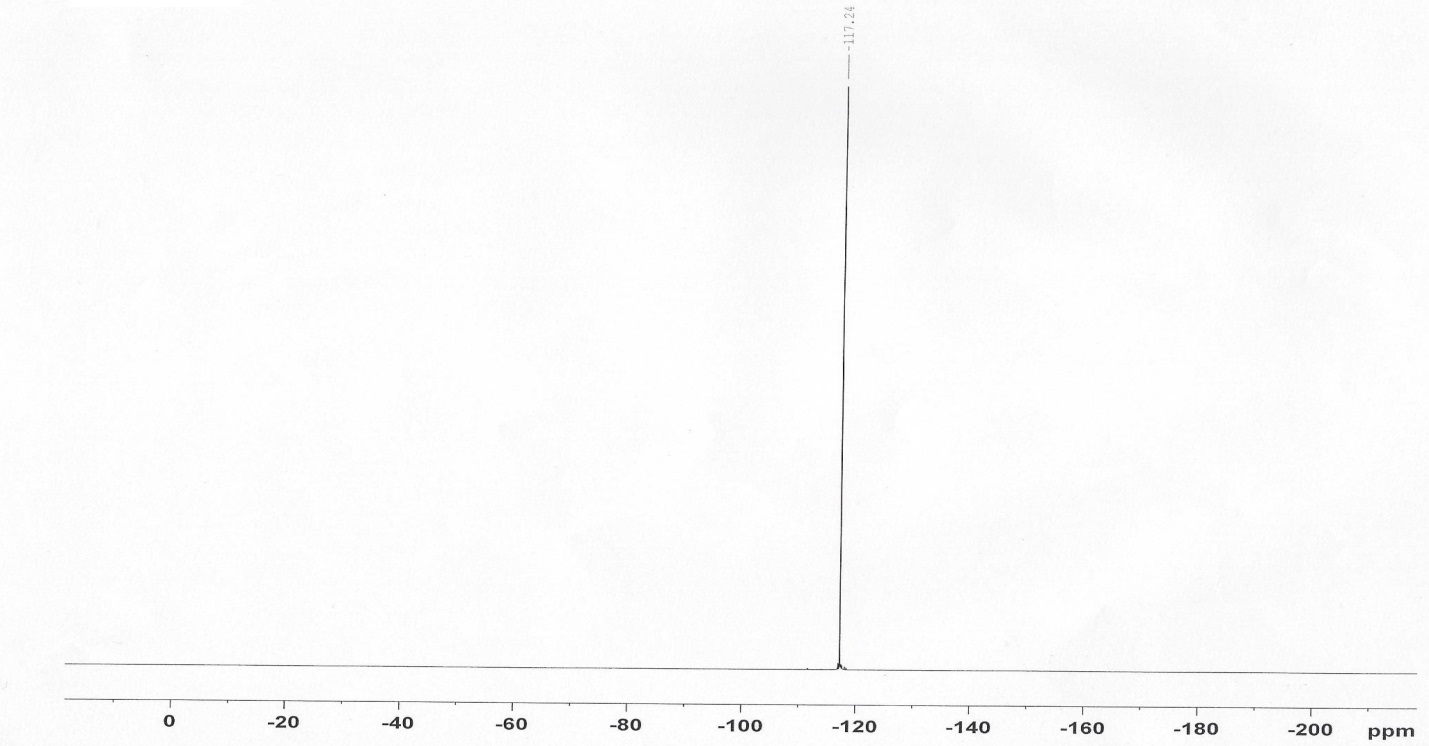


**Figure S73**. ^19^F NMR spectrum of **6ae** in CDCl_3_


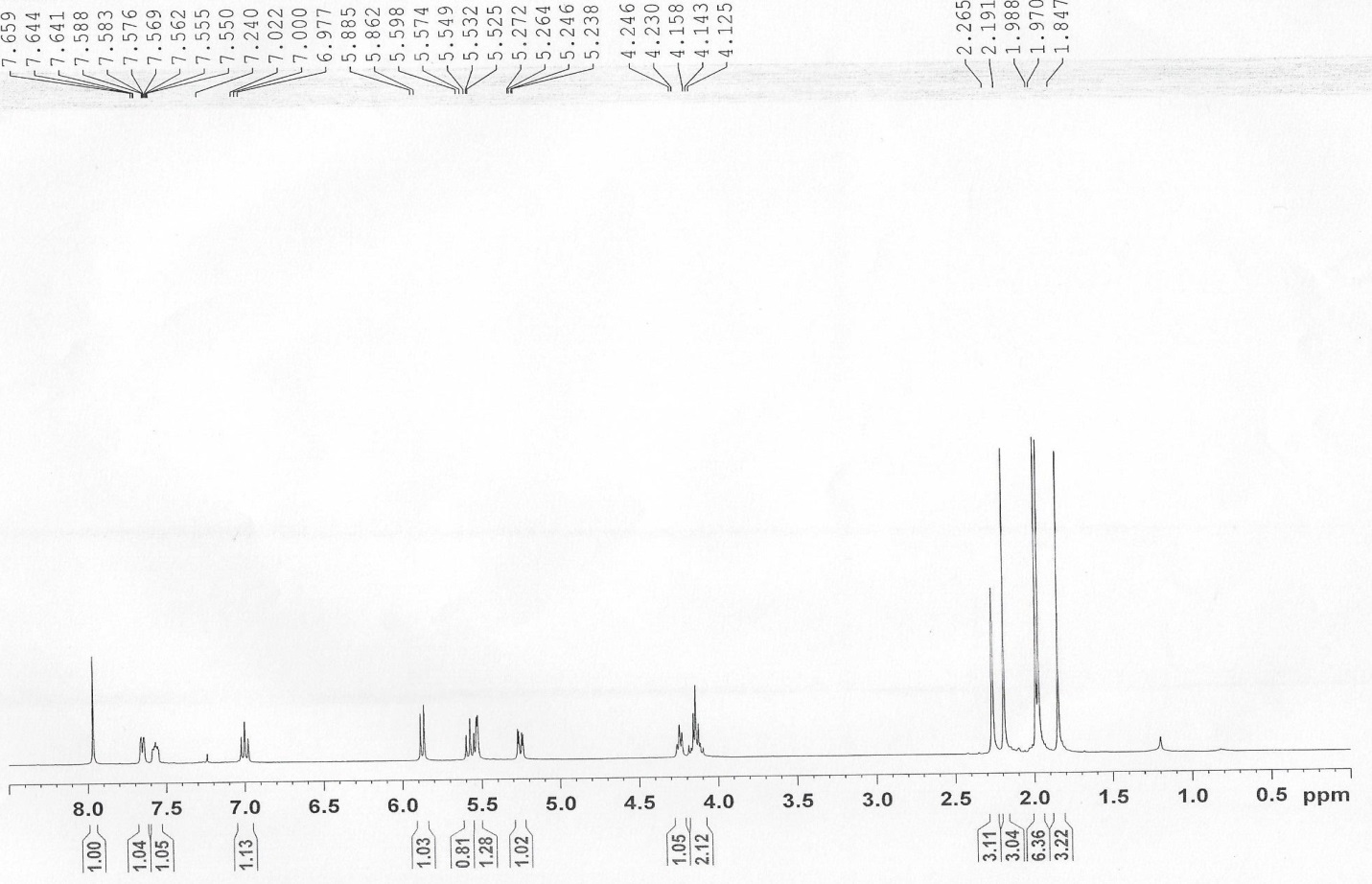


**Figure S74**. ^1^H NMR spectrum of **6be** in CDCl_3_


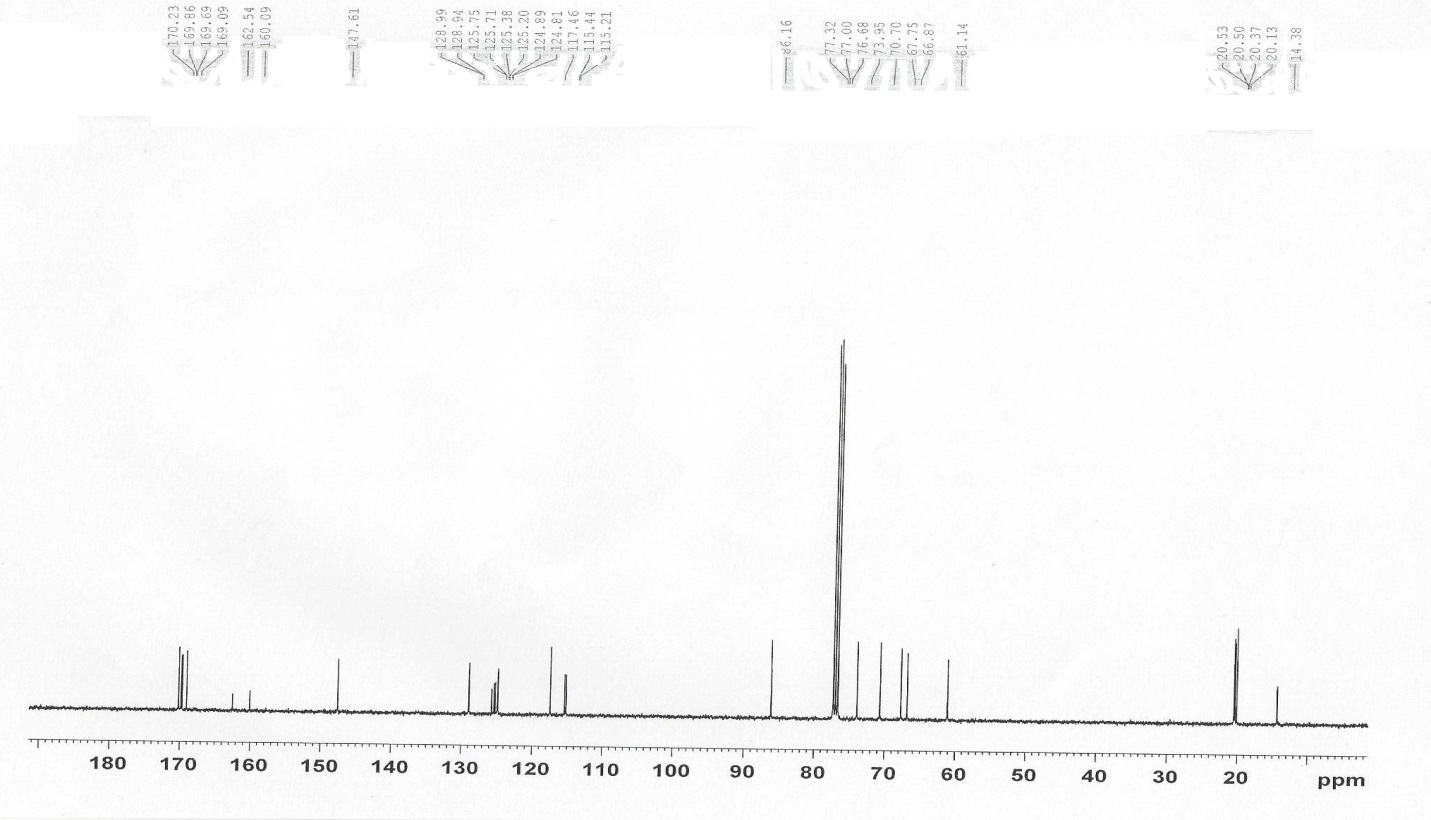


**Figure S75**. ^13^C NMR spectrum of **6be** in CDCl_3_


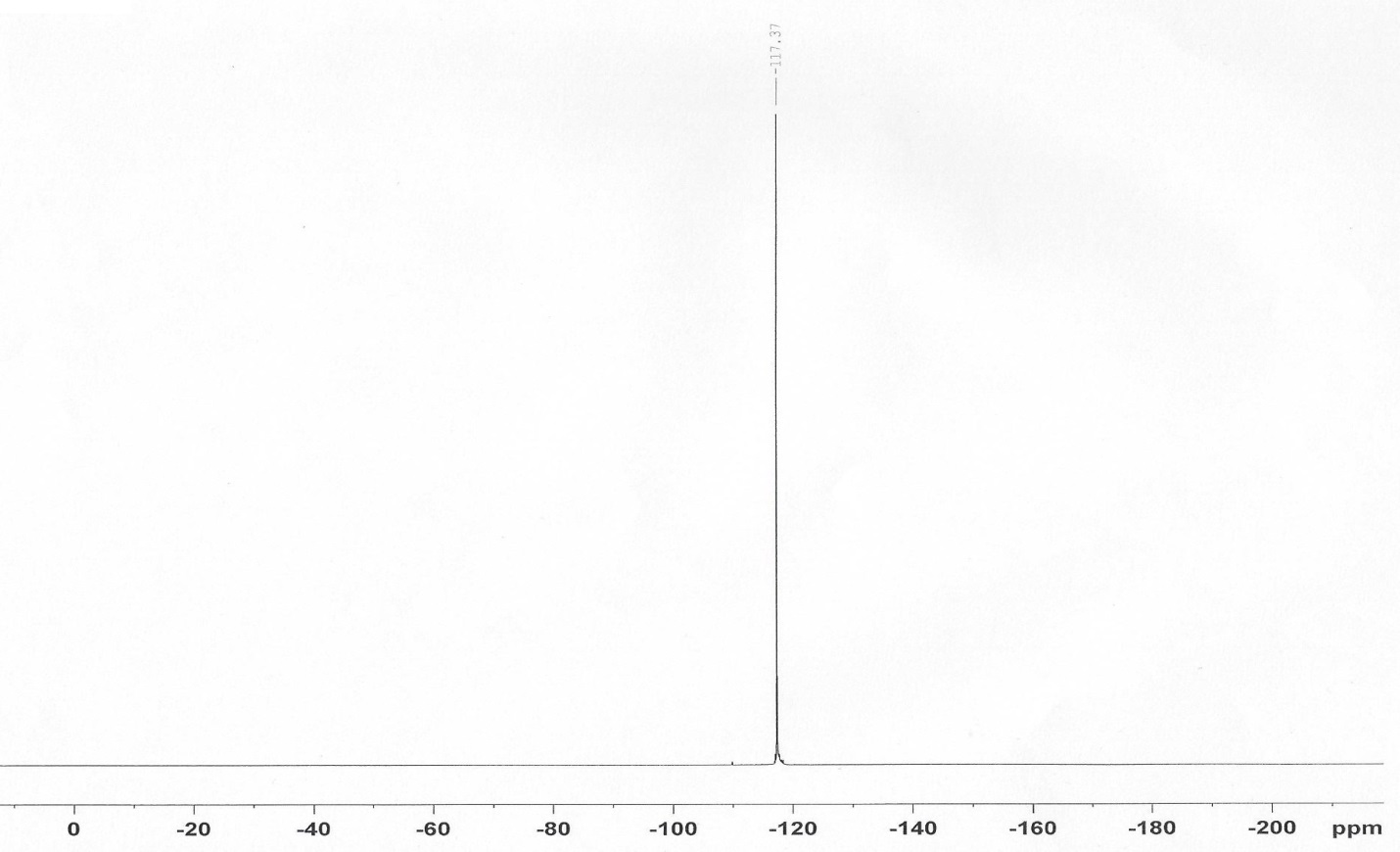


**Figure S76**. ^19^F NMR spectrum of **6be** in CDCl_3_


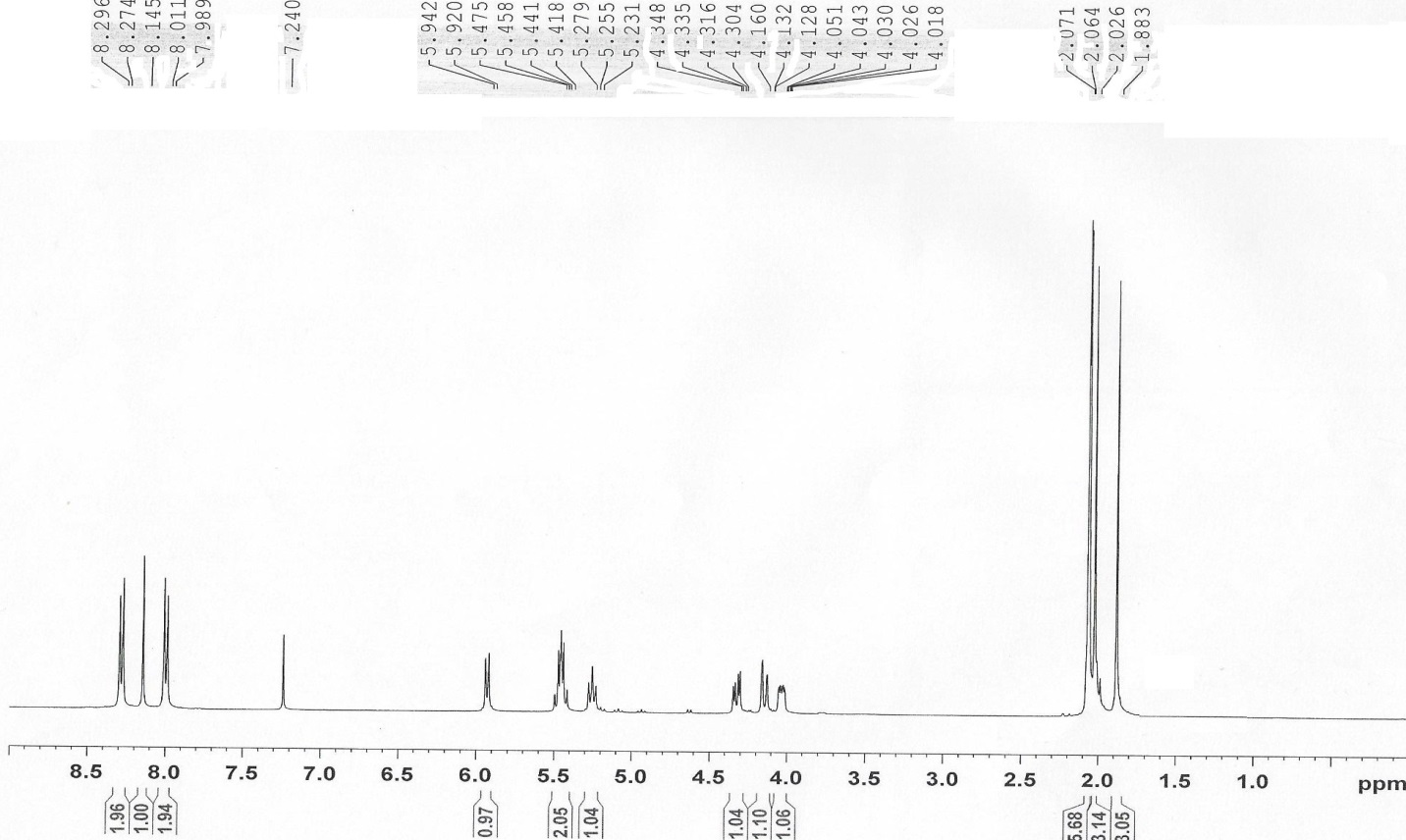


**Figure S77**. ^1^H NMR spectrum of **6aj** in CDCl_3_


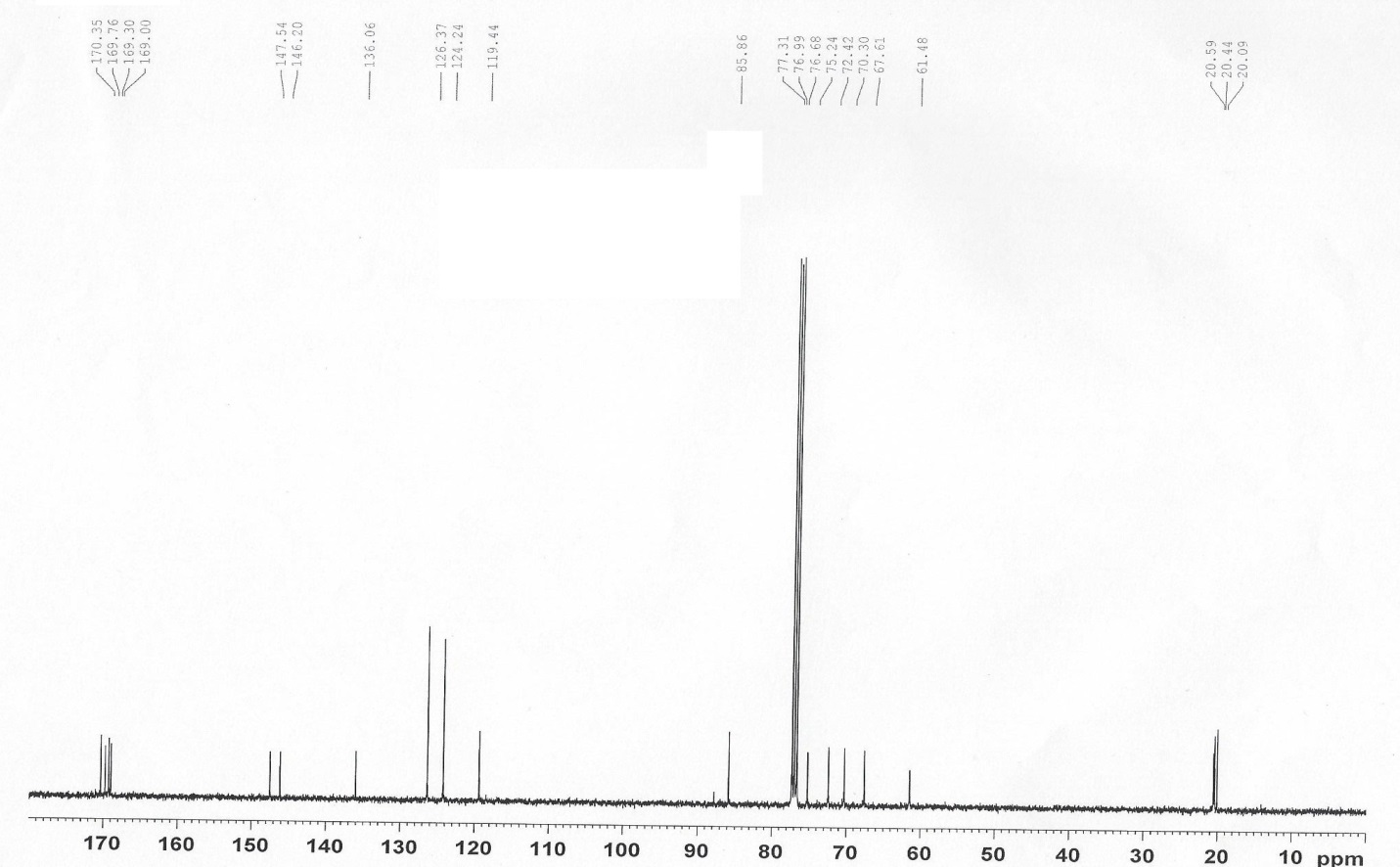


**Figure S78**. ^13^C NMR spectrum of **6aj** in CDCl_3_


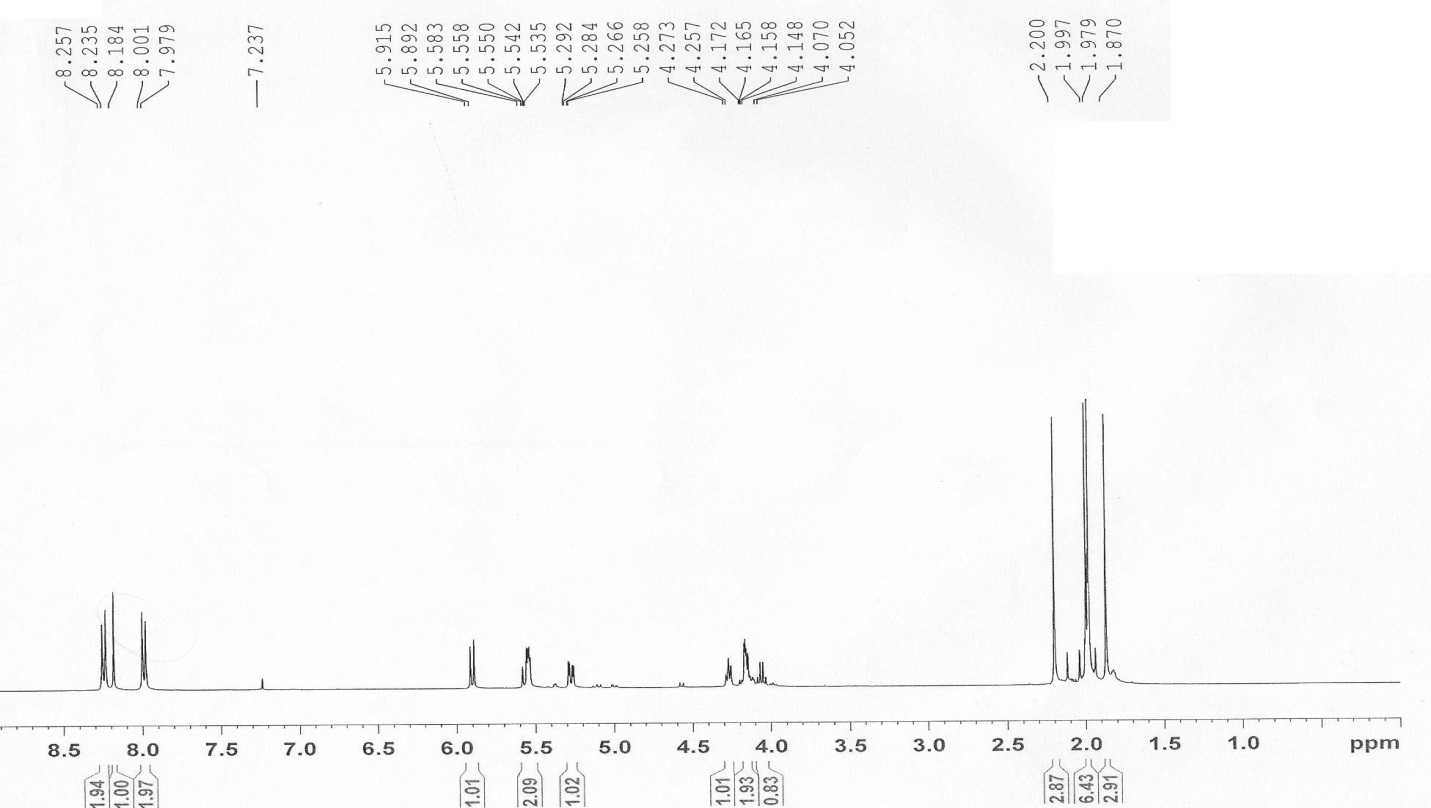


**Figure S79**. ^1^H NMR spectrum of **6bj** in CDCl_3_


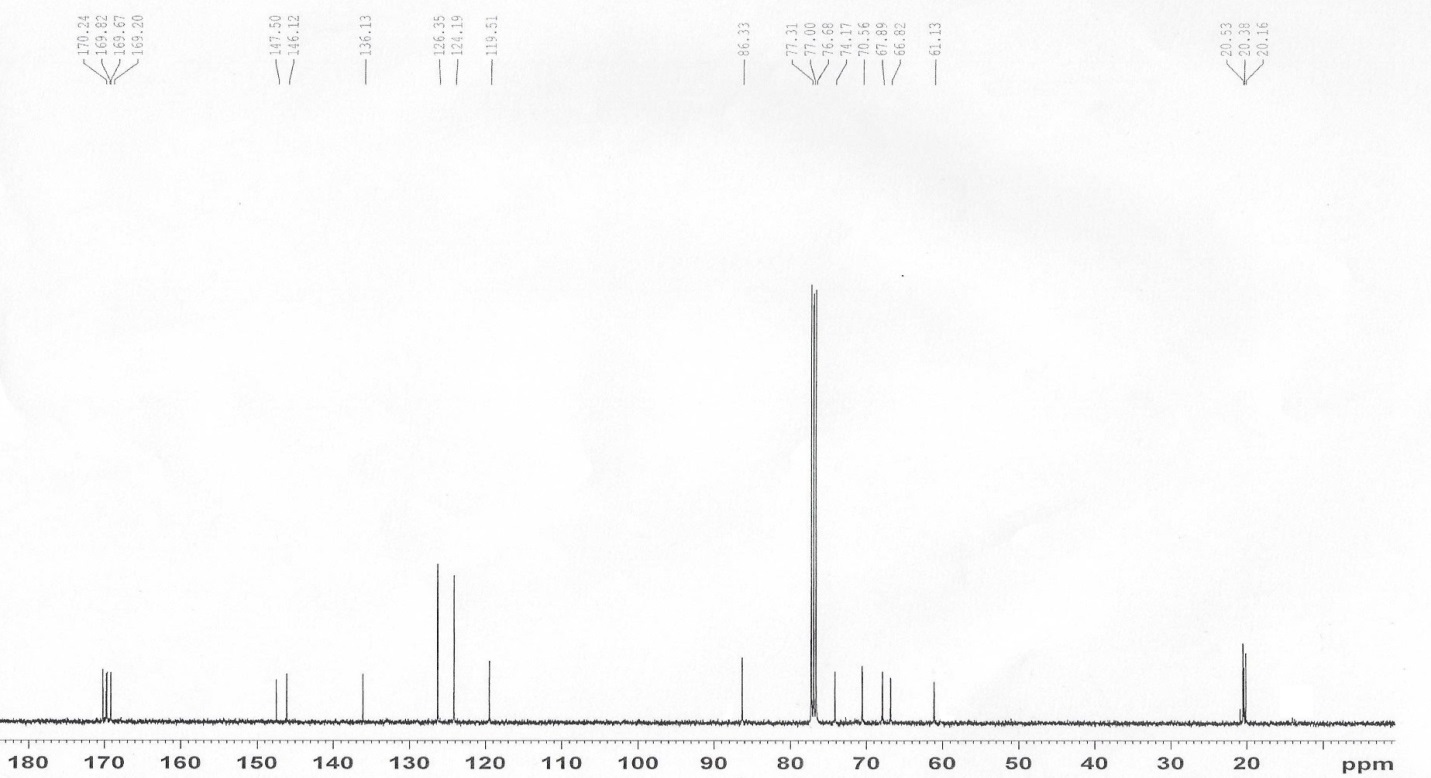


**Figure S80**. ^13^C NMR spectrum of **6bj** in CDCl_3_
